# Discovering genetic mechanisms underlying the co-occurrence of Parkinson’s disease and non-motor traits

**DOI:** 10.1038/s41531-024-00638-w

**Published:** 2024-01-23

**Authors:** Sreemol Gokuladhas, Tayaza Fadason, Sophie Farrow, Antony Cooper, Justin M. O’Sullivan

**Affiliations:** 1 The Liggins Institute, University of Auckland, Auckland, 1023 New Zealand; 2 Maurice Wilkins Centre for Molecular Biodiscovery, Auckland, 1010 New Zealand; 3 St Vincent’s Clinical School, UNSW Sydney, Sydney, NSW Australia; 4 Australian Parkinson’s Mission, Garvan Institute of Medical Research, Sydney, New South Wales Australia; 5 MRC Lifecourse Epidemiology Unit, University of Southampton, Southampton, UK; 6 Singapore Institute for Clinical Sciences, Agency for Science, Technology and Research (A*STAR), Singapore, Singapore

**Keywords:** Genomics, Parkinson's disease

## Abstract

Understanding the biological mechanisms that underlie the non-motor symptoms of Parkinson’s disease (PD) requires comprehensive frameworks that unravel the complex interplay of genetic risk factors. Here, we used a disease-agnostic brain cortex gene regulatory network integrated with Mendelian Randomization analyses that identified 19 genes whose changes in expression were causally linked to PD. We further used the network to identify genes that are regulated by PD-associated genome-wide association study (GWAS) SNPs. Extended protein interaction networks derived from PD-risk genes and PD-associated SNPs identified convergent impacts on biological pathways and phenotypes, connecting PD with established co-occurring traits, including non-motor symptoms. These findings hold promise for therapeutic development. In conclusion, while distinct sets of genes likely influence PD risk and outcomes, the existence of genes in common and intersecting pathways associated with other traits suggests that they may contribute to both increased PD risk and symptom heterogeneity observed in people with Parkinson’s.

## Introduction

Parkinson’s disease (PD) is clinically heterogeneous in nature, exhibiting a wide range of motor and non-motor symptoms that progress over time and differ between individuals^1^. Despite the substantia nigra being commonly considered the primary region affected in PD, the neurodegenerative process has been shown to significantly impact other brain regions, leading to a wide range of both motor and non-motor symptoms^2,3^ and traits. These include psychiatric symptoms^4^, reduced olfaction, sleep disorders, and dementia^5^. While the motor symptoms associated with PD are fairly well defined, the presentation of the non-motor symptoms in PD is extremely heterogeneous. This observed heterogeneity in symptoms associated with PD likely arises due to interactions between genetic, epigenetic, and environmental risk factors over an individual’s lifetime^6,7^. However, little is known about the mechanisms that underlie these non-motor associations.

Non-motor symptoms that include cognitive and executive dysfunctions are common in PD^8,9^. Prefrontal cortex dysfunction in early-stage PD with rapid eye movement sleep behaviour disorder has been proposed as an effective subtype-specific biomarker of neurodegenerative progression^10^ and non-motor functions. While the mechanisms attributing to the non-motor symptoms of PD remain unknown, altered connectivity that modifies the functional network organisation in the brain cortex was observed by EEG in PD patients when compared to age-matched controls^11^. This finding is consistent with cortical processes having a substantial role in PD. Some non-motor symptoms are frequently present during the prodromal (pre-motor) stage of PD^12^; thus, investigating their genetic and mechanistic underpinnings is critical for harnessing the non-motor aspect of PD for earlier diagnosis and intervention.

Additionally, the altered cortical function appears to be linked to early-stage compensation for basal ganglia dysfunction^10^ and, consequently, motor functions. Given its association with non-motor and motor symptoms in PD, the question remains whether inherited common genetic variants associated with gene expression changes in the brain cortex can be causally linked to the development of PD. Mendelian Randomization (MR) is a powerful approach that determines the causal relationship between modifiable gene expression and disease outcomes using genetic variants associated with gene expression as instrumental variables (IVs)^13^. MR has previously been used to identify genes whose expression changes in dopaminergic neurons have a causal role in PD^13^. However, given that gene expression and its regulation are predominantly tissue-specific, identifying PD risk factors in the brain cortex and exploring the biological mechanisms that they affect may provide valuable insights into cortical mechanisms contributing to PD and co-occurring traits, including non-motor symptoms.

In addition to the motor and non-motor symptoms associated with PD, a number of traits can co-occur with PD. Understanding the complex connections between PD and co-occurring traits requires integrating information across biological levels (e.g. genetic risk, chromatin structure, protein interactions and phenotypes) to inform on the mechanistic interactions that impact shared inherited genetic risk. We have developed a de novo analytical approach that uses protein-protein interaction networks (PPIN) to identify connections across these levels^14–17^. The use of tissue-specific gene regulatory networks (GRN) within this integrative approach identifies conditions/traits co-occurring with PD and the genetic variation and biological pathway(s) that are responsible for the observed co-occurrence.

Here, we performed an unbiased, de novo analysis^14,15,17,18^ integrating the Brain Cortex-specific Gene Regulatory Network (BC-GRN), PPIN and GWAS data. We identify SNPs (that are associated with altered expression of specific genes) in the brain cortex that are causally related to PD. Seeding protein interaction networks with the genes whose expression is altered, and in parallel with genes regulated by PD-associated SNPs, identifies proteins and additional genetic variants that likely explain the genetic basis of a number of traits associated with PD. The finding that these genetic variants are common in diverse populations likely explains why the occurrence varies between individuals. We contend that these findings provide mechanistic insight for identifying therapeutics to treat a subset of the largely untreated non-motor conditions associated with PD and for whom the individuals can be readily identified. Downstream clinical trials that include genetic stratification are essential for confirming these findings and targeting the non-motor symptoms of PD, particularly those that occur in the prodromal phase.

## Results

### Mendelian randomization (MR) identified 19 genes whose altered expression in the brain cortex was causally linked to PD

**Fig. 1:**
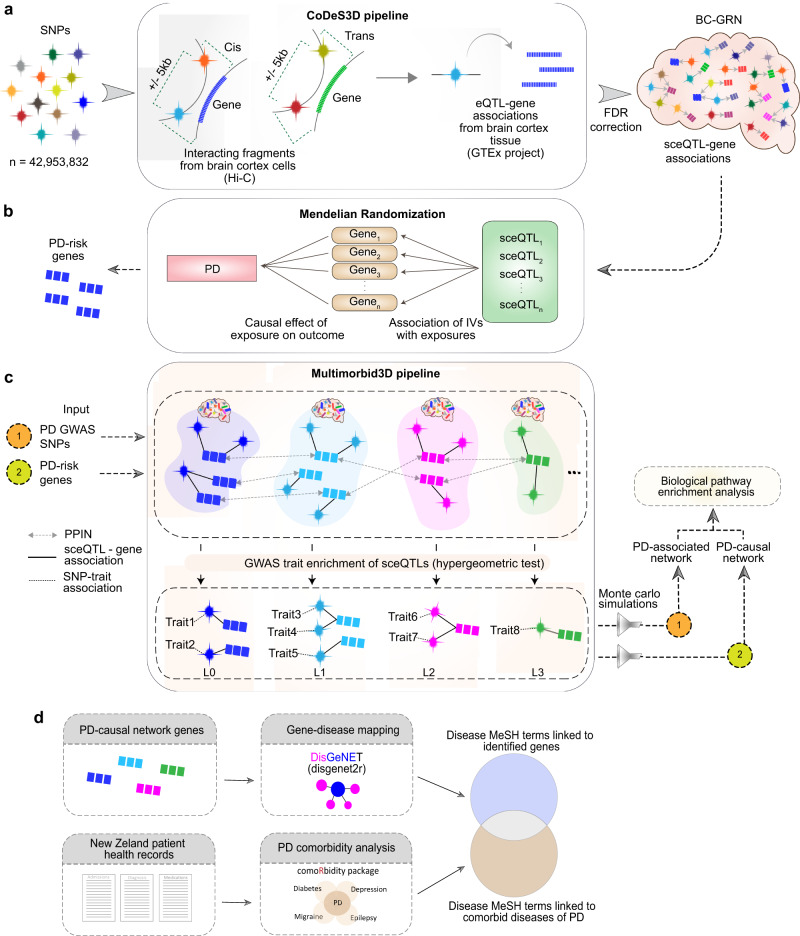
Overview of the study. **a** The CoDeS3D pipeline^56^ was used to construct the Brain Cortex Gene Regulatory Network (BC-GRN) by integrating Hi-C data from cortex cells and eQTL data from brain cortex tissue to map common SNPs to their target genes. The sceQTL-gene associations that passed the Benjamini–Hochberg FDR correction threshold constitute the BC-GRN. **b** Transcriptome-wide Mendelian randomization (MR) was used to estimate the causal effect of the expression of the brain cortex genes (exposures) on PD using sceQTLs as IVs. **c** The multimorbid3D pipeline was used to connect the PD-risk genes (identified by MR analysis in **b**) and PD-associated GWAS SNPs (Nalls et al., 2019) to co-occurring traits and the genetic and biological interaction that connects them (see ‘Methods’). Biological pathways enriched for the PD-causal and PD-associated network were detected. **d** Medical conditions that co-occur with PD (ICD10 code – G20) were identified within the clinical records of ~2 million NZ public health patients (January 2016 and December 2020) using the comorbidity R package. ICD10 codes for co-occurring conditions were converted to MeSH terms using the Unified Medical Language System application programming interface. The MeSH terms for the observed co-occurring conditions were compared to the MeSH terms for the genes that are linked to PD risk, following disease-gene mapping (R interface of DisGeNET (*disgenet2r*^64^)).

**Fig. 2:**
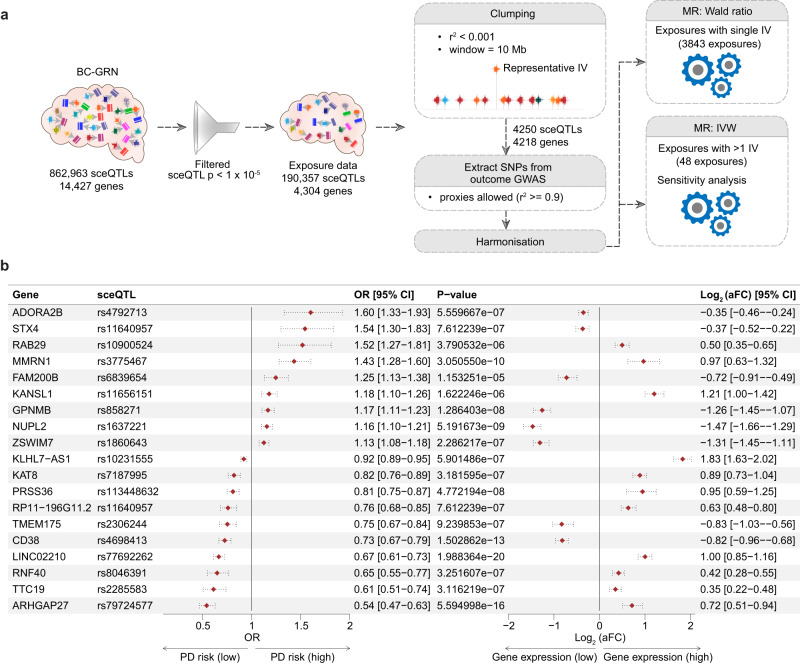
Identification of PD-risk genes in the brain cortex using MR. **a** The sceQTL-gene associations within the BC-GRN with an association *p*-value (<1 × 10^−5^) were included in the exposure dataset. Clumping identified independent sceQTLs (IVs) for each gene (exposure). IV summary statistics were extracted from the outcome GWAS^19^. Proxy SNPs (LD; r^2^ >=0.9) were used for IVs that were absent in the outcome GWAS. The exposure and outcome data were harmonised to ensure that the risk was conferred by the same effect allele in both datasets. Finally, MR analysis was performed using Wald ratio and Inverse variance weighted (IVW) methods for exposure with single IV and >1 IV, respectively. **b** Genes identified as having significant PD risk (*p* < 1.15e−05). The Forest plot: left, the odds ratio (OR) of PD per 1-SD change in gene expression in the brain cortex; and right, allele-specific gene expression changes (aFC) associated with the sceQTL in the brain cortex. Horizontal dotted lines represent a 95% confidence interval.

Changes in gene expression associated with germline variants in the brain cortex may contribute to the development of PD, and investigating these changes could provide new insights into disease mechanisms underlying PD. Therefore, we aimed to identify gene regulatory interactions in brain cortex that may be causally linked to PD. We constructed a Brain Cortex Gene Regulatory Network (BC-GRN) that consisted of 1,050,132 sceQTLs associations between 862,963 sceQTLs and 14,427 genes (Fig. 1a; Supplementary Fig. 1). From the BC-GRN, regulatory interactions involving 4304 genes and 190,357 sceQTLs with association *p* < 1e−5 and FDR < 0.05 were considered for MR analysis (see ‘Methods’). Using the selected sceQTLs as instrumental variables (IVs), we estimated the causality of their target genes (exposures) on PD using a two-sample MR approach (Figs. 1b and 2a). Of 3968 genes tested, changes in the expression of 19 genes (three non-coding and 16 protein-coding genes) were identified as causally related to PD at a stringent threshold (*p* < 1.28e−05), hereafter referred to as “PD-risk” genes (Fig. 2b). Expression changes (allelic fold change (aFC)) for the 19 PD-risk genes were associated with 18 unique sceQTLs in the brain cortex. Among these 19 PD-risk genes, nine increase and ten decrease the odds ratio (OR; risk) of PD. For example, increased expression of *ARHGAP27* and *RNF40* in the brain cortex was associated with decreased OR of PD. By contrast, cortical down-regulation of *FAM200B, GPNMB*, and *NUPL2* (HGNC official name *NUP42*) was associated with increased OR of PD. In addition to the 19 PD-risk genes, 300 genes were identified as being suggestive (1.28e−05 > *p* < 0.05) of causal associations with PD, hereafter referred to as “suggestive PD-risk genes” (Supplementary Table 1). Pathway enrichment analysis did not return any biological pathways enriched for these suggestive PD-risk genes.

### Identification of traits that co-occur with PD

Given that genes/proteins don’t function in isolation, it is important to consider additional regulatory and interaction networks for the 19 PD-risk genes under the premise that regulatory or interaction effects may modify the impact of these genes on PD risk. With this in mind, we used the BC-GRN to map additional regulatory sceQTLs, in addition to the 18 causal sceQTLs, for each gene on each level of the protein interaction network and tested the sceQTLs for enrichment in GWAS traits to create the “PD-causal network” (Fig. 1c). Similarly, we created a separate “PD-associated network”; that is, the PPIN expanded from the genes that are regulated by PD-associated SNPs^19^ (i.e. GWAS SNPs; Supplementary Table 2) and their regulatory sceQTLs in the cortex. To identify co-occurring traits of PD, the PPIN was expanded up to five levels in PD-causal and -associated networks (Fig. 1c). However, subsequent analyses of the PD-causal and PD-associated networks were restricted to three interaction levels (L0-L3) to minimise the risk of including traits that co-occur with PD by chance (Supplementary Fig. 2), resulting in 53 and 93 genes within the PD-causal and PD-associated networks, respectively (Supplementary Tables 3 and 5).

**Fig. 3:**
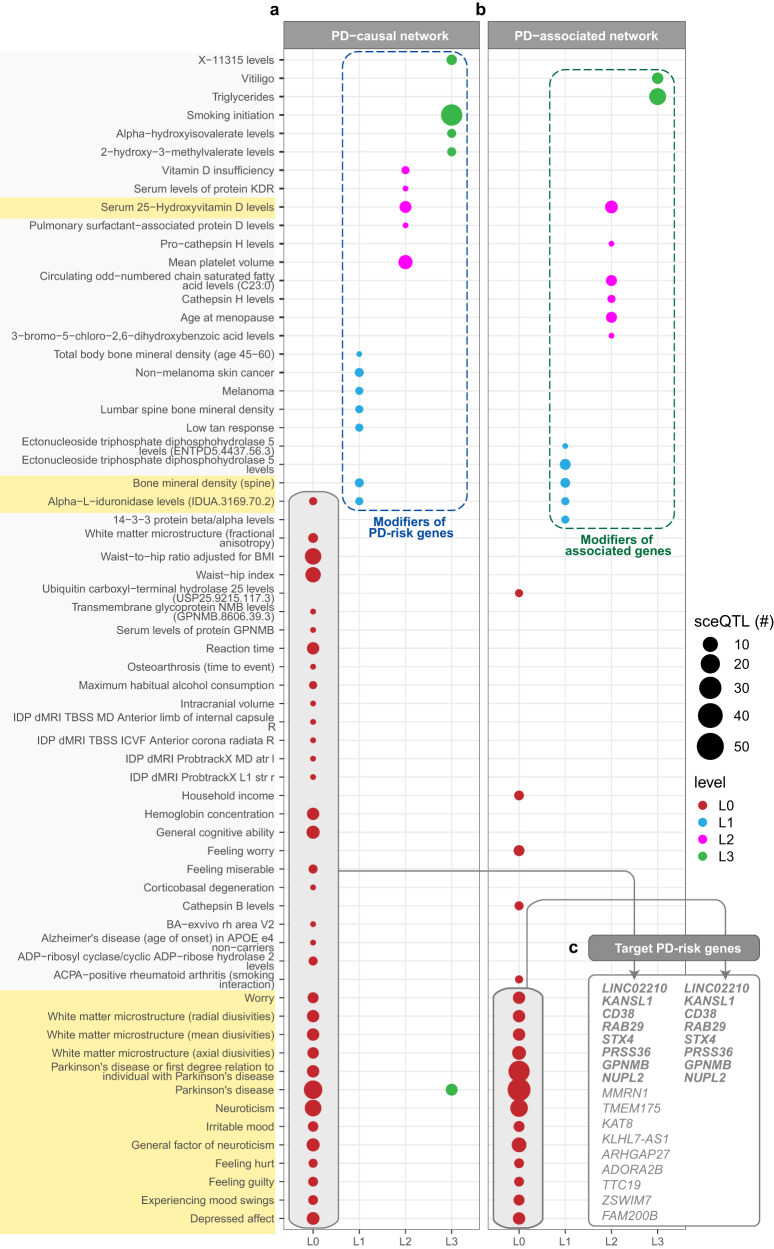
PPI networks seeded with PD-risk genes and associated GWAS SNPs identify traits that co-occur with PD. Traits-associated loci enriched within **a** PD-causal network and **b** PD-associated network. Traits identified at L0 of the PD-causal network are inferred to be comorbid traits of PD, whereas those identified at L0 of the PD-associated network are PD-associated traits. Regulatory loci associated with traits identified at L0-L3 of the PD-causal (blue dotted lines in **a**) and PD-associated (green dotted line in **b**) networks are modifiers of the PD-risk and PD-associated genes, respectively. Traits associated with both PD-causal and PD-associated networks are highlighted in yellow. **c** Target PD-risk genes of the regulatory loci associated with L0 traits within the PD-causal network and PD-associated network. Genes highlighted in bold font are PD-risk genes.

Forty-nine traits were significantly enriched within the PD-causal network (Fig. 3a; Supplementary Table 3). Two of the 49 traits were PD and proxy-PD (first-degree relative), which is consistent with the identification of the genes (*LINC02210, KANSL1, TMEM175, CD38, MMRN1, RAB29, STX4, PRSS36, GPNMB, NUPL2, KAT8, KLHL7-AS1*, and *ARHGAP27*) being causally linked to PD, as the loci containing the regulatory sceQTLs have previously been associated with PD risk through GWAS. The identification of PD on level three is due to eQTLs regulating *WNT3*, *SETD1A*, and *AP2B1* and is consistent with the hypothesis that modifiers of the PD-risk genes can be detected. Smoking initiation was a level three trait associated with a set of 47 SNPs associated with 19 genes (Supplementary Table 4), five of which (*AP2B1, ERBB2, GAB2, PTK2, RAF1*) are enriched in the EGF/EGFR signalling pathway (WikiPathways: WP437, *p* = 1.716 × 10^−3^) (Supplementary Table 6). Given that the regulatory loci associated with L0 traits (Fig. 3a—grey box) directly modulate the PD-risk genes, it can be inferred that these traits are “comorbid” with PD, add to the patient’s symptoms, and they may additionally worsen the disease trajectory. In contrast, the regulatory loci associated with traits spanning L1-L3 within the PD-causal network (Fig. 3a—blue dashed box) influence PD-risk genes by acting as modifiers of genes (proteins) that interact with the PD-risk gene encoded proteins. It is important to remember that the tissues affected by the traits associated with the sceQTLs are not restricted to the brain cortex. As such, it is possible that these traits mechanistically modify the disease trajectory of PD.

Thirty-one traits were significantly enriched within the PD-associated network (Fig. 3b; Supplementary Table 5). A subset of regulatory loci associated with traits at L0 of the PD-associated network also regulate PD-risk genes (Fig. 3c). The majority of these shared GWAS traits are either brain-related phenotypes or psychiatric traits (e.g. depressed affect, irritable mood, etc.). Further, 16 of the 31 traits enriched within the PD-associated network (Fig. 3, highlighted in yellow) were also present within the PD-causal network, consistent with convergence between the causal and associated networks. We hypothesised that this observation might extend to convergence between common genetic variation associated with PD and suggestive PD-risk genes. Therefore, we tested to see if the genes that are within the PD-associated network overlapped the suggestive PD-risk genes from the MR analysis to predict alternate converging pathways that influence PD and associated symptoms. Of the 93 genes in the PD-associated network (Supplementary Table 5), ten genes (*FAM47E, PCGF3, CTSB, SH3GL2, WNT3, ITGA8, IP6K2, P4HTM, BIN3*, and *AREL1*) were found to overlap with the set of 300 suggestive PD-risk genes.

### 17q21.31 genes contribute to the interaction between PD and mood-related traits

**Fig. 4:**
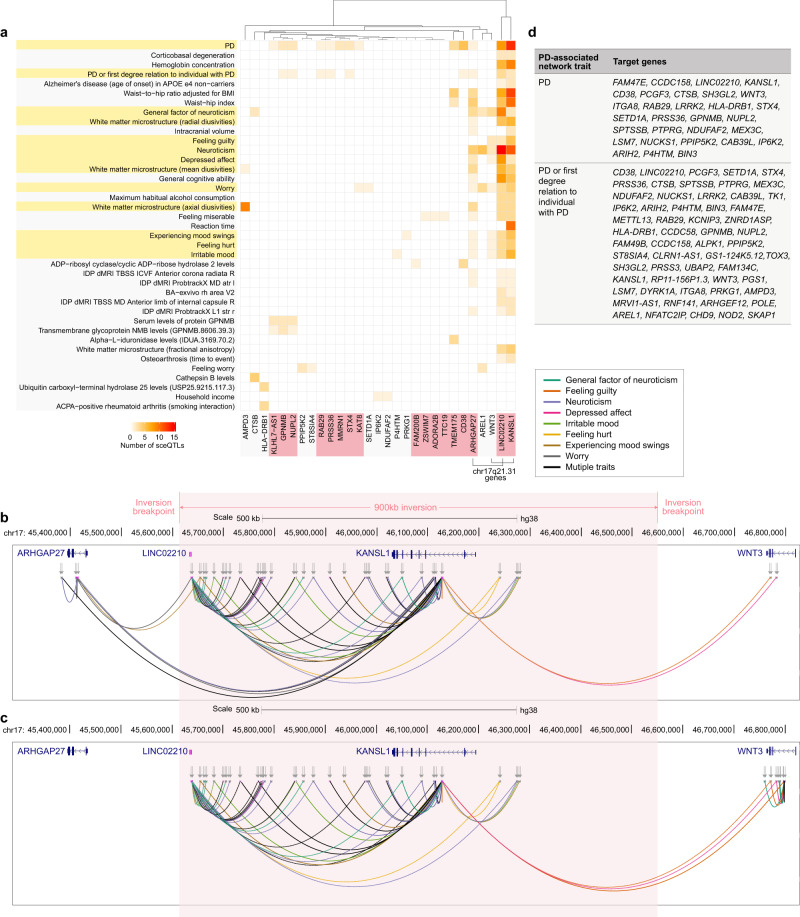
Interaction of L0 traits and PD is primarily mediated by 17q21.31 genes. **a** Regulatory loci associated with mood-related traits at L0 of the PD-causal and PD-associated networks (Fig. 3) predominantly regulate 17q21.31 genes (i.e. *KANSL1, LINC02210, WNT3, ARHGAP27*). Overlapping traits between PD-causal and PD-associated networks are highlighted in yellow, and the PD-risk genes are highlighted in pink. **b**, **c** The regulatory interactions across 17q21.31 are illustrated for the PD-causal network and the PD-associated network, respectively. Line colours correspond to the trait that is associated with the sceQTL that contributes to the interaction. Inverted arrows show the chromosomal location of sceQTLs. Inversion boundaries are diffuse but are located between *ARHGAP27* – *LINC02210* and *KANSL1*-*WNT3*. The limits of the inverted region are shaded in pink. **d** Genes that were associated with PD and proxy-PD-associated GWAS loci (Nalls et al., 2019) within PD-associated network.

In both the PD-causal and PD-associated networks, we identified regulatory loci associated with PD and brain/mood-related traits (e.g. worry, depressed effect, feeling miserable) that predominantly regulate gene expression in the 17q21.31 region (Fig. 4a). This suggests that the 17q21.31 locus may contribute to the relative risk of non-motor mood-related traits in PD, consistent with previous findings highlighting this region’s importance for cognitive and mood-related traits^20^. Most of these mood-related trait-associated regulatory loci are located within the 900 kb inversion breakpoint within the 17q21.31 locus and exert their regulatory activity through *cis-*regulatory interactions (Fig. 4b, c). Furthermore, we found that most of these regulatory interactions at the 17q21.31 locus are shared between both the PD-causal (Fig. 4b) and PD-associated networks (Fig. 4c). Notably, *LINC02210* and *KANSL1* (located within 17q21.31 inversion) are identified in both the PD-causal and PD-associated networks, while *ARHGAP27* is not within the PD-associated network (Fig. 4d).

### Shared biological pathways link PD and co-occurring traits of PD

**Fig. 5:**
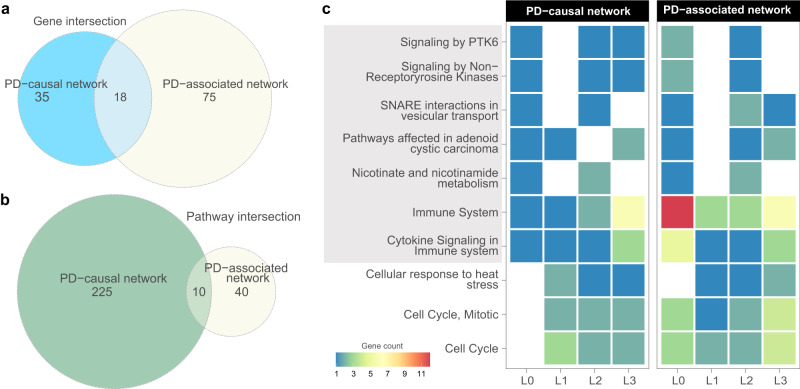
Genes from the PD-causal and PD-associated networks function within a subset of biological pathways. **a** The Venn diagram demonstrates the overlap of genes between the PD-causal network and PD-associated network identified across L0-L3. Comparatively, the number of intersecting genes is smaller when considering the size of each individual network. **b** Similarly, only ten biological pathways were found to overlap between the pathways enriched for PD-causal network genes and PD-associated network genes. **c** The figure shows the number of genes from each level of PD-causal and PD-associated networks enriched for each intersecting pathway. Of 10 intersecting pathways, seven (highlighted in grey) contain PD-risk genes.

Mapping PD-risk and PD-associated genes to biological pathways is vital to gain insights into pathogenic mechanisms and understand the relationships between PD and other traits. Therefore, we performed pathway enrichment analysis and identified the biological pathways over-represented by genes within the PD-causal network (Supplementary Table 6) and PD-associated network (Supplementary Table 7). Although 18 genes appear in both the PD-risk and PD-associated networks (Supplementary Table 8), only ten pathways were shared between them (Fig. 5c; Supplementary Table 9). The majority of genes and the biological pathways enriched for those genes are distinct and do not overlap within these two networks. Of note, PD-risk genes are enriched within shared pathways alongside genes from subsequent levels of the PD-causal and associated networks. This focused (predominantly involving a small number of genes associated with many traits [compare Fig. 5a, b]) analysis leads to the observation that traits associated with genes at levels L1 to L3, along with their regulatory loci, may exert their influence on PD through interconnected biological pathways (Fig. 5c).

### Diseases associated with PD-causal network genes overlap with comorbid medical conditions in PD patients

**Fig. 6:**
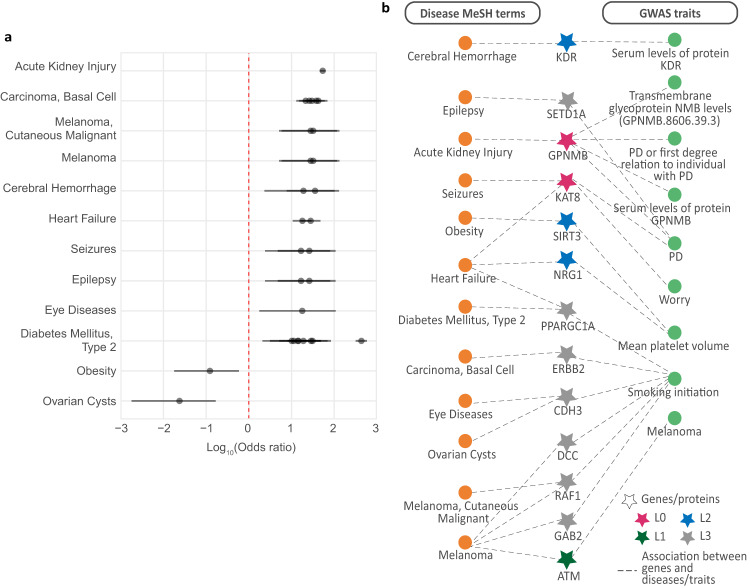
Patient records confirm that diseases associated with the genes identified within the PD-causal network co-occur with PD. **a** Mesh terms of diseases comorbid with PD in NZ. Odds ratios (OR) were calculated for the ICD10 codes for co-occurring conditions that overlapped gene-related medical conditions identified using DisGeNET data. Diseases with multiple ORs represent three-letter ICD10 categories or MeSH subheadings with more than one sub-category. For example, Melanoma (MeSH = D008545, ICD-10-AM = C43) has two sub-categories, C434 (Malignant melanoma of scalp and neck) and C433 (Malignant melanoma of other and unspecified parts of the face). Horizontal lines represent a 95% confidence interval. **b** PD-causal network genes are linked to both disease MeSH terms (orange) seen in NZ PD patients and GWAS traits (green) identified in our analysis (Fig. 3a).

Comorbidity analysis for PD was conducted using patient health records within New Zealand’s integrated data infrastructure to corroborate the gene-linked co-occurring traits we have identified in this study (Fig. 1d; see ‘Methods’). The prevalence of PD in the patient population studied was 0.27% (i.e. 5556 out of the 2,051,661 individuals) of those seen by NZ’s public health system between 1 January 2016 and 31 December 2020. The comorbidity analyses identified 281 ICD-10 codes with a statistical (q-value, 0.05) relationship with PD (Supplementary Table 10). Ninety-three (33%) ICD-10 codes mapped to 100 medical conditions (MeSH terms) in the Unified Medical Language System (UMLS). Of these 100 medical conditions, 12 were also identified as being gene-linked conditions by DisGeNet (Supplementary Table 11; see ‘Methods’). Notably, the odds ratio for obesity and ovarian cysts were consistent, with them having a known reduced frequency in PD patients. However, the remaining ten conditions occurred more frequently in the PD patients (Fig. 6a).

The genes we identified within the PD-causal network were linked to GWAS traits (Fig. 3a). However, although GWAS traits can offer mechanistic insights, most are non-medical conditions. Therefore, we used gene-disease association information from the DisGeNet database to annotate the PD-causal network genes to medical conditions (MeSH terms). Of 53 genes in the PD-causal network, 33 were annotated to MeSH terms (Supplementary Table 12). The MeSH terms were then compared to those identified amongst NZ’s PD patients. Of 33 genes mapped to MeSH terms, 13 were also found to be associated with comorbid conditions in PD patients. Notably, two of these genes, *GPNMB* and *KAT8*, were identified as (i) PD-risk genes and (ii) are regulated by PD-associated loci (GPNMB - rs28624974, rs199347, KAT8 - rs14235) (Fig. 6b).

## Discussion

**Fig. 7:**
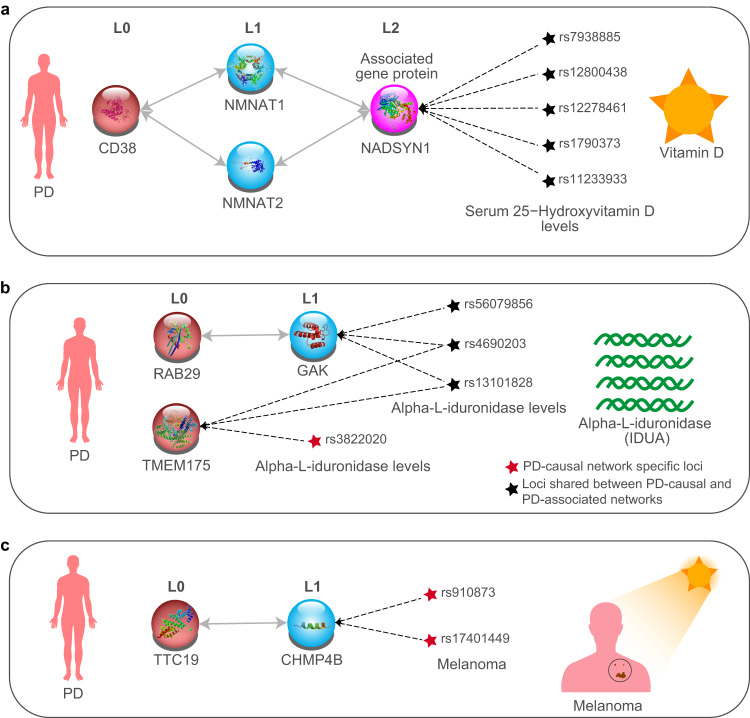
Exemplar networks that illustrate the connections between cortical changes and seemingly unrelated changes within the plasma of PD patients. **a** Nicotinamide adenine dinucleotide (NAD+) coenzyme metabolism and circulating vitamin D levels (Supplementary Table 13); **b** circulating plasma IDUA levels (Supplementary Table 14); and **c** melanoma (Supplementary Table 15).

We created and utilised a disease-agnostic BC-GRN to undertake an unbiased scaled approach that identified 19 genes whose changes in expression in the cortex were causally linked to PD. Seven of these genes were novel for their association with PD, while twelve of them (i.e. *ARHGAP27, FAM200B, TMEM175, CD38, ZSWIM7, GPNMB, STX4, KANSL1, ADORA2B, KAT8, MMRN1, PRSS36*) were previously identified by Alvarado et al.^21^, validating our approach. Notably, our study provided additional evidence for their causal role, specifically within the brain cortex. Furthermore, we identified an additional set of 300 genes that show suggestive evidence of a causal association with PD in the cortex. The chr17q21.31 inversion is particularly important as it contains three non-coding RNAs (*LINC02210, KLHL7-AS1, RP11-196G11.2*) where increases in expression were causally linked to both reductions in the risk of developing PD and non-motor symptoms. However, the extended networks on which the PD-risk genes and their associated SNPs have convergent effects also connect to established traits that co-occur with PD and hold significant allure for therapeutic development (Fig. 7). Although distinct sets of genes likely influence PD risk, symptoms, and comorbidities, additional intersecting genes and pathways suggest that the genes that occur in both the PD-causal and associated networks may contribute to increased disease risk and altered PD-related outcomes or complications. This study contributes valuable insights into the molecular mechanisms underlying PD and presents potential therapeutic targets for future investigations.

We identified *CD38* as a PD-risk gene. *CD38* encodes a multifunctional enzyme that is a key modulator of NAD metabolism and plays a central role in dictating age-related NAD decline^22^. *CD38* interacts with genes encoding proteins that are involved in the NAD biosynthetic Preiss-Handler pathway (i.e. NMNAT1/2, NADSYN1; Fig. 7a), which uses dietary nicotinic acid to synthesise NAD^23^. There is preliminary evidence that NAD supplementation (using nicotinamide riboside [NR]) increases cerebral NAD levels and clinical improvement^24^. We propose that the sceQTLs we identified as associated with the Preiss-Handler pathway may contribute to the observed heterogeneity in “NOPARK” trial participant responses to nicotinamide riboside^24^, as only certain sceQTL combinations will likely have significant impacts on the participants. Furthermore, eQTL-SNPs associated with the expression of the *NADSYN1* gene in both the PD-causal and PD-associated networks have been linked to reduced Serum 25-Hydroxyvitamin D levels by GWAS. This, in turn, may contribute to the subgroup of PD patients with significantly lower serum 25-Hydroxyvitamin D concentrations than age-matched controls^25^, and which correlated with higher UPDRS scores^26^. Therefore, reduced serum vitamin D levels may help identify individual PD patients who also have altered NAD cycling through the cortical Preiss-Handler pathway and thus would benefit from NR-based NAD replenishment therapy^24,27^. Notably, vitamin D is central to calcium homeostasis^28^, while the products of CD38 activity (i.e. cyclic ADP-ribose and nicotinic acid adenine dinucleotide phosphate) are potent mobilisers of endoplasmic and endo-lysosomal calcium stores^29,30^. Therefore, it is also tempting to speculate that these genetic variants might contribute to PD through changes that affect calcium homeostasis, and thus contribute to the selective degeneration of dopaminergic neurons^31,32^.

Loci on chromosomes 1 and 4 (e.g. *RAB29* and *TMEM175*) are widely recognised as being amongst the top risk loci for PD due to the presence of more than one independent risk signal at these loci^19^. Therefore, their identification among the PD-risk genes was not unexpected. *RAB29, GAK and TMEM175* are all connected through the lysosomal function to PD and through GWAS SNPs to alpha-L-iduronidase levels (IDUA) levels in plasma (Fig. 7b). RAB29 functionally interacts with GAK^33^ and regulatory variants of GAK are associated with elevated extracellular levels of lysosomal hydrolase IDUA (alpha-L-iduronidase) in the blood (Fig. 7b). This supports the contribution of GAK and RAB29 to PD, and potential mechanistic pathways include the mis-sorting and secretion of lysosomal hydrolases and the corresponding reduced activity of multiple hydrolases in the lysosome. Nascent soluble lysosomal hydrolases are bound by sorting receptors (e.g. mannose-6-phosphate receptor (M6PR)) in the trans-Golgi network (TGN) and sorted into clathrin-coated vesicles destined for late endosomes/lysosomes where the sorting receptors release the hydrolases in the low pH environment before undergoing retrograde transport to the TGN. Perturbation of this cyclic sorting process results in the mis-sorting*/*secretion of the hydrolase(s) and accompanying reduced hydrolase activity in lysosomes. *GAK* participates in uncoating clathrin-coated vesicles, and decreased *GAK* expression reduces the trafficking of M6PR from the TGN to the lysosome^34^. Similarly, decreased *RAB29* expression perturbs retrograde trafficking of M6PR from late endosomes/lysosomes to the TGN^35^. Elevated levels of *IDUA* in the blood are also associated with regulatory variants of *TMEM175*, a late endosomal/lysosomal protein that regulates lumenal pH and whose increased expression elevates lumenal pH^36^. Notably, deficiencies in IDUA levels have also been linked to an autosomal recessive lysosomal storage disorder (Mucopolysaccharidosis type 1)^37,38^. As such, our findings provide evidence to support the screening of patients for elevated IDUA blood levels, with the aim of identifying patients with lysosomal sorting defects and associated reductions in lysosomal hydrolase activities.

Individuals with PD have a lower risk of most cancers except for melanoma, for which they are at increased risk, while a family history of melanoma in first-degree relatives is associated with a higher risk of PD^39,40^. Despite many associations, the mechanistic underpinnings of the positive and significant genetic correlation between melanoma and PD^41^ are not fully understood. Our analysis identified the PD causative protein TTC19 as interacting with CHMP48, which was regulated by variants that are associated with melanoma (Fig. 7c). Notably, the regulatory variants associated with *CHMP48* and melanoma also regulate the expression of *ASIP* in human skin. ASIP regulates pigment synthesis and is strongly associated with the risk of melanoma and melanoma survival^42–44^. ASIP is an antagonist of MC1R signalling, which is critical for the synthesis of the UV-protective dark-coloured eumelanin, and elevated ASIP expression in the skin reduces eumelanin biosynthesis and results in the elevated production of pheomelanin, a yellow-coloured and less UV-protective isoform of melanin^45^. Increased expression of ASIP in the skin is associated with variants that increase the risk for melanoma in PD patients who possess the appropriate combination of these variants and can thus be genetically identified. Thus, our orthogonal approach has provided the first mechanistic insight into the Parkinson’s-melanoma relationship while demonstrating how inheritance of these common SNPs (MAF > 5%, for each of the sceQTLs) can co-contribute to risk for both PD and melanoma and their co-occurrence/comorbid relationship.

Despite smoking being associated with a decreased risk of PD, in individuals with PD, studies using generalised linear models have highlighted positive associations between smoking and non-motor mood-related symptoms^46,47^. However, these exploratory analyses were far from providing mechanistic insights into these associations. We observed that eQTLs targeting genes within the EGF/EGFR signalling pathway (*AP2B1, ERBB2, GAB2, PTK2, RAF1*) were also associated with smoking in the causal network. Activation of EGFR in airway epithelial cells is responsible for mucin production after inhalation of cigarette smoke in airways in vitro and in vivo^48^. Mutations in *Parkin* and *LRRK2* have both been associated with defects in EGFR internalisation, degradation and recycling (reviewed in ref. ^49^). In the brain, EGFR signalling has also been implicated in neurodegeneration through changes in the regulation of cortical astrocyte apoptosis^50^. Consistent with this, Nalls et al. identified that Parkinson’s disease has a significantly positive causal effect on smoking initiation (MR effect 0.027, SE 0.006, Bonferroni-adjusted *p* = 1.62 × 10^−5^)^19^. We contend this inter-relationship results from a genetic interaction between EGFR signalling and PD.

The position effect theory posits that a deleterious change in gene expression levels can be caused by a position change of the gene relative to its normal chromosomal environment^51^ (e.g. moving a gene can disrupt interactions with regulatory elements). Therefore, it is notable that integrating genome organisation into analyses of disease-associated eQTLs at chromosome 17q21.31 identifies a regulatory network linking variation inside and outside of the locus with changes in gene transcript levels in a phenotype-specific manner. The 17q21.31 locus exists as two haplotypes due to an ancient inversion; the H2 allele (inverted) is the ancestral organisation of this locus (H1 is the reference orientation^52^). Campoy et al.^20^ present an in-depth analysis of the 17q21.31 inversion, mapping associations with 64 traits (including many brain-related disorders) that are associated with the supergene^20^. Consistent with this, we identified regulatory interactions involving non-motor traits-associated loci within the supergene loci. We argue that the substantial contributions of the 17q21.31 supergene to PD and non-motor traits/symptoms are due to regulatory changes in spatially-constrained eQTLs that interact to link the regulation of two PD-risk genes (*LINC02210* and *KANSL1*) within the inversion boundaries. Moreover, these regulatory changes also affect the regulation of one established PD-risk gene (*ARHGAP27*) and one potentially suggestive PD-risk gene (*WNT3*) located outside the inversion boundaries (Fig. 4b, c). Therefore, we propose that position effects play a pivotal role in the inter and intra-inversion effects of eQTLs at this locus. We suggest that investigating the impacts of the relative positioning of eQTLs to the genes across the inversion boundaries would provide valuable insights into both PD risk and the associated manifestations of the supergene.

There are a number of genes that have been previously associated with PD among the 300 suggestive PD-risk genes. For example, the P14K2A protein has been shown to accumulate rapidly on damaged lysosomes as part of an endoplasmic reticulum-mediated process of lysosomal repair^53^. The identification of P14K2A as being suggestive of causality is consistent with hypotheses that the ability (or inability) to repair lysosomal damage has a significant role in PD. However, further work must be conducted to confirm this and the roles of other genes within the suggestive PD-risk genes.

This study is not without limitations, many of which derive from the reliance upon existing HiC, GWAS and PPIN data, all of which are subject to study bias. This includes a bias for participants of primarily European ancestry within GWAS studies^54^. Secondly, although incorporating brain cortex eQTL data is invaluable to our investigation, tissue sample size (*n* = 205) may be insufficient to identify all potential PD risk factors in the brain cortex. For example, despite detecting sceQTLs for *SNCA*, *LRRK2*, and *GBA* in the BC-GRN, their *p*-values did not meet the threshold (<1 × 10^−5^) required for inclusion in the MR analysis. Alternatively, it is possible that: (1) there are additional biological mechanisms that account for risk associated with these known PD genes; (2) rare high-impact mutations within these genes are responsible; or (3) these genes’ association with PD are not via the cortex. Thirdly, our analysis does not include sceQTL target genes that do not have curated interactions in STRING. Fourthly, the gene regulatory and protein interaction networks we generated were not dynamic, representing only a snapshot of possible interactions, and are thus subject to change. Finally, our analyses combine information sources that do not originate from the same biological samples (i.e. eQTL data from GTEx^55^, Hi-C datasets, GWAS, and interaction data). Notwithstanding these limitations, our approach improves our ability to identify the direct contribution(s) of PD-risk genes and associated traits to PD.

In summary, we used Mendelian Randomisation to identify 19 genes whose changes in expression in the cortex were causally linked to PD, and further integration of gene and protein interaction datasets enabled the identification of the wider networks associated with these genes. However, our results have significant mechanistic implications beyond identifying these PD-risk genes, such as potential links between cortical non-motor function and changes in the plasma (e.g. IDUA and vitamin D). The altered protein interaction networks and eQTLs that are associated with these changes provide valuable resources and potential biomarkers for designing and stratifying clinical trials that aim to target therapies for non-motor PD symptoms. Initially, these findings may be used to retrospectively stratify already-completed clinical trials, with the potential to rescue previously failed trials by sub-selecting individuals who possess altered regulatory elements for the genes within the targeted pathway (including modifiers). Beyond this, and moving forward, incorporating this knowledge as an a priori during trial design will be a critical step towards reducing initial failure rates and taking a more precision medicine approach towards treating PD.

## Methods

### Building the brain cortex eQTL gene regulatory network (BC-GRN)

All genetic variants called in the whole genome sequencing of the GTEx project^55^ (dbGaP accession phs000424.v8.p2, project #22937) were downloaded (10 January 2022) and run through the CoDeS3D algorithm^56^ to identify spatially constrained expression quantitative loci (i.e. sceQTL)-gene associations. Briefly, the CoDeS3D algorithm identified HindIII digested restriction fragments harbouring genetic variants (i.e. SNPs) from Hi-C libraries of dorsolateral prefrontal cortex cells^57^. Gene-harbouring fragments that were captured as physically interacting with the variant-harbouring fragments were identified. SNP-gene pairs with >1 interaction in >1 Hi-C biological replicate were then tested for eQTL associations using tensorQTL^58^. SNP-gene pairs that pass a chromosome-based Benjamini–Hochberg false discovery rate (FDR < 0.05) correction were deemed significant sceQTL associations and formed the brain cortex gene regulatory network (BC-GRN). The BC-GRN comprises sceQTL associations categorised as cis (where linear DNA distance between sceQTL and gene is <1 Mb), trans-intra-chromosomal (where linear DNA distance between sceQTL and gene is >1 Mb on the same chromosome), and trans-inter-chromosomal (where sceQTL and gene is on different chromosome) (Fig. 1a).

### Identification of PD causal genes in BC-GRN using Mendelian Randomisation (MR)

To estimate the causality explained by changes in gene expression (i.e. exposure) on the outcome (i.e. disease) using MR, each genetic variant included in the MR analysis must satisfy three basic assumptions: (i) it is associated with the exposure (i.e. relevance assumption), (ii) it is not associated with any confounder of the exposure-outcome association (i.e. independence or exchangeability assumption), and (iii) it is only associated with the outcome through the exposure (i.e. exclusion restriction assumption). A genetic variant satisfying these assumptions is designated an instrumental variable(s) (IVs). Here, we used sceQTLs within the BC-GRN as IVs to estimate the causality explained by their target genes within the BC-GRN on PD (Fig. 1b). Given that the number of brain cortex samples used for the eQTL analysis in GTEx^55^ is small (*n* = 205) and only genetic variant – gene association confirmed by both chromatin interaction and eQTL analysis were included in the analysis, using a stringent association threshold (*p* < 5 × 10^−8^) would eliminate most of the sceQTLs and genes. Therefore, to select valid sceQTLs from the BC-GRN, we used a less stringent *p*-value threshold 1 × 10^−5^ as it is shown that eQTLs with association *p*-value < 1 × 10^−5^ would greatly minimize the weak instrument bias^13,59,60^. The sceQTLs that had passed this association threshold were chosen as IVs. These IVs and their respective target genes were included in the exposure dataset. Furthermore, to obtain independent IVs for each gene (exposure), we performed LD clumping using the *ld_clump()* function in the *TwoSampleMR* package (v0.5.6)^61^. European superpopulation (EUR) from the 1000 genome project (Phase III) was used as a reference panel for clumping. In a 10 Mb window, two or more SNPs were considered independent only if they were not linked (LD; r^2^ < 0.001).

The largest PD GWAS reported by Nalls et al.^19^ was used as outcome data for MR analysis. The summary statistics were accessed from the IEU OpenGWAS portal (GWAS ID: ieu-b-7) using *the extract_outcome_data()* function in the *TwoSampleMR* package (v0.5.6)^61^. Only GWAS summary statistics of publicly available datasets (33,674 cases and 449,056 control 17,891,936 SNPs) were included in this study. When an IV was absent in the outcome GWAS, we replaced it with their proxy SNPs (r^2^ > 0.9). When a proxy SNP was also unavailable, we excluded the IV from the analysis.

Sensitivity analysis was performed for exposures with more than one IV. The presence of heterogeneity means violation of IV assumptions for which horizontal pleiotropy is likely the main cause. We performed a heterogeneity test using the *mr_heterogeneity()* (Cochrane Q method) function in the *TwoSampleMR* package^61^ and removed exposures with IVs having significant heterogeneity (Q_pval < 0.05). Further, we evaluated the horizontal pleiotropic effect using the MR-Egger intercept term, which was performed using *mr_pleiotropy_test()*. It is considered that directional pleiotropy is present when the MR-intercept term differs from the null (*p* < 0.05).

We harmonised the exposure and outcome datasets to ensure the estimated causal effect is attributed to the same allele in both datasets. Harmonisation was done using *the harmonise_data()* function in the *TwosampleMR* package^61^. The Wald ratio (also known as the ratio of coefficients) method is the most straightforward way of estimating the causal effect of the exposure on the outcome, so it was used for calculating the causal effect of exposures with only one IV. In short, the Wald ratio causal estimate was obtained by dividing the coefficient of the IV-outcome association by the coefficient of the IV-exposure association. The inverse variance weighted (IVW) method was used for exposure with more than one IV. The MR analysis was performed using the *mr_singlesnp()* function in the *TwosampleMR* package^61^. The MR analysis results were corrected using the Bonferroni multiple testing correction procedure. The exposures with *p*-values lower than the Bonferroni multiple testing correction threshold (*p* < 0.05/number of exposures) were considered genes causally linked to PD (i.e. “PD-risk” genes). We used an extremely conservative correction threshold to prevent the inclusion of false positive findings. The association between an exposure and outcome is considered suggestive if adjusted *p* > 0.05/number of exposures and <0.05.

### Identifying traits with shared molecular interactions with PD

We undertook a de novo approach to identify traits with shared molecular interactions with PD. We used the multimorbid3D algorithm^17,18^ to integrate BC-GRN, protein-protein interactions (PPIs) and SNP-trait associations from GWAS Catalog^62^ to identify traits that are linked to PD-risk genes and PD-associated GWAS loci. Firstly, the multimorbid3D pipeline used the BC-GRN to identify the significant sceQTLs that are associated with the PD-risk genes in the cortex. The PD-risk genes with their regulatory loci constitute ‘level0 (L0)’ of the “PD-causal network”. Similarly, we identified genes targeted by the 90 PD-associated SNPs (from Nalls et al.^19^) and SNPs that are strongly linked to them with linkage disequilibrium (LD) (r^2^ ≥ 0.8, within ± 5000 bp window, using 1000 genomes phase III EUR super-population). The resulting gene set and the associated sceQTLs formed the ‘level0 (L0)’ of the “PD-associated network” (Fig. 1c).

Since the proteins encoded by genes do not function independently, identifying all the protein-encoding genes interacting with the L0 genes (using PPI data) could help identify additional genes relevant to PD. We therefore interrogated the STRING database^63^ (accessed 30/03/2023) to identify genes/proteins that interact with the L0 genes/proteins. The genes encoding the interacting proteins, together with their associated sceQTLs, form the next level (or L1) of the PD-causal network and PD-associated network. This process was repeated to obtain five levels of the protein interaction network (Fig. 1c).

We tested for traits in the GWAS Catalog (accessed 30/03/2023) that are enriched for sceQTLs (and SNPs in strong linkage disequilibrium (R^2^ > = 0.8) with these sceQTLs) at each level (Fig. 1c). To do this, we calculated the hypergeometric survival function for each trait; thus,

1 $${sf}\left({Trait},{Level}\right)=1-\mathop{\sum }\limits_{i=0}^{n}\frac{{{{n}\choose{{x}_{i}}}}{{{M-n}\choose{N-{x}_{i}}}}}{{{M}\choose{N}}}$$

where *M* is the total number of unique SNPs in the GWAS Catalog^62^, *n* is the number of SNPs associated with a given trait, *N* is the number of BC-GRN sceQTLs identified at the given level (plus SNPs in LD as described above), *x* is the number of sceQTLs (plus SNPs in LD) associated with the given trait at the given level. Bonferroni correction (FDR < 0.05) was subsequently applied to obtain significantly enriched traits at each level.

Monte Carlo simulations (*n* = 1000) were performed to identify traits that are enriched within the PD-causal and PD-associated gene networks that are not accounted for by chance alone. Each simulation was started with a random set of genes of the same size as the L0 gene set (for each PD-causal and PD-associated network), selected from the BC-GRN. The simulations were then run through the multimorbid3D pipeline.

### Identification of biological pathways enriched for genes within the PD-causal and associated networks

We aimed to identify biological pathways in which genes that form the PD-causal and PD-associated gene networks are over-represented. *Gprofiler2* was used to perform the enrichment analysis for pathways in KEGG, REACTOME and WikiPathways with all genes in BC-GRN as background set. All enrichment *p*-values were corrected for multiple testing using the *‘fdr’* correction method implemented in the *gprofiler2* package. Only those pathways that passed the corrected *p*-value threshold of <0.05 are reported in this study.

### Identification of diseases associated with PD-causal network genes

We aimed to identify the disease conditions associated with the genes forming the PD-causal network. Since the GWAS catalog contains ‘traits’ that are not clinically recognised or diseases (e.g. household income, white matter microstructure), we retrieved “curated” gene-disease associations from DisGeNET^64^ (v7.0) using *gene2disease* function (*disgenet2r* package^64^) for the genes. Disease annotations were mapped to their corresponding Medical Subject Heading (MeSH) identifiers using the mapping information provided on the DisGeNET portal (https://www.disgenet.org/downloads).

### Comorbidity analysis

The New Zealand (NZ) Integrated Data Infrastructure was interrogated for diagnostic records from patients who were seen by NZ’s public health system between 1 January 2016 and 31 December 2020. Records of patients (<100 years of age) were used in this analysis (*N* = 2,051,661). Statistics New Zealand (project number MAA2020-63) reviewed and approved the use of the Integrated Data Infrastructure within Stats NZ Data Lab. The comoRbidity^65^ R algorithm was used to identify conditions that were co-occurring with PD (ICD-10-AM code, G20) in the NZ patients. Measures of comorbidity (i.e. relative risk, odds ratio, and comorbidity score) were calculated as defined below:

2 $${{{\rm{Relative}}}\,{{\rm{risk}}}}_{{AB}}=\frac{{C}_{{AB}}N}{{P}_{A}{P}_{B}}$$

3 $${{{\rm{Odds}}}\,{{\rm{ratio}}}}_{{AB}}=\frac{{C}_{{AB}}H}{{C}_{A}{C}_{B}}$$

4 $${{\rm{Comorbidity}}}\,{{\rm{score}}}={\log }_{2}\left(\frac{{{\rm{observed}}}\,{C}_{{AB}}+1}{{{\rm{expected}}}\,{C}_{{AB}}+1}\right),\,{{\rm{expected}}}\,{C}_{{AB}}=\frac{{P}_{A}{P}_{B}}{N}$$

where *A* is PD and *B* is the disease condition being tested, *C*_*AB*_ is the number of patients diagnosed with PD and disease *B*, *N* is the total number of patients in the population, *P*_*A*_ and *P*_*B*_ are the prevalence of PD and disease *B*, respectively, *H* is the number of patients without PD and disease *B*, *C*_*A*_ is the number of patients diagnosed with PD, and *C*_*B*_ is the number of patients diagnosed with disease *B*. Conditions with <6 patients were excluded from the analysis. Conditions with a 95% confidence interval of odd ratios overlapping zero were also excluded from the analysis. The ICD-10-AM codes were converted to MeSH terms using the Unified Medical Language System (2022AA release) API from the National Library of Medicine.

### Reporting summary

Further information on research design is available in the Nature Research Reporting Summary linked to this article.

### Supplementary information

Supplemental material

Reporting summary

## Data Availability

All data analysed or produced throughout this research is detailed in the article or Supplementary Material. Additionally, publicly accessible datasets obtained and utilised in our study were Genotype-Tissue Expression (GTEx) Portal (https://www.gtexportal.org/home/), STRING (https://string-db.org/), DisGeNet (https://www.disgenet.org/home/).

## Code availability

Custom scripts used for data analyses and visualisation are available at: https://github.com/Genome3d/Parkinsons_disease_genetics.git. Code for bioinformatics software or algorithm is available at: CoDeS3D (https://github.com/Genome3d/codes3d.git), Multimorbid3D (https://github.com/Genome3d/multimorbid3D.git), STRING API (https://github.com/gpp-rnd/stringdb), TwoSampleMR (https://github.com/MRCIEU/TwoSampleMR), comoRbidty: https://bitbucket.org/ibi_group/comorbidity, and UMLS API: https://github.com/HHS/uts-rest-api.
