# Supplementary material for: Discovering genetic mechanisms underlying the co-occurrence of Parkinson’s disease and non-motor traits
Source: NPJ Parkinsons Dis. 2024 Jan 23;10:27. doi: 10.1038/s41531-024-00638-w (PMC10805842; doi:10.1038/s41531-024-00638-w)
Supplement: Supplementary file 1 — Supplemental material [file 41531_2024_638_MOESM1_ESM.pdf]

Discovering genetic mechanisms underlying the co-occurrence of Parkinson's disease and non-motor traits

Sreemol Gokuladhas[1], Tayaza Fadason[1,2], Sophie Farrow[1,2], Antony Cooper[3,4], Justin M. O'Sullivan[1,2,4,5,6*]

1. The Liggins Institute, University of Auckland, Auckland 1023, New Zealand.
2. Maurice Wilkins Centre for Molecular Biodiscovery, Auckland 1010, New Zealand.
3. St Vincent's Clinical School, UNSW Sydney, Sydney, NSW, Australia.
4. Australian Parkinson's Mission, Garvan Institute of Medical Research, Sydney, New South Wales, Australia.
5. MRC Lifecourse Epidemiology Unit, University of Southampton, United Kingdom.
6. Singapore Institute for Clinical Sciences, Agency for Science, Technology and Research (A*STAR), Singapore, Singapore.
*Corresponding author: justin.osullivan@auckland.ac.nz

Supplementary Figures

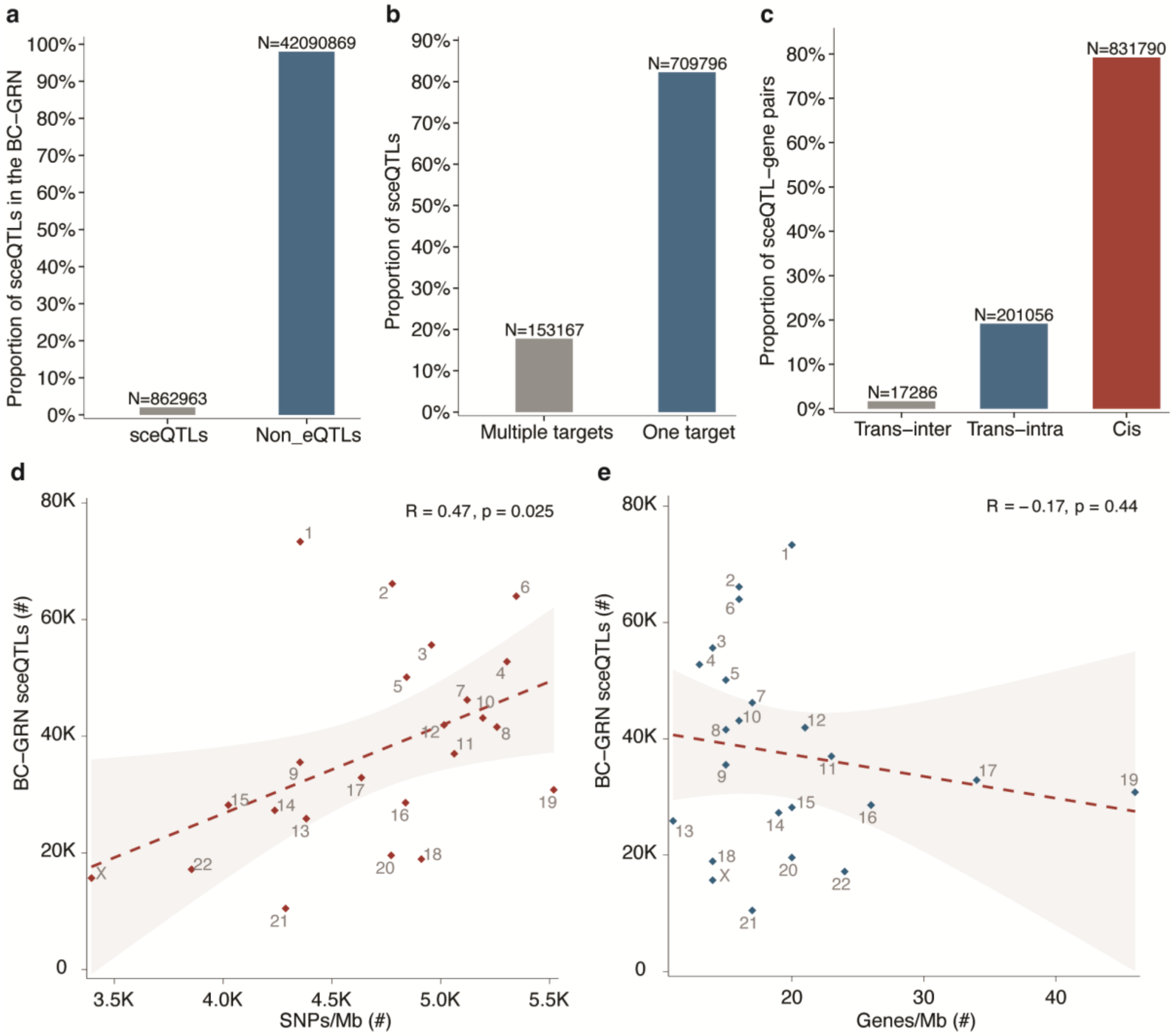

Supplementary Figure 1. General features of BC-GRN.

a Of 42,953,832 SNPs with valid reference rsID genotyped in GTEx (V8), only 8,62,963 (2%) SNPs were found to be associated with the expression of genes via physical interaction (captured using Hi-C data) in adult brain cortex. **b** The large proportion of identified sceQTLs (82%) in the BC-GRN had only one target gene. **c** A vast majority of regulatory interactions were identified between sceQTLs and genes that are located within 1Mb (*cis*) distance. In addition to that, a considerable number of *trans* (sceQTLs and genes located on

different chromosomes (trans-inter), or within the same chromosomes with >1Mb apart) sceQTL-gene regulatory interactions were also identified. **d** There is a positive correlation between the number of sceQTLs identified within BC-GRN and the total number of common SNPs on each chromosome. **e** However, negative correlation is observed between the number of sceQTLs and the total number of genes on each chromosome. *sceqtls – spatially constrained eQTLs

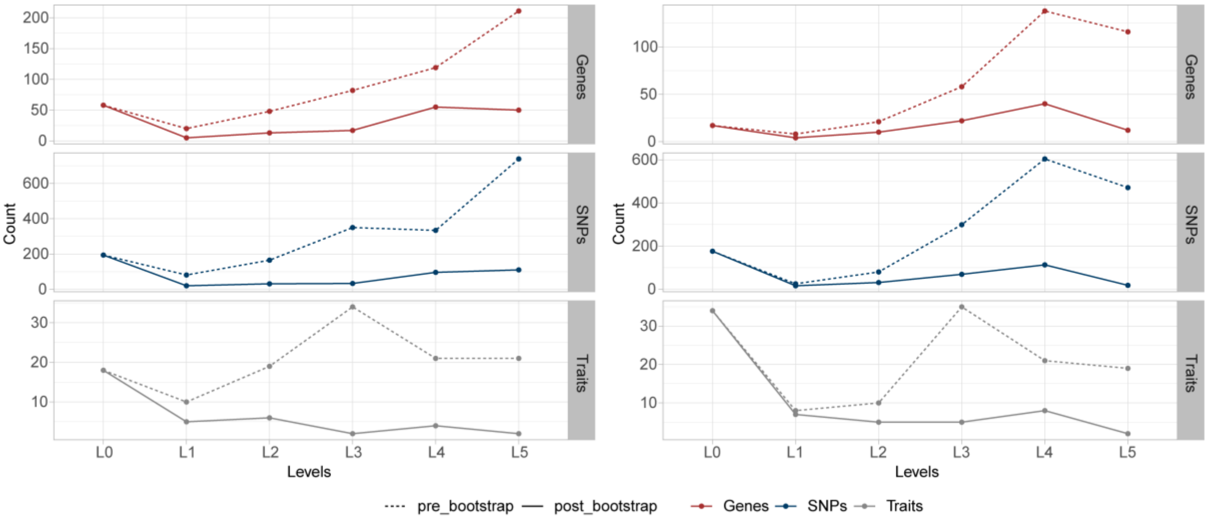

Supplementary Figure 2. Distribution of genes, SNPs and Traits across expanded PD-associated and PD-causal networks.

This figure shows the number of genes, their regulatory loci and traits associated with those loci across L0 to L5 of the PD-associated network (left) and PD-causal network (right) before and after bootstrapping was performed. Bootstrapping was performed to minimise the risk of including false-positive trait associations.

Supplementary Tables

Column header definitions

SNP	single nucleotide polymorphism. It is associated with a GWAS trait and may be a sceQTL in the brain cortex or in Linkage disequilibrium with a sceQTL
b	beta (or effect size) from MR analysis
SE	Standard error
p	p-value from MR analysis
lo-CI	lower confidence interval of b
up-CI	upper confidence interval of b
OR/or	Odds ratio
OR_lci95	Odds ratio lower 95% confidence interval
OR_uci95	Odds ratio upper 95% confidence interval
Target gene	Target gene of the SNP
PPI level	Protein-protein interaction level
Bootstrap_pval	P-value obtained from bootstrap analysis. Pval <0.05 means less likely to have found by chance
Bonferroni_adj_pval	Bonferroni adjusted hypergeometric trait enrichment p-values
eQTL beta	effect size of an eQTL (NA = GWAS SNP is not an eQTL but in linkage disequilibrium with an eQTL SNP in the BC-GRN)
Disease code	ICD-10-AM code of disease that co-morbid with PD
Major class	Major class of the disease
descShort	Short description of the disease
trait	GWAS trait associated with the snp

Supplementary Table 1. Mendelian Randomization analysis identified PD-risk genes at suggestive level significance (1.28e-05 > p < 0.05).

Exposure (gene)	Outcome	SNP	b	SE	p	lo_CI	up_CI	OR	OR_lci95	OR_uci95
FAM47E	PD | ieu-b-7	rs1876540	-0.1041	0.0239	1.31E-05	-0.1509	-0.0573	0.9011	0.8599	0.9443
ZNF391	PD | ieu-b-7	rs4713090	0.1920	0.0466	3.73E-05	0.1008	0.2833	1.2117	1.1060	1.3275
CTSB	PD | ieu-b-7	rs6990033	-0.4259	0.1042	4.39E-05	-0.6302	-0.2216	0.6532	0.5325	0.8012
CENPV	PD | ieu-b-7	rs3112520	0.0982	0.0251	8.96E-05	0.0491	0.1473	1.1032	1.0503	1.1587
STBD1	PD | ieu-b-7	rs3796491	0.2237	0.0577	1.07E-04	0.1105	0.3368	1.2506	1.1168	1.4005
PPP1R21	PD | ieu-b-7	rs76875602	-0.1988	0.0528	1.68E-04	-0.3023	-0.0953	0.8197	0.7391	0.9091
AMIGO1	PD | ieu-b-7	rs1149170	-0.3865	0.1030	1.74E-04	-0.5884	-0.1847	0.6794	0.5552	0.8314
AC074117.10	PD | ieu-b-7	rs13472	0.2263	0.0622	2.72E-04	0.1045	0.3481	1.2539	1.1101	1.4164
ADCY9	PD | ieu-b-7	rs12920521	0.3442	0.0983	4.61E-04	0.1516	0.5368	1.4108	1.1636	1.7104
ZC3H7B	PD | ieu-b-7	rs11090045	-0.4252	0.1224	5.11E-04	-0.6650	-0.1853	0.6537	0.5143	0.8308
MCCC1	PD | ieu-b-7	rs11714270	-0.1646	0.0474	5.16E-04	-0.2576	-0.0717	0.8482	0.7729	0.9308
TAL1	PD | ieu-b-7	rs2008713	0.2760	0.0809	6.45E-04	0.1175	0.4346	1.3179	1.1246	1.5444
DRAM1	PD | ieu-b-7	rs12230626	-0.2646	0.0781	6.99E-04	-0.4176	-0.1116	0.7675	0.6586	0.8944
EPG5	PD | ieu-b-7	rs967137	0.0917	0.0273	7.66E-04	0.0383	0.1452	1.0961	1.0390	1.1562
PLCXD2	PD | ieu-b-7	rs4527400	-0.3423	0.1018	7.69E-04	-0.5418	-0.1429	0.7101	0.5817	0.8669
MEI1	PD | ieu-b-7	rs4822027	-0.1427	0.0431	9.30E-04	-0.2272	-0.0582	0.8670	0.7968	0.9434
SH3GL2	PD | ieu-b-7	rs10810821	0.4745	0.1439	9.74E-04	0.1925	0.7565	1.6072	1.2123	2.1307
BIN3	PD | ieu-b-7	rs11136093	0.1474	0.0448	9.94E-04	0.0596	0.2351	1.1588	1.0614	1.2651
DEGS2	PD | ieu-b-7	rs4905934	-0.1996	0.0611	0.001089337	-0.3193	-0.0798	0.8191	0.7266	0.9233
C20orf194	PD | ieu-b-7	rs6084342	0.1620	0.0498	0.00115405	0.0643	0.2597	1.1759	1.0664	1.2965
FAHD1	PD | ieu-b-7	rs11248893	-0.0848	0.0262	0.001211955	-0.1361	-0.0334	0.9187	0.8727	0.9671
ENPP5	PD | ieu-b-7	rs2206965	-0.1039	0.0322	0.001254498	-0.1670	-0.0408	0.9013	0.8462	0.9600
NOP14-AS1	PD | ieu-b-7	rs16843603	0.2670	0.0831	0.001316148	0.1041	0.4298	1.3060	1.1097	1.5370

TNNI3K	PD | ieu-b-7	rs34018059	-0.1782	0.0560	0.001444939	-0.2879	-0.0686	0.8367	0.7498	0.9337
TYW5	PD | ieu-b-7	rs281787	-0.1576	0.0496	0.001480027	-0.2548	-0.0604	0.8542	0.7750	0.9414
ITGA8	PD | ieu-b-7	rs10796307	-0.1548	0.0490	0.001579997	-0.2508	-0.0588	0.8566	0.7782	0.9429
SCP2	PD | ieu-b-7	rs495985	-0.3442	0.1098	0.00172833	-0.5595	-0.1289	0.7088	0.5715	0.8791
CTD-3247F14.2	PD | ieu-b-7	rs1059592	0.1680	0.0536	0.001739581	0.0628	0.2731	1.1829	1.0648	1.3140
PLPP5	PD | ieu-b-7	rs56322953	0.2353	0.0753	0.00178526	0.0877	0.3829	1.2653	1.0916	1.4666
ACTR10	PD | ieu-b-7	rs79224182	0.3534	0.1137	0.001872265	0.1307	0.5762	1.4240	1.1396	1.7793
XXbac-BPG154L12.5	PD | ieu-b-7	rs2273019	0.1008	0.0329	0.002174324	0.0363	0.1652	1.1060	1.0370	1.1796
RCBTB1	PD | ieu-b-7	rs7336027	-0.1653	0.0544	0.002358442	-0.2719	-0.0588	0.8476	0.7619	0.9429
SLC25A24	PD | ieu-b-7	rs538717	-0.1204	0.0396	0.002365781	-0.1980	-0.0428	0.8866	0.8204	0.9581
AREL1	PD | ieu-b-7	rs11844127	-0.4726	0.1556	0.002385494	-0.7775	-0.1676	0.6234	0.4596	0.8457
PCGF3	PD | ieu-b-7	rs34434107	-0.1489	0.0496	0.002699796	-0.2461	-0.0516	0.8617	0.7818	0.9497
P2RX7	PD | ieu-b-7	rs208299	0.1665	0.0558	0.002843887	0.0572	0.2759	1.1812	1.0588	1.3178
PDLIM2	PD | ieu-b-7	rs7846476	-0.1542	0.0517	0.002858504	-0.2556	-0.0529	0.8571	0.7745	0.9485
RP11-1348G14.4	PD | ieu-b-7	rs62036613	0.1056	0.0355	0.002908252	0.0361	0.1750	1.1113	1.0367	1.1913
LARP1B	PD | ieu-b-7	rs10001048	0.2637	0.0892	0.003121065	0.0888	0.4386	1.3018	1.0929	1.5505
RNASET2	PD | ieu-b-7	rs9356551	0.2294	0.0781	0.003315729	0.0763	0.3825	1.2578	1.0793	1.4659
DNAH10OS	PD | ieu-b-7	rs9863	0.1407	0.0481	0.00342286	0.0465	0.2349	1.1511	1.0476	1.2648
ZYG11B	PD | ieu-b-7	rs545719	0.3104	0.1063	0.003495212	0.1021	0.5187	1.3640	1.1075	1.6799
CTD-2378E12.1	PD | ieu-b-7	rs2619691	-0.1488	0.0515	0.003852418	-0.2498	-0.0479	0.8617	0.7790	0.9532
SCFD2	PD | ieu-b-7	rs4693003	-0.2017	0.0699	0.003930069	-0.3388	-0.0646	0.8173	0.7126	0.9374
RP11-707A18.1	PD | ieu-b-7	rs2033944	0.1466	0.0512	0.004233974	0.0461	0.2470	1.1579	1.0472	1.2802
ANKMY1	PD | ieu-b-7	rs3821348	-0.0992	0.0348	0.004307738	-0.1673	-0.0311	0.9055	0.8459	0.9694
PI4K2A	PD | ieu-b-7	rs7901060	0.2429	0.0852	0.004375897	0.0758	0.4100	1.2750	1.0788	1.5068
GRK4	PD | ieu-b-7	rs28619042	-0.0794	0.0279	0.004431193	-0.1341	-0.0247	0.9236	0.8745	0.9756

RP1-8B1.4	PD	ieu-b-7	rs4496799	-0.0627	0.0221	0.004527092	-0.1060	-0.0194	0.9392	0.8995	0.9808
LNP1	PD	ieu-b-7	rs277646	-0.1849	0.0654	0.004720219	-0.3131	-0.0566	0.8312	0.7312	0.9449
RP11-313J2.1	PD	ieu-b-7	rs2466466	0.0634	0.0225	0.004788007	0.0194	0.1075	1.0655	1.0195	1.1135
AC007966.1	PD	ieu-b-7	rs6758929	-0.0475	0.0169	0.004842811	-0.0805	-0.0144	0.9536	0.9226	0.9857
DDHD2	PD	ieu-b-7	rs6992943	0.2591	0.0922	0.00495415	0.0784	0.4398	1.2958	1.0815	1.5524
AVPI1	PD	ieu-b-7	rs12358715	-0.2284	0.0813	0.0049649	-0.3878	-0.0691	0.7958	0.6786	0.9333
MORN4	PD	ieu-b-7	rs12358715	0.1100	0.0392	0.0049649	0.0333	0.1868	1.1163	1.0338	1.2053
PROM2	PD	ieu-b-7	rs6719098	0.1268	0.0453	0.00509453	0.0381	0.2155	1.1351	1.0388	1.2404
DALRD3	PD	ieu-b-7	rs6768433	0.1850	0.0663	0.005236153	0.0551	0.3149	1.2033	1.0567	1.3701
G3BP2	PD	ieu-b-7	rs13142804	-0.4551	0.1630	0.005243734	-0.7745	-0.1356	0.6344	0.4609	0.8732
TXNDC15	PD	ieu-b-7	rs79082746	-0.1119	0.0401	0.005288293	-0.1906	-0.0333	0.8941	0.8265	0.9673
CAMLG	PD	ieu-b-7	rs145506998	-0.0581	0.0208	0.00535366	-0.0989	-0.0172	0.9436	0.9058	0.9829
C4orf26	PD	ieu-b-7	rs2061742	0.1499	0.0539	0.005403921	0.0443	0.2555	1.1617	1.0453	1.2911
ZNF660	PD	ieu-b-7	rs939649	-0.0788	0.0283	0.005412892	-0.1344	-0.0233	0.9242	0.8743	0.9770
HPR	PD	ieu-b-7	rs3794695	-0.0557	0.0201	0.005581092	-0.0951	-0.0163	0.9458	0.9093	0.9838
RP4-605O3.4	PD	ieu-b-7	rs11831413	0.0800	0.0289	0.005649812	0.0233	0.1366	1.0833	1.0236	1.1464
WDR6	PD	ieu-b-7	rs7627404	-0.1614	0.0586	0.005908636	-0.2763	-0.0465	0.8510	0.7586	0.9546
PLS1	PD	ieu-b-7	rs7641914	0.2241	0.0816	0.006037041	0.0641	0.3840	1.2511	1.0662	1.4681
TSPAN15	PD	ieu-b-7	rs1227967	-0.2981	0.1087	0.00610512	-0.5111	-0.0850	0.7422	0.5998	0.9185
NCKIPSD	PD	ieu-b-7	rs6792510	-0.1180	0.0434	0.006496851	-0.2030	-0.0330	0.8887	0.8163	0.9675
DISP3	PD	ieu-b-7	rs2594302	-0.1635	0.0601	0.006525246	-0.2813	-0.0457	0.8492	0.7548	0.9553
IFT122	PD	ieu-b-7	rs4429625	0.1017	0.0374	0.006531596	0.0284	0.1750	1.1071	1.0288	1.1913
PDCD1LG2	PD	ieu-b-7	rs7860373	-0.1426	0.0526	0.006713248	-0.2457	-0.0395	0.8671	0.7822	0.9613
SULT2B1	PD	ieu-b-7	rs2665580	0.1378	0.0508	0.006715384	0.0382	0.2374	1.1477	1.0389	1.2680
RP11-1084I9.1	PD	ieu-b-7	rs4906840	-0.1315	0.0488	0.007001473	-0.2271	-0.0359	0.8768	0.7969	0.9647
ITCH	PD	ieu-b-7	rs6059909	0.3073	0.1147	0.007407071	0.0824	0.5322	1.3597	1.0859	1.7026
PSMD2	PD	ieu-b-7	rs6845	-0.3460	0.1305	0.008003225	-0.6018	-0.0903	0.7075	0.5478	0.9137

TOR1A	PD | ieu-b-7	rs7025992	-0.2506	0.0946	0.008055226	-0.4360	-0.0652	0.7783	0.6466	0.9368
PDHB	PD | ieu-b-7	rs72624863	-0.1080	0.0409	0.008334963	-0.1882	-0.0278	0.8977	0.8285	0.9726
GPR161	PD | ieu-b-7	rs7522216	0.1280	0.0485	0.00836402	0.0329	0.2232	1.1366	1.0334	1.2500
NEIL2	PD | ieu-b-7	rs804286	-0.1484	0.0563	0.008368823	-0.2588	-0.0381	0.8621	0.7720	0.9626
PANK2	PD | ieu-b-7	rs34562926	0.2639	0.1001	0.008379987	0.0677	0.4601	1.3020	1.0701	1.5842
AEBP1	PD | ieu-b-7	rs113784062	0.1574	0.0597	0.008425689	0.0403	0.2745	1.1705	1.0411	1.3159
AADAT	PD | ieu-b-7	rs4692790	-0.1714	0.0651	0.008436605	-0.2990	-0.0439	0.8425	0.7416	0.9571
KTN1	PD | ieu-b-7	rs28703642	0.2396	0.0912	0.008570869	0.0610	0.4183	1.2708	1.0629	1.5193
MCM3AP	PD | ieu-b-7	rs62226486	-0.2324	0.0885	0.008594464	-0.4058	-0.0591	0.7926	0.6664	0.9426
CD247	PD | ieu-b-7	rs7513428	0.1466	0.0560	0.008829406	0.0369	0.2562	1.1578	1.0375	1.2921
VCL	PD | ieu-b-7	rs767809	0.1753	0.0670	0.0088793	0.0440	0.3067	1.1917	1.0450	1.3589
POLR3H	PD | ieu-b-7	rs9611611	-0.1069	0.0409	0.008958433	-0.1871	-0.0267	0.8986	0.8294	0.9736
EFCAB1	PD | ieu-b-7	rs1871889	-0.0689	0.0264	0.009159025	-0.1208	-0.0171	0.9334	0.8863	0.9831
ICK	PD | ieu-b-7	rs160631	-0.2657	0.1025	0.009516481	-0.4665	-0.0649	0.7667	0.6272	0.9372
TCTEX1D1	PD | ieu-b-7	rs6697073	0.0779	0.0301	0.009640128	0.0189	0.1369	1.0810	1.0191	1.1467
ATG14	PD | ieu-b-7	rs6573020	-0.1222	0.0473	0.009731483	-0.2149	-0.0296	0.8850	0.8067	0.9709
GBAS	PD | ieu-b-7	rs11771788	-0.3244	0.1262	0.010147086	-0.5716	-0.0771	0.7230	0.5646	0.9258
SMIM2-AS1	PD | ieu-b-7	rs4942281	-0.0751	0.0294	0.010601844	-0.1327	-0.0175	0.9277	0.8758	0.9827
CCDC162P	PD | ieu-b-7	rs6925339	0.0726	0.0285	0.01076412	0.0168	0.1283	1.0753	1.0169	1.1369
CCNT2	PD | ieu-b-7	rs12617286	-0.1870	0.0733	0.010787598	-0.3307	-0.0432	0.8295	0.7184	0.9577
GALNT6	PD | ieu-b-7	rs2241545	-0.1253	0.0492	0.010862691	-0.2217	-0.0289	0.8822	0.8012	0.9715
TDRD3	PD | ieu-b-7	rs4886224	-0.2726	0.1078	0.011417822	-0.4838	-0.0614	0.7614	0.6164	0.9405
RAP1GAP2	PD | ieu-b-7	rs57282053	-0.6517	0.2578	0.011470664	-1.1569	-0.1464	0.5212	0.3144	0.8638
YIPF7	PD | ieu-b-7	rs10938361	-0.0982	0.0390	0.011784969	-0.1747	-0.0218	0.9065	0.8398	0.9785
GPA33	PD | ieu-b-7	rs6660754	-0.0868	0.0345	0.011931289	-0.1544	-0.0191	0.9169	0.8569	0.9811
TSPYL5	PD | ieu-b-7	rs112664809	0.3095	0.1233	0.012099636	0.0677	0.5512	1.3627	1.0701	1.7353
GOLPH3L	PD | ieu-b-7	rs12409208	-0.1997	0.0796	0.012122046	-0.3558	-0.0437	0.8189	0.7006	0.9573

GNPDA2	PD | ieu-b-7	rs12639722	-0.0762	0.0305	0.012419331	-0.1359	-0.0164	0.9267	0.8730	0.9837
GUF1	PD | ieu-b-7	rs12639722	0.0687	0.0275	0.012419331	0.0148	0.1225	1.0711	1.0149	1.1303
RP11-315D16.4	PD | ieu-b-7	rs55941639	-0.0555	0.0222	0.012419331	-0.0990	-0.0120	0.9460	0.9058	0.9881
TRPM2	PD | ieu-b-7	rs7278527	0.1962	0.0787	0.012663761	0.0420	0.3505	1.2168	1.0429	1.4198
RPS17	PD | ieu-b-7	rs1259180	-0.2106	0.0846	0.012832939	-0.3765	-0.0447	0.8101	0.6863	0.9563
AP001347.6	PD | ieu-b-7	rs12627691	0.0803	0.0324	0.013218318	0.0168	0.1439	1.0837	1.0169	1.1547
C1orf27	PD | ieu-b-7	rs10911849	-0.2017	0.0814	0.01323907	-0.3613	-0.0421	0.8173	0.6968	0.9588
SFXN4	PD | ieu-b-7	rs10749291	0.0528	0.0214	0.013479158	0.0109	0.0947	1.0542	1.0110	1.0993
RP11-104N10.2	PD | ieu-b-7	rs55952217	0.0465	0.0188	0.013489104	0.0096	0.0835	1.0476	1.0097	1.0870
SMIM22	PD | ieu-b-7	rs960006	-0.0931	0.0378	0.013728663	-0.1672	-0.0191	0.9111	0.8461	0.9811
HLA-A	PD | ieu-b-7	rs1611326	-0.2022	0.0822	0.013931826	-0.3634	-0.0410	0.8169	0.6953	0.9598
YBEY	PD | ieu-b-7	rs35210219	0.0574	0.0234	0.014149663	0.0115	0.1033	1.0591	1.0116	1.1088
SCART1	PD | ieu-b-7	rs2265903	-0.1031	0.0421	0.014244058	-0.1856	-0.0207	0.9020	0.8306	0.9795
SRPK1	PD | ieu-b-7	rs4340986	-0.2067	0.0846	0.014616363	-0.3726	-0.0408	0.8133	0.6890	0.9600
CHRNB1	PD | ieu-b-7	rs60488855	0.1582	0.0650	0.014900739	0.0309	0.2856	1.1714	1.0313	1.3305
APP	PD | ieu-b-7	rs225440	0.4731	0.1946	0.015041739	0.0917	0.8545	1.6049	1.0961	2.3501
TUBA1C	PD | ieu-b-7	rs2162644	0.1321	0.0545	0.015285972	0.0254	0.2389	1.1413	1.0257	1.2699
KCTD15	PD | ieu-b-7	rs28609628	-0.2727	0.1125	0.015329914	-0.4931	-0.0522	0.7613	0.6107	0.9491
MTHFS	PD | ieu-b-7	rs642697	0.1545	0.0639	0.015554088	0.0293	0.2797	1.1671	1.0298	1.3228
ZNF738	PD | ieu-b-7	rs59617671	0.0952	0.0394	0.015663354	0.0180	0.1723	1.0998	1.0181	1.1881
CAPZA1	PD | ieu-b-7	rs6671645	-0.1965	0.0822	0.016808826	-0.3576	-0.0354	0.8216	0.6994	0.9652
GAR1	PD | ieu-b-7	rs10365	-0.0732	0.0307	0.017096986	-0.1334	-0.0130	0.9294	0.8751	0.9870
RP11-89K11.1	PD | ieu-b-7	rs11633441	-0.1340	0.0565	0.017762328	-0.2448	-0.0232	0.8746	0.7828	0.9771
LDHAL6A	PD | ieu-b-7	rs3993293	0.0767	0.0324	0.017901604	0.0132	0.1403	1.0798	1.0133	1.1506
FAM89A	PD | ieu-b-7	rs4310430	0.1122	0.0474	0.01796303	0.0193	0.2051	1.1187	1.0194	1.2276
BTNL2	PD | ieu-b-7	rs35302878	0.0983	0.0417	0.018242657	0.0167	0.1800	1.1033	1.0168	1.1972
NUP85	PD | ieu-b-7	rs8065996	0.0800	0.0339	0.018349426	0.0135	0.1466	1.0833	1.0136	1.1579

AARS	PD | ieu-b-7	rs2070203	-0.2560	0.1088	0.01862558	-0.4692	-0.0427	0.7742	0.6255	0.9582
APPL2	PD | ieu-b-7	rs1201658	0.3428	0.1462	0.019018015	0.0563	0.6292	1.4088	1.0579	1.8761
POLR2M	PD | ieu-b-7	rs72745568	-0.1271	0.0544	0.019535593	-0.2338	-0.0204	0.8807	0.7916	0.9798
ARHGAP19	PD | ieu-b-7	rs701820	0.2840	0.1217	0.019679954	0.0453	0.5226	1.3284	1.0464	1.6864
AC009404.2	PD | ieu-b-7	rs6731372	-0.1129	0.0485	0.019858242	-0.2079	-0.0179	0.8932	0.8123	0.9823
CPEB1	PD | ieu-b-7	rs783534	-0.2356	0.1013	0.020040893	-0.4342	-0.0370	0.7901	0.6478	0.9636
CCDC153	PD | ieu-b-7	rs4936468	0.0947	0.0408	0.020241602	0.0148	0.1746	1.0993	1.0149	1.1908
RPL32P3	PD | ieu-b-7	rs7612378	-0.1617	0.0697	0.020258851	-0.2983	-0.0252	0.8507	0.7421	0.9751
FAM178B	PD | ieu-b-7	rs17119656	0.1833	0.0790	0.020292483	0.0285	0.3382	1.2012	1.0289	1.4024
RP11-665C16.6	PD | ieu-b-7	rs11547116	0.0577	0.0249	0.020569766	0.0089	0.1065	1.0594	1.0089	1.1123
RP11-91P24.7	PD | ieu-b-7	rs2032415	0.0853	0.0369	0.020725408	0.0130	0.1575	1.0890	1.0131	1.1706
TPCN2	PD | ieu-b-7	rs72932523	0.1325	0.0575	0.021103791	0.0199	0.2451	1.1417	1.0201	1.2778
AIFM2	PD | ieu-b-7	rs6480442	0.0693	0.0301	0.0212157	0.0103	0.1282	1.0717	1.0104	1.1368
ASB8	PD | ieu-b-7	rs10875746	-0.0928	0.0403	0.021336289	-0.1718	-0.0138	0.9114	0.8422	0.9863
CDIPT	PD | ieu-b-7	rs8335	-0.3556	0.1550	0.021768565	-0.6593	-0.0518	0.7008	0.5172	0.9495
LDHC	PD | ieu-b-7	rs58655904	0.0456	0.0199	0.022072634	0.0066	0.0846	1.0466	1.0066	1.0883
RP11-349H17.2	PD | ieu-b-7	rs4081488	-0.0874	0.0382	0.022134989	-0.1622	-0.0125	0.9163	0.8502	0.9875
MICU1	PD | ieu-b-7	rs7069357	-0.2360	0.1032	0.022163528	-0.4381	-0.0338	0.7898	0.6452	0.9668
LSAMP	PD | ieu-b-7	rs13100436	0.3412	0.1492	0.022193661	0.0488	0.6336	1.4067	1.0500	1.8845
LINC01749	PD | ieu-b-7	rs6122362	-0.1263	0.0553	0.022345424	-0.2347	-0.0179	0.8813	0.7908	0.9822
PEX6	PD | ieu-b-7	rs6941212	0.0497	0.0218	0.02237014	0.0071	0.0924	1.0510	1.0071	1.0968
RP11-320P7.2	PD | ieu-b-7	rs10879100	0.0859	0.0376	0.022525658	0.0121	0.1597	1.0897	1.0122	1.1731
CCT6P3	PD | ieu-b-7	rs4718188	-0.0413	0.0181	0.022702215	-0.0769	-0.0058	0.9595	0.9260	0.9942
CLNS1A	PD | ieu-b-7	rs35774637	-0.2055	0.0903	0.022787514	-0.3825	-0.0286	0.8142	0.6822	0.9718
TBC1D2	PD | ieu-b-7	rs942166	0.1106	0.0486	0.02290593	0.0153	0.2059	1.1170	1.0154	1.2286
QRICH1	PD | ieu-b-7	rs6806356	-0.1767	0.0777	0.022988131	-0.3291	-0.0244	0.8380	0.7196	0.9759
LINC00339	PD | ieu-b-7	rs10917120	0.0398	0.0175	0.023018482	0.0055	0.0740	1.0406	1.0055	1.0768

ERV3-1	PD | ieu-b-7	rs10949951	-0.0479	0.0211	0.023387449	-0.0893	-0.0065	0.9532	0.9145	0.9935
P4HTM	PD | ieu-b-7	rs9882443	0.2391	0.1056	0.023593705	0.0321	0.4462	1.2702	1.0326	1.5624
WBP1L	PD | ieu-b-7	rs2902548	0.1726	0.0764	0.023941637	0.0228	0.3224	1.1884	1.0230	1.3804
BACE2	PD | ieu-b-7	rs914187	-0.1945	0.0863	0.024311284	-0.3637	-0.0252	0.8233	0.6951	0.9751
PIGG	PD | ieu-b-7	rs61334630	0.1611	0.0719	0.025117986	0.0201	0.3021	1.1748	1.0203	1.3526
TUBG2	PD | ieu-b-7	rs2089118	0.1496	0.0668	0.025214845	0.0186	0.2806	1.1614	1.0188	1.3240
RSPH3	PD | ieu-b-7	rs6929297	-0.2108	0.0944	0.025550599	-0.3959	-0.0258	0.8099	0.6731	0.9746
RHCE	PD | ieu-b-7	rs3093638	0.0582	0.0261	0.025755075	0.0070	0.1093	1.0599	1.0071	1.1155
GSTA1	PD | ieu-b-7	rs9463833	0.1240	0.0556	0.025833902	0.0149	0.2330	1.1320	1.0151	1.2624
POPDC2	PD | ieu-b-7	rs13065543	0.0971	0.0436	0.025910878	0.0117	0.1826	1.1020	1.0117	1.2003
UPB1	PD | ieu-b-7	rs9624470	-0.0799	0.0359	0.026014938	-0.1503	-0.0096	0.9232	0.8604	0.9905
PLAC9	PD | ieu-b-7	rs11499000	-0.0667	0.0300	0.026139166	-0.1254	-0.0079	0.9355	0.8821	0.9921
NEDD9	PD | ieu-b-7	rs1810451	-0.2615	0.1180	0.026684538	-0.4928	-0.0302	0.7699	0.6109	0.9702
PPM1A	PD | ieu-b-7	rs10151922	0.1826	0.0826	0.026993053	0.0208	0.3444	1.2003	1.0210	1.4111
MAEL	PD | ieu-b-7	rs7551881	0.0571	0.0258	0.027161868	0.0064	0.1077	1.0587	1.0065	1.1137
ABO	PD | ieu-b-7	rs517414	-0.0664	0.0301	0.02730912	-0.1254	-0.0074	0.9357	0.8821	0.9926
TIE1	PD | ieu-b-7	rs2275180	-0.1570	0.0712	0.027511091	-0.2967	-0.0174	0.8547	0.7433	0.9828
CYP4A22	PD | ieu-b-7	rs74591829	-0.0753	0.0342	0.027604819	-0.1423	-0.0083	0.9275	0.8674	0.9917
INPP1	PD | ieu-b-7	rs2016037	0.0845	0.0384	0.027667396	0.0093	0.1597	1.0882	1.0093	1.1732
TMEM147-AS1	PD | ieu-b-7	rs12608794	-0.1563	0.0711	0.027986255	-0.2957	-0.0169	0.8553	0.7440	0.9833
DHRS4L2	PD | ieu-b-7	rs2273946	0.1178	0.0536	0.028103875	0.0126	0.2229	1.1250	1.0127	1.2497
DLG4	PD | ieu-b-7	rs3826408	0.3472	0.1582	0.028144503	0.0372	0.6572	1.4151	1.0379	1.9294
C1orf198	PD | ieu-b-7	rs12699	-0.3265	0.1488	0.028203474	-0.6182	-0.0349	0.7214	0.5389	0.9657
CPB2-AS1	PD | ieu-b-7	rs17600984	-0.0494	0.0225	0.028247446	-0.0936	-0.0053	0.9518	0.9107	0.9947
AC007773.2	PD | ieu-b-7	rs358548	-0.1177	0.0537	0.028590233	-0.2230	-0.0123	0.8890	0.8001	0.9878
TMEM50A	PD | ieu-b-7	rs3091242	0.1242	0.0567	0.028628004	0.0130	0.2354	1.1322	1.0131	1.2654
CERS5	PD | ieu-b-7	rs7964698	0.0734	0.0336	0.028803155	0.0076	0.1392	1.0762	1.0076	1.1493

ARNT	PD | ieu-b-7	rs11204752	-0.0978	0.0447	0.028812203	-0.1854	-0.0101	0.9069	0.8308	0.9899
RP11-403A3.3	PD | ieu-b-7	rs4773111	0.1530	0.0701	0.028972794	0.0157	0.2903	1.1653	1.0158	1.3368
KCNE3	PD | ieu-b-7	rs683144	-0.1097	0.0503	0.029067298	-0.2082	-0.0112	0.8961	0.8120	0.9889
DNAJC3	PD | ieu-b-7	rs11616456	-0.2108	0.0966	0.029159854	-0.4002	-0.0214	0.8099	0.6702	0.9788
ZNF781	PD | ieu-b-7	rs62108305	-0.0927	0.0425	0.029274123	-0.1761	-0.0093	0.9115	0.8386	0.9907
TESMIN	PD | ieu-b-7	rs4073958	-0.1262	0.0580	0.029541021	-0.2399	-0.0125	0.8814	0.7867	0.9875
RP11-742D12.2	PD | ieu-b-7	rs17708203	-0.1392	0.0640	0.029651016	-0.2646	-0.0137	0.8701	0.7675	0.9864
CHCHD2	PD | ieu-b-7	rs6593298	0.0956	0.0440	0.029659866	0.0094	0.1818	1.1003	1.0095	1.1993
CSE1L	PD | ieu-b-7	rs34778847	0.2409	0.1109	0.029766445	0.0236	0.4582	1.2724	1.0239	1.5813
PPIL6	PD | ieu-b-7	rs140355984	-0.0873	0.0402	0.029874891	-0.1661	-0.0085	0.9164	0.8470	0.9915
POLM	PD | ieu-b-7	rs10265836	-0.1285	0.0592	0.029879299	-0.2445	-0.0125	0.8794	0.7831	0.9875
UBE2W	PD | ieu-b-7	rs34584592	-0.3371	0.1552	0.029904853	-0.6413	-0.0328	0.7139	0.5266	0.9677
MAN2B2	PD | ieu-b-7	rs7659958	-0.1367	0.0630	0.029951459	-0.2602	-0.0133	0.8722	0.7709	0.9868
TBCD	PD | ieu-b-7	rs11869930	-0.2148	0.0990	0.030003779	-0.4088	-0.0208	0.8067	0.6645	0.9794
PLGRKT	PD | ieu-b-7	rs2146074	-0.1976	0.0911	0.030081615	-0.3762	-0.0190	0.8207	0.6865	0.9811
RRH	PD | ieu-b-7	rs7654660	0.0806	0.0372	0.030333027	0.0077	0.1535	1.0839	1.0077	1.1660
C9orf72	PD | ieu-b-7	rs774358	-0.0780	0.0361	0.030529037	-0.1487	-0.0073	0.9250	0.8618	0.9927
IPO8	PD | ieu-b-7	rs12370947	0.1015	0.0469	0.030565845	0.0095	0.1936	1.1069	1.0096	1.2136
XIRP2	PD | ieu-b-7	rs1450043	-0.1323	0.0613	0.030964062	-0.2526	-0.0121	0.8760	0.7768	0.9880
P3H2-AS1	PD | ieu-b-7	rs491074	-0.0792	0.0367	0.030968259	-0.1512	-0.0072	0.9239	0.8597	0.9928
PLPP7	PD | ieu-b-7	rs2478861	-0.2038	0.0946	0.031282125	-0.3893	-0.0183	0.8156	0.6775	0.9819
TRIM69	PD | ieu-b-7	rs17588305	0.0746	0.0346	0.031383659	0.0067	0.1425	1.0774	1.0067	1.1531
DAPK2	PD | ieu-b-7	rs332287	0.2101	0.0977	0.031518039	0.0186	0.4016	1.2338	1.0188	1.4943
CDADC1	PD | ieu-b-7	rs9568182	-0.1450	0.0675	0.03163946	-0.2773	-0.0128	0.8650	0.7578	0.9873
RP11-547D13.1	PD | ieu-b-7	rs28562431	-0.1518	0.0707	0.031828987	-0.2904	-0.0132	0.8591	0.7479	0.9869
NDUFB1	PD | ieu-b-7	rs7151932	0.1871	0.0875	0.032466954	0.0156	0.3586	1.2058	1.0158	1.4313
DNAL1	PD | ieu-b-7	rs12432044	0.1459	0.0683	0.032649786	0.0120	0.2797	1.1570	1.0121	1.3227

MTCH2	PD | ieu-b-7	rs11604680	0.0733	0.0343	0.032690047	0.0060	0.1405	1.0760	1.0061	1.1509
FBLN1	PD | ieu-b-7	rs2238809	0.2413	0.1131	0.032937745	0.0196	0.4630	1.2729	1.0197	1.5889
BNIP1	PD | ieu-b-7	rs72816168	0.0777	0.0364	0.033014917	0.0063	0.1491	1.0808	1.0063	1.1608
TYSND1	PD | ieu-b-7	rs2394645	0.1236	0.0580	0.033117718	0.0099	0.2373	1.1315	1.0099	1.2678
ANXA11	PD | ieu-b-7	rs11201989	-0.0830	0.0390	0.033210132	-0.1595	-0.0066	0.9203	0.8526	0.9934
GALK2	PD | ieu-b-7	rs11070699	-0.1034	0.0486	0.033308718	-0.1986	-0.0082	0.9018	0.8199	0.9919
RBM20	PD | ieu-b-7	rs2840311	0.0687	0.0323	0.033397429	0.0054	0.1321	1.0712	1.0054	1.1412
AC073343.13	PD | ieu-b-7	rs149502418	0.1161	0.0546	0.033586613	0.0090	0.2231	1.1231	1.0091	1.2500
OVOS2	PD | ieu-b-7	rs12580607	-0.1006	0.0474	0.033700562	-0.1935	-0.0078	0.9043	0.8241	0.9923
KCNJ3	PD | ieu-b-7	rs765439	0.1776	0.0837	0.033800053	0.0136	0.3417	1.1944	1.0137	1.4073
NCOA6	PD | ieu-b-7	rs6059916	-0.1858	0.0875	0.033800053	-0.3573	-0.0142	0.8305	0.6996	0.9859
HSPB8	PD | ieu-b-7	rs68062239	-0.2511	0.1184	0.033903956	-0.4830	-0.0191	0.7780	0.6169	0.9811
C18orf8	PD | ieu-b-7	rs6507720	0.1184	0.0559	0.03420497	0.0088	0.2279	1.1257	1.0089	1.2560
ANAPC4	PD | ieu-b-7	rs9991246	-0.1020	0.0482	0.034490872	-0.1965	-0.0074	0.9031	0.8216	0.9926
TRAPPC13	PD | ieu-b-7	rs12153235	0.2034	0.0963	0.034569939	0.0148	0.3921	1.2256	1.0149	1.4800
METTL21EP	PD | ieu-b-7	rs4150355	0.0737	0.0349	0.034810771	0.0053	0.1422	1.0765	1.0053	1.1528
CPSF2	PD | ieu-b-7	rs7149665	0.1260	0.0597	0.034867822	0.0090	0.2431	1.1343	1.0090	1.2752
AHSA1	PD | ieu-b-7	rs4369582	0.2119	0.1008	0.035426602	0.0145	0.4094	1.2361	1.0146	1.5059
OXGR1	PD | ieu-b-7	rs9556649	0.0861	0.0409	0.035426602	0.0059	0.1664	1.0899	1.0059	1.1810
RP11-386G11.10	PD | ieu-b-7	rs2016107	-0.0793	0.0378	0.035967266	-0.1535	-0.0052	0.9237	0.8577	0.9948
CYP39A1	PD | ieu-b-7	rs9455330	-0.1643	0.0784	0.036054055	-0.3178	-0.0107	0.8485	0.7277	0.9894
CTD-2313J17.5	PD | ieu-b-7	rs28435105	-0.0760	0.0363	0.036149832	-0.1472	-0.0049	0.9268	0.8631	0.9951
WDR25	PD | ieu-b-7	rs61990729	0.1219	0.0583	0.036457534	0.0077	0.2362	1.1297	1.0077	1.2664
TM7SF3	PD | ieu-b-7	rs146970669	-0.1235	0.0591	0.036514368	-0.2393	-0.0077	0.8838	0.7871	0.9923
ATRIP	PD | ieu-b-7	rs12497634	-0.0842	0.0406	0.03787239	-0.1637	-0.0047	0.9192	0.8490	0.9953
CADM2-AS1	PD | ieu-b-7	rs68058340	0.0674	0.0325	0.037966035	0.0037	0.1310	1.0697	1.0037	1.1400

CTD-2256P15.4	PD | ieu-b-7	rs3843011	-0.0515	0.0248	0.037986535	-0.1001	-0.0029	0.9498	0.9048	0.9972
STYXL1	PD | ieu-b-7	rs41301427	0.0649	0.0313	0.038091977	0.0036	0.1262	1.0670	1.0036	1.1345
KCNJ6	PD | ieu-b-7	rs857977	0.2167	0.1046	0.038197668	0.0118	0.4217	1.2420	1.0119	1.5245
LBHD1	PD | ieu-b-7	rs10792356	-0.0858	0.0416	0.039221479	-0.1674	-0.0042	0.9178	0.8459	0.9958
RP11-895K13.2	PD | ieu-b-7	rs837707	0.0981	0.0476	0.039238783	0.0048	0.1913	1.1031	1.0048	1.2109
CCND2-AS2	PD | ieu-b-7	rs10774058	0.1341	0.0651	0.039398541	0.0065	0.2617	1.1435	1.0065	1.2992
RP11-324I22.4	PD | ieu-b-7	rs11010224	0.1081	0.0525	0.039398541	0.0052	0.2110	1.1142	1.0053	1.2349
RP11-43F13.1	PD | ieu-b-7	rs58306596	0.0902	0.0438	0.039749945	0.0042	0.1761	1.0943	1.0042	1.1925
CCNB1IP1	PD | ieu-b-7	rs6575164	-0.0734	0.0358	0.040108322	-0.1436	-0.0033	0.9292	0.8663	0.9967
SHBG	PD | ieu-b-7	rs62059838	-0.0686	0.0335	0.040438416	-0.1343	-0.0030	0.9337	0.8743	0.9970
CLHC1	PD | ieu-b-7	rs6545475	-0.0728	0.0356	0.040444485	-0.1425	-0.0032	0.9297	0.8672	0.9968
MCUR1	PD | ieu-b-7	rs6904811	0.1089	0.0531	0.040529714	0.0047	0.2130	1.1150	1.0047	1.2374
SCIMP	PD | ieu-b-7	rs2439945	-0.0769	0.0376	0.040769009	-0.1506	-0.0032	0.9260	0.8602	0.9968
RP11-333E1.1	PD | ieu-b-7	rs2439945	-0.1165	0.0569	0.040769009	-0.2281	-0.0049	0.8900	0.7961	0.9951
MYLK2	PD | ieu-b-7	rs6060978	0.1486	0.0727	0.040826964	0.0062	0.2910	1.1602	1.0062	1.3378
OXTR	PD | ieu-b-7	rs53576	-0.1011	0.0495	0.041042199	-0.1981	-0.0041	0.9038	0.8203	0.9959
PNKD	PD | ieu-b-7	rs1877715	-0.2051	0.1005	0.041162958	-0.4020	-0.0082	0.8145	0.6690	0.9918
CHST10	PD | ieu-b-7	rs10193204	0.1530	0.0750	0.041428506	0.0059	0.3000	1.1653	1.0060	1.3498
ZNF641	PD | ieu-b-7	rs9788082	-0.0998	0.0489	0.041428506	-0.1957	-0.0039	0.9050	0.8222	0.9961
SULT1A1	PD | ieu-b-7	rs28729187	0.0919	0.0451	0.041646331	0.0035	0.1804	1.0963	1.0035	1.1977
P3H4	PD | ieu-b-7	rs1065029	-0.1528	0.0752	0.041984875	-0.3001	-0.0055	0.8583	0.7407	0.9945
INTS10	PD | ieu-b-7	rs111386636	-0.1379	0.0678	0.04205219	-0.2708	-0.0050	0.8712	0.7628	0.9951
SULT1A2	PD | ieu-b-7	rs2726033	-0.0613	0.0303	0.042760515	-0.1206	-0.0020	0.9405	0.8864	0.9980
ROPN1B	PD | ieu-b-7	rs7630213	-0.0690	0.0341	0.043143156	-0.1359	-0.0021	0.9333	0.8730	0.9979
CSDC2	PD | ieu-b-7	rs12484467	-0.0510	0.0253	0.043589385	-0.1006	-0.0015	0.9502	0.9043	0.9985
LSM10	PD | ieu-b-7	rs78467995	0.1621	0.0803	0.04367033	0.0046	0.3196	1.1760	1.0046	1.3765
SNX27	PD | ieu-b-7	rs6702842	0.0957	0.0474	0.043768161	0.0027	0.1887	1.1004	1.0027	1.2076

RP11-102M11.2	PD | ieu-b-7	rs35874192	-0.0848	0.0421	0.043935476	-0.1673	-0.0023	0.9187	0.8459	0.9977
LCN8	PD | ieu-b-7	rs2784068	0.0601	0.0299	0.044332961	0.0015	0.1187	1.0620	1.0015	1.1261
MGMT	PD | ieu-b-7	rs1008982	-0.0671	0.0334	0.044553091	-0.1325	-0.0016	0.9351	0.8759	0.9984
AHI1	PD | ieu-b-7	rs2207000	-0.0931	0.0464	0.044787528	-0.1840	-0.0022	0.9111	0.8319	0.9978
LINC00598	PD | ieu-b-7	rs2324499	-0.1402	0.0699	0.044957648	-0.2773	-0.0032	0.8692	0.7578	0.9969
POLR2D	PD | ieu-b-7	rs13012321	0.1304	0.0651	0.044973529	0.0029	0.2580	1.1393	1.0029	1.2943
RP11-384K6.8	PD | ieu-b-7	rs415653	0.0594	0.0297	0.045061317	0.0013	0.1175	1.0612	1.0013	1.1247
AC004540.4	PD | ieu-b-7	rs6461933	-0.0761	0.0380	0.045063094	-0.1505	-0.0017	0.9267	0.8603	0.9983
NIPAL2	PD | ieu-b-7	rs56215918	0.1727	0.0862	0.045200315	0.0037	0.3417	1.1885	1.0037	1.4073
ADAMTSL2	PD | ieu-b-7	rs9330463	0.1225	0.0612	0.045240067	0.0026	0.2424	1.1303	1.0026	1.2743
NME5	PD | ieu-b-7	rs62381760	-0.1201	0.0600	0.045500264	-0.2378	-0.0024	0.8868	0.7884	0.9976
MED24	PD | ieu-b-7	rs34342173	-0.1127	0.0563	0.045500264	-0.2231	-0.0023	0.8934	0.8000	0.9977
WNT3	PD | ieu-b-7	rs3933653	-0.0543	0.0271	0.045500264	-0.1075	-0.0011	0.9472	0.8981	0.9989
DPYSL5	PD | ieu-b-7	rs13025436	-0.1197	0.0599	0.045500264	-0.2370	-0.0024	0.8872	0.7890	0.9976
BCAP29	PD | ieu-b-7	rs2701678	0.2052	0.1026	0.045500264	0.0041	0.4062	1.2277	1.0041	1.5011
DNAJC5G	PD | ieu-b-7	rs74630263	0.1893	0.0949	0.045933923	0.0034	0.3753	1.2085	1.0034	1.4554
RHEBL1	PD | ieu-b-7	rs10459232	0.1465	0.0734	0.046135441	0.0025	0.2904	1.1577	1.0025	1.3370
TMPRSS5	PD | ieu-b-7	rs7114297	-0.0681	0.0342	0.046509397	-0.1352	-0.0011	0.9341	0.8735	0.9989
NOXRED1	PD | ieu-b-7	rs176778	0.0734	0.0369	0.046763125	0.0011	0.1458	1.0762	1.0011	1.1570
HFM1	PD | ieu-b-7	rs1823868	0.1138	0.0572	0.046813817	0.0016	0.2259	1.1205	1.0016	1.2535
CLGN	PD | ieu-b-7	rs11100639	-0.1412	0.0710	0.046871354	-0.2804	-0.0020	0.8683	0.7555	0.9981
E2F7	PD | ieu-b-7	rs11116780	0.0743	0.0375	0.047337993	0.0009	0.1478	1.0772	1.0009	1.1592
IP6K2	PD | ieu-b-7	rs72929172	0.0807	0.0407	0.047345845	0.0009	0.1604	1.0840	1.0009	1.1739
HARS	PD | ieu-b-7	rs2230361	0.2278	0.1149	0.047463254	0.0025	0.4531	1.2558	1.0025	1.5731
HDAC9	PD | ieu-b-7	rs10239376	0.3508	0.1771	0.047565123	0.0038	0.6979	1.4203	1.0038	2.0095
ZNF667	PD | ieu-b-7	rs10413986	0.1161	0.0587	0.047899285	0.0011	0.2311	1.1231	1.0011	1.2599
PNO1	PD | ieu-b-7	rs6742014	-0.1020	0.0516	0.048010811	-0.2031	-0.0009	0.9030	0.8162	0.9991

CDKL2	PD | ieu-b-7	rs35602225	0.1957	0.0990	0.048172288	0.0016	0.3898	1.2161	1.0016	1.4767
POMT2	PD | ieu-b-7	rs369901	0.2092	0.1060	0.048340939	0.0015	0.4169	1.2327	1.0015	1.5173
STEAP3	PD | ieu-b-7	rs838069	-0.1879	0.0952	0.048366061	-0.3744	-0.0013	0.8287	0.6877	0.9987
KCNJ16	PD | ieu-b-7	rs12942690	-0.2390	0.1211	0.048398095	-0.4763	-0.0017	0.7874	0.6211	0.9983
TRPM1	PD | ieu-b-7	rs3809579	-0.0486	0.0246	0.048457598	-0.0969	-0.0003	0.9526	0.9077	0.9997
RIPK1	PD | ieu-b-7	rs2272990	-0.1854	0.0942	0.049019612	-0.3700	-0.0008	0.8308	0.6907	0.9992
THSD7A	PD | ieu-b-7	rs67910311	-0.2214	0.1126	0.049320335	-0.4421	-0.0007	0.8014	0.6427	0.9993
TMCO1	PD | ieu-b-7	rs28504591	0.2403	0.1223	0.049339187	0.0007	0.4800	1.2717	1.0007	1.6161
AKAP3	PD | ieu-b-7	rs979877	-0.0614	0.0312	0.049434597	-0.1226	-0.0002	0.9405	0.8846	0.9998
PERP	PD | ieu-b-7	rs2152010	-0.1143	0.0582	0.049650478	-0.2284	-0.0002	0.8920	0.7958	0.9998

Supplementary Table 2. List of SNPs inputted to multimorbid3D pipeline to build PD-associated network

rs114138760	rs2042477	rs10513789	rs13117519	rs75859381	rs896435	rs10847864	rs2904880	rs117615688
rs35749011	rs11683001	rs873786	rs62333164	rs199351	rs10748818	rs11610045	rs11150601	rs11658976
rs76763715	rs57891859	rs34311866	rs1867598	rs76949143	rs72840788	rs9568188	rs6500328	rs61169879
rs6658353	rs1474055	rs4698412	rs26431	rs1293298	rs117896735	rs4771268	rs3104783	rs666463
rs11578699	rs73038319	rs34025766	rs11950533	rs620513	rs7938782	rs12147950	rs10221156	rs1941685
rs823118	rs6808178	rs6825004	rs4140646	rs2280104	rs12283611	rs11158026	rs12600861	rs12456492
rs11557080	rs12497850	rs4101061	rs9261484	rs2086641	rs3802920	rs3742785	rs12951632	rs8087969
rs4653767	rs55961674	rs6854006	rs112485576	rs13294100	rs76904798	rs979812	rs2269906	rs55818311
rs10797576	rs11707416	rs356182	rs12528068	rs10756907	rs34637584	rs2251086	rs850738	rs77351827
rs76116224	rs1450522	rs5019538	rs997368	rs6476434	rs7134559	rs6497339	rs62053943	rs2248244

Supplementary Table 3. Traits enriched for loci regulating genes in PD-causal network

SNP	trait	Target gene	PPI level	Bootstrap_pval	Bonferroni_adj_pval
rs12185268	PD	LINC02210	L0	0.012	2.60E-46
rs12185268	PD	KANSL1	L0	0.012	2.60E-46
rs12185268	Corticobasal degeneration	LINC02210	L0	0.004	0.01865476
rs12185268	Corticobasal degeneration	KANSL1	L0	0.004	0.01865476
rs12185268	Hemoglobin concentration	LINC02210	L0	0.012	0.01186528
rs12185268	Hemoglobin concentration	KANSL1	L0	0.012	0.01186528
rs6599389	PD	TMEM175	L0	0.012	2.60E-46
rs4698412	PD	CD38	L0	0.012	2.60E-46
rs4698412	PD or first degree relation to individual with PD	CD38	L0	0.008	3.34E-12
rs199515	PD	KANSL1	L0	0.012	2.60E-46
rs199515	Hemoglobin concentration	KANSL1	L0	0.012	0.01186528
rs199533	PD	KANSL1	L0	0.012	2.60E-46
rs199533	Alzheimer's disease (age of onset) in APOE e4 non-carriers	KANSL1	L0	0.002	0.044613754
rs199533	Hemoglobin concentration	KANSL1	L0	0.012	0.01186528
rs393152	PD	KANSL1	L0	0.012	2.60E-46
rs393152	PD	LINC02210	L0	0.012	2.60E-46
rs393152	Corticobasal degeneration	KANSL1	L0	0.004	0.01865476
rs393152	Corticobasal degeneration	LINC02210	L0	0.004	0.01865476
rs6532197	PD	MMRN1	L0	0.012	2.60E-46
rs4538475	PD	CD38	L0	0.012	2.60E-46
rs947211	PD	RAB29	L0	0.012	2.60E-46
rs6599388	PD	TMEM175	L0	0.012	2.60E-46
rs6599388	Waist-to-hip ratio adjusted for BMI	TMEM175	L0	0.03	1.60E-04
rs6599388	Waist-hip index	TMEM175	L0	0.016	8.64E-05

rs11724635	PD	CD38	L0	0.012	2.60E-46
rs2942168	PD	KANSL1	L0	0.012	2.60E-46
rs2942168	PD	LINC02210	L0	0.012	2.60E-46
rs11012	PD	LINC02210	L0	0.012	2.60E-46
rs113434679	PD	KANSL1	L0	0.012	2.60E-46
rs113434679	PD	LINC02210	L0	0.012	2.60E-46
rs113434679	General factor of neuroticism	KANSL1	L0	0.02	2.54E-10
rs113434679	General factor of neuroticism	LINC02210	L0	0.02	2.54E-10
rs62053943	PD	LINC02210	L0	0.012	2.60E-46
rs62053943	PD or first degree relation to individual with PD	LINC02210	L0	0.008	3.34E-12
rs62053943	White matter microstructure (radial diusivities)	LINC02210	L0	0.006	1.66E-08
rs62053943	General factor of neuroticism	LINC02210	L0	0.02	2.54E-10
rs11150601	PD	STX4	L0	0.012	2.60E-46
rs11150601	PD	PRSS36	L0	0.012	2.60E-46
rs11150601	PD or first degree relation to individual with PD	STX4	L0	0.008	3.34E-12
rs11150601	PD or first degree relation to individual with PD	PRSS36	L0	0.008	3.34E-12
rs28624974	PD	GPNMB	L0	0.012	2.60E-46
rs28624974	PD	NUPL2	L0	0.012	2.60E-46
rs113564729	PD	KANSL1	L0	0.012	2.60E-46
rs140820592	PD	STX4	L0	0.012	2.60E-46
rs6826785	PD	MMRN1	L0	0.012	2.60E-46
rs6449168	PD	CD38	L0	0.012	2.60E-46
rs415430	PD	KANSL1	L0	0.012	2.60E-46
rs199498	PD	KANSL1	L0	0.012	2.60E-46
rs14235	PD	KAT8	L0	0.012	2.60E-46
rs17649553	PD	KANSL1	L0	0.012	2.60E-46
rs199347	PD	GPNMB	L0	0.012	2.60E-46

rs199347	PD	NUPL2	L0	0.012	2.60E-46
rs199347	PD	KLHL7-AS1	L0	0.012	2.60E-46
rs17577094	PD	ARHGAP27	L0	0.012	2.60E-46
rs17577094	PD	KANSL1	L0	0.012	2.60E-46
rs7221167	PD	LINC02210	L0	0.012	2.60E-46
rs7221167	Waist-to-hip ratio adjusted for BMI	LINC02210	L0	0.03	1.60E-04
rs7221167	PD or first degree relation to individual with PD	LINC02210	L0	0.008	3.34E-12
rs7221167	General factor of neuroticism	LINC02210	L0	0.02	2.54E-10
rs199501	PD	KANSL1	L0	0.012	2.60E-46
rs35541465	PD	TMEM175	L0	0.012	2.60E-46
rs4266290	PD	CD38	L0	0.012	2.60E-46
rs365825	PD	KANSL1	L0	0.012	2.60E-46
rs365825	PD	LINC02210	L0	0.012	2.60E-46
rs9303525	Intracranial volume	ARHGAP27	L0	0.002	0.044613754
rs9303525	Intracranial volume	KANSL1	L0	0.002	0.044613754
rs199525	Intracranial volume	KANSL1	L0	0.002	0.044613754
rs199525	Feeling guilty	KANSL1	L0	0.006	7.82E-07
rs111433752	Neuroticism	LINC02210	L0	0.016	1.80E-16
rs62064595	Neuroticism	ARHGAP27	L0	0.016	1.80E-16
rs62064595	Waist-to-hip ratio adjusted for BMI	ARHGAP27	L0	0.03	1.60E-04
rs62064595	Waist-hip index	ARHGAP27	L0	0.016	8.64E-05
rs1230106	Neuroticism	LINC02210	L0	0.016	1.80E-16
rs1724422	Neuroticism	LINC02210	L0	0.016	1.80E-16
rs1724422	Depressed affect	LINC02210	L0	0.008	3.81E-12
rs61572747	Neuroticism	ARHGAP27	L0	0.016	1.80E-16
rs61572747	Neuroticism	KANSL1	L0	0.016	1.80E-16
rs61572747	Depressed affect	ARHGAP27	L0	0.008	3.81E-12

rs61572747	Depressed affect	KANSL1	L0	0.008	3.81E-12
rs61572747	White matter microstructure (mean diusivities)	ARHGAP27	L0	0.012	1.67E-09
rs61572747	White matter microstructure (mean diusivities)	KANSL1	L0	0.012	1.67E-09
rs61572747	White matter microstructure (radial diusivities)	ARHGAP27	L0	0.006	1.66E-08
rs61572747	White matter microstructure (radial diusivities)	KANSL1	L0	0.006	1.66E-08
rs61572747	General cognitive ability	ARHGAP27	L0	0.01	0.001427436
rs61572747	General cognitive ability	KANSL1	L0	0.01	0.001427436
rs61572747	General factor of neuroticism	ARHGAP27	L0	0.02	2.54E-10
rs61572747	General factor of neuroticism	KANSL1	L0	0.02	2.54E-10
rs113100008	Neuroticism	LINC02210	L0	0.016	1.80E-16
rs35909029	Neuroticism	LINC02210	L0	0.016	1.80E-16
rs35909029	Depressed affect	LINC02210	L0	0.008	3.81E-12
rs34186148	Neuroticism	LINC02210	L0	0.016	1.80E-16
rs34186148	Depressed affect	LINC02210	L0	0.008	3.81E-12
rs34186148	White matter microstructure (mean diusivities)	LINC02210	L0	0.012	1.67E-09
rs34186148	White matter microstructure (radial diusivities)	LINC02210	L0	0.006	1.66E-08
rs4076453	Neuroticism	LINC02210	L0	0.016	1.80E-16
rs3785879	Neuroticism	KANSL1	L0	0.016	1.80E-16
rs3785879	Worry	KANSL1	L0	0.008	4.30E-06
rs2435207	Neuroticism	KANSL1	L0	0.016	1.80E-16
rs2016034	Neuroticism	ARHGAP27	L0	0.016	1.80E-16
rs2016034	Neuroticism	KANSL1	L0	0.016	1.80E-16
rs199447	Neuroticism	KANSL1	L0	0.016	1.80E-16
rs77804065	Neuroticism	LINC02210	L0	0.016	1.80E-16
rs77804065	Neuroticism	KANSL1	L0	0.016	1.80E-16
rs77804065	Maximum habitual alcohol consumption	LINC02210	L0	0.008	0.040609036
rs77804065	Maximum habitual alcohol consumption	KANSL1	L0	0.008	0.040609036

rs77804065	Feeling guilty	LINC02210	L0	0.006	7.82E-07
rs77804065	Feeling guilty	KANSL1	L0	0.006	7.82E-07
rs7225384	Neuroticism	LINC02210	L0	0.016	1.80E-16
rs7225384	Depressed affect	LINC02210	L0	0.008	3.81E-12
rs242947	Neuroticism	LINC02210	L0	0.016	1.80E-16
rs242947	Depressed affect	LINC02210	L0	0.008	3.81E-12
rs242947	General factor of neuroticism	LINC02210	L0	0.02	2.54E-10
rs754593	Neuroticism	LINC02210	L0	0.016	1.80E-16
rs754593	Waist-to-hip ratio adjusted for BMI	LINC02210	L0	0.03	1.60E-04
rs754593	Depressed affect	LINC02210	L0	0.008	3.81E-12
rs754593	Waist-hip index	LINC02210	L0	0.016	8.64E-05
rs754593	General cognitive ability	LINC02210	L0	0.01	0.001427436
rs754593	General factor of neuroticism	LINC02210	L0	0.02	2.54E-10
rs7225002	Neuroticism	ARHGAP27	L0	0.016	1.80E-16
rs7225002	Depressed affect	ARHGAP27	L0	0.008	3.81E-12
rs7225002	PD or first degree relation to individual with PD	ARHGAP27	L0	0.008	3.34E-12
rs7225002	Waist-hip index	ARHGAP27	L0	0.016	8.64E-05
rs7225002	White matter microstructure (axial diusivities)	ARHGAP27	L0	0.012	2.57E-07
rs7225002	General factor of neuroticism	ARHGAP27	L0	0.02	2.54E-10
rs2732708	Neuroticism	KANSL1	L0	0.016	1.80E-16
rs2732708	Neuroticism	LINC02210	L0	0.016	1.80E-16
rs2732708	Feeling miserable	KANSL1	L0	0.004	0.002266556
rs2732708	Feeling miserable	LINC02210	L0	0.004	0.002266556
rs17652520	Neuroticism	KANSL1	L0	0.016	1.80E-16
rs2214258	Neuroticism	KANSL1	L0	0.016	1.80E-16
rs2214258	Neuroticism	LINC02210	L0	0.016	1.80E-16
rs62062288	Neuroticism	KANSL1	L0	0.016	1.80E-16

rs62062288	Feeling guilty	KANSL1	L0	0.006	7.82E-07
rs12944712	Neuroticism	LINC02210	L0	0.016	1.80E-16
rs55955207	Neuroticism	KANSL1	L0	0.016	1.80E-16
rs55955207	Neuroticism	LINC02210	L0	0.016	1.80E-16
rs61667602	Maximum habitual alcohol consumption	KANSL1	L0	0.008	0.040609036
rs61667602	Maximum habitual alcohol consumption	LINC02210	L0	0.008	0.040609036
rs570285046	Maximum habitual alcohol consumption	KANSL1	L0	0.008	0.040609036
rs570285046	Maximum habitual alcohol consumption	LINC02210	L0	0.008	0.040609036
rs12185233	Waist-to-hip ratio adjusted for BMI	LINC02210	L0	0.03	1.60E-04
rs12185233	Waist-to-hip ratio adjusted for BMI	KANSL1	L0	0.03	1.60E-04
rs2279183	Waist-to-hip ratio adjusted for BMI	TMEM175	L0	0.03	1.60E-04
rs11724804	Waist-to-hip ratio adjusted for BMI	TMEM175	L0	0.03	1.60E-04
rs60521849	Waist-to-hip ratio adjusted for BMI	KANSL1	L0	0.03	1.60E-04
rs10514889	Waist-to-hip ratio adjusted for BMI	KANSL1	L0	0.03	1.60E-04
rs10514889	Waist-hip index	KANSL1	L0	0.016	8.64E-05
rs12051901	Waist-to-hip ratio adjusted for BMI	KANSL1	L0	0.03	1.60E-04
rs12051901	Waist-hip index	KANSL1	L0	0.016	8.64E-05
rs9904766	Waist-to-hip ratio adjusted for BMI	ARHGAP27	L0	0.03	1.60E-04
rs9904766	Waist-to-hip ratio adjusted for BMI	KANSL1	L0	0.03	1.60E-04
rs9904766	Waist-hip index	ARHGAP27	L0	0.016	8.64E-05
rs9904766	Waist-hip index	KANSL1	L0	0.016	8.64E-05
rs4510068	Waist-to-hip ratio adjusted for BMI	ARHGAP27	L0	0.03	1.60E-04
rs4510068	Waist-hip index	ARHGAP27	L0	0.016	8.64E-05
rs62074125	Waist-to-hip ratio adjusted for BMI	KANSL1	L0	0.03	1.60E-04
rs62074125	Waist-hip index	KANSL1	L0	0.016	8.64E-05
rs62074125	Reaction time	KANSL1	L0	0.008	2.52E-05
rs11653367	Waist-to-hip ratio adjusted for BMI	KANSL1	L0	0.03	1.60E-04

rs11653367	Waist-hip index	KANSL1	L0	0.016	8.64E-05
rs12452064	Waist-to-hip ratio adjusted for BMI	KANSL1	L0	0.03	1.60E-04
rs12452064	Waist-hip index	KANSL1	L0	0.016	8.64E-05
rs9303471	Waist-to-hip ratio adjusted for BMI	LINC02210	L0	0.03	1.60E-04
rs7218457	Waist-to-hip ratio adjusted for BMI	LINC02210	L0	0.03	1.60E-04
rs62063286	Waist-to-hip ratio adjusted for BMI	KANSL1	L0	0.03	1.60E-04
rs6815138	Waist-to-hip ratio adjusted for BMI	TMEM175	L0	0.03	1.60E-04
rs6815138	Waist-hip index	TMEM175	L0	0.016	8.64E-05
rs9895436	Waist-to-hip ratio adjusted for BMI	LINC02210	L0	0.03	1.60E-04
rs9895436	Waist-hip index	LINC02210	L0	0.016	8.64E-05
rs3072094	Waist-to-hip ratio adjusted for BMI	LINC02210	L0	0.03	1.60E-04
rs3072094	Waist-to-hip ratio adjusted for BMI	KANSL1	L0	0.03	1.60E-04
rs3072094	Waist-hip index	LINC02210	L0	0.016	8.64E-05
rs3072094	Waist-hip index	KANSL1	L0	0.016	8.64E-05
rs11656151	Waist-to-hip ratio adjusted for BMI	KANSL1	L0	0.03	1.60E-04
rs11656151	Waist-hip index	KANSL1	L0	0.016	8.64E-05
rs199503	Waist-to-hip ratio adjusted for BMI	KANSL1	L0	0.03	1.60E-04
rs199503	Waist-hip index	KANSL1	L0	0.016	8.64E-05
rs62057061	Depressed affect	LINC02210	L0	0.008	3.81E-12
rs62057061	Hemoglobin concentration	LINC02210	L0	0.012	0.01186528
rs12938031	Depressed affect	LINC02210	L0	0.008	3.81E-12
rs12938031	Worry	LINC02210	L0	0.008	4.30E-06
rs12938031	General cognitive ability	LINC02210	L0	0.01	0.001427436
rs12938031	General factor of neuroticism	LINC02210	L0	0.02	2.54E-10
rs199505	Depressed affect	KANSL1	L0	0.008	3.81E-12
rs11557080	PD or first degree relation to individual with PD	RAB29	L0	0.008	3.34E-12
rs199351	PD or first degree relation to individual with PD	GPNMB	L0	0.008	3.34E-12

rs199351	PD or first degree relation to individual with PD	NUPL2	L0	0.008	3.34E-12
rs117615688	PD or first degree relation to individual with PD	LINC02210	L0	0.008	3.34E-12
rs11658976	PD or first degree relation to individual with PD	KANSL1	L0	0.008	3.34E-12
rs199453	PD or first degree relation to individual with PD	KANSL1	L0	0.008	3.34E-12
rs2435210	Worry	KANSL1	L0	0.008	4.30E-06
rs59735493	Worry	KAT8	L0	0.008	4.30E-06
rs62064641	Worry	LINC02210	L0	0.008	4.30E-06
rs62064641	Worry	ARHGAP27	L0	0.008	4.30E-06
rs62064641	Worry	KANSL1	L0	0.008	4.30E-06
rs7220839	Worry	LINC02210	L0	0.008	4.30E-06
rs9896243	Worry	KANSL1	L0	0.008	4.30E-06
rs12945855	Feeling miserable	ADORA2B	L0	0.004	0.002266556
rs12945855	Feeling miserable	TTC19	L0	0.004	0.002266556
rs12945855	Feeling miserable	ZSWIM7	L0	0.004	0.002266556
rs56280951	Feeling miserable	KANSL1	L0	0.004	0.002266556
rs56280951	Feeling miserable	LINC02210	L0	0.004	0.002266556
rs17577369	Feeling miserable	KANSL1	L0	0.004	0.002266556
rs17577369	Feeling miserable	LINC02210	L0	0.004	0.002266556
rs17577369	Feeling miserable	ARHGAP27	L0	0.004	0.002266556
rs241036	Experiencing mood swings	KANSL1	L0	0.008	6.98E-05
rs241036	Experiencing mood swings	LINC02210	L0	0.008	6.98E-05
rs241036	Experiencing mood swings	ARHGAP27	L0	0.008	6.98E-05
rs55657917	Experiencing mood swings	KANSL1	L0	0.008	6.98E-05
rs55657917	Experiencing mood swings	LINC02210	L0	0.008	6.98E-05
rs55657917	Feeling hurt	KANSL1	L0	0.006	5.97E-04
rs55657917	Feeling hurt	LINC02210	L0	0.006	5.97E-04
rs79857651	Experiencing mood swings	KANSL1	L0	0.008	6.98E-05

rs112333322	Experiencing mood swings	KANSL1	L0	0.008	6.98E-05
rs112333322	Experiencing mood swings	LINC02210	L0	0.008	6.98E-05
rs17665188	Experiencing mood swings	KANSL1	L0	0.008	6.98E-05
rs62055701	Irritable mood	LINC02210	L0	0.012	1.09E-06
rs62055701	Irritable mood	KANSL1	L0	0.012	1.09E-06
rs2106785	Irritable mood	LINC02210	L0	0.012	1.09E-06
rs2106785	Irritable mood	KANSL1	L0	0.012	1.09E-06
rs17650842	Irritable mood	KANSL1	L0	0.012	1.09E-06
rs17661015	Irritable mood	KANSL1	L0	0.012	1.09E-06
rs17661015	Irritable mood	LINC02210	L0	0.012	1.09E-06
rs17661015	Irritable mood	ARHGAP27	L0	0.012	1.09E-06
rs17661015	Feeling hurt	KANSL1	L0	0.006	5.97E-04
rs17661015	Feeling hurt	LINC02210	L0	0.006	5.97E-04
rs17661015	Feeling hurt	ARHGAP27	L0	0.006	5.97E-04
rs2261201	Irritable mood	KANSL1	L0	0.012	1.09E-06
rs70600	Irritable mood	KANSL1	L0	0.012	1.09E-06
rs62061733	Feeling hurt	KANSL1	L0	0.006	5.97E-04
rs2696668	Feeling hurt	KANSL1	L0	0.006	5.97E-04
rs2696668	Feeling hurt	LINC02210	L0	0.006	5.97E-04
rs4471723	Feeling guilty	KANSL1	L0	0.006	7.82E-07
rs2696532	Feeling guilty	KANSL1	L0	0.006	7.82E-07
rs2696532	Feeling guilty	LINC02210	L0	0.006	7.82E-07
rs4320134	ADP-ribosyl cyclase/cyclic ADP-ribose hydrolase 2 levels	CD38	L0	0.004	4.77E-05
rs4698119	ADP-ribosyl cyclase/cyclic ADP-ribose hydrolase 2 levels	CD38	L0	0.004	4.77E-05
rs1110258	ADP-ribosyl cyclase/cyclic ADP-ribose hydrolase 2 levels	CD38	L0	0.004	4.77E-05
rs1789	ADP-ribosyl cyclase/cyclic ADP-ribose hydrolase 2 levels	FAM200B	L0	0.004	4.77E-05
rs10445365	IDP dMRI TBSS ICVF Anterior corona radiata R	LINC02210	L0	0.002	0.03474041

rs10445365	IDP dMRI TBSS ICVF Anterior corona radiata R	KANSL1	L0	0.002	0.03474041
rs10445365	IDP dMRI ProbtrackX MD atr l	LINC02210	L0	0.002	0.003744143
rs10445365	IDP dMRI ProbtrackX MD atr l	KANSL1	L0	0.002	0.003744143
rs79724577	IDP dMRI TBSS ICVF Anterior corona radiata R	ARHGAP27	L0	0.002	0.03474041
rs79724577	IDP dMRI ProbtrackX MD atr l	ARHGAP27	L0	0.002	0.003744143
rs142642115	BA-exvivo rh area V2	LINC02210	L0	0.004	0.012451144
rs2696606	BA-exvivo rh area V2	KANSL1	L0	0.004	0.012451144
rs2696606	BA-exvivo rh area V2	LINC02210	L0	0.004	0.012451144
rs112123127	IDP dMRI TBSS MD Anterior limb of internal capsule R	KANSL1	L0	0.004	0.007479481
rs112123127	IDP dMRI TBSS MD Anterior limb of internal capsule R	LINC02210	L0	0.004	0.007479481
rs2732650	IDP dMRI TBSS MD Anterior limb of internal capsule R	KANSL1	L0	0.004	0.007479481
rs2732650	IDP dMRI TBSS MD Anterior limb of internal capsule R	LINC02210	L0	0.004	0.007479481
rs2696518	IDP dMRI ProbtrackX L1 str r	KANSL1	L0	0.002	0.003744143
rs417968	IDP dMRI ProbtrackX L1 str r	KANSL1	L0	0.002	0.003744143
rs417968	IDP dMRI ProbtrackX L1 str r	LINC02210	L0	0.002	0.003744143
rs417968	IDP dMRI ProbtrackX L1 str r	ARHGAP27	L0	0.002	0.003744143
rs10262243	Serum levels of protein GPNMB	GPNMB	L0	0.004	0.012451144
rs10262243	Serum levels of protein GPNMB	KLHL7-AS1	L0	0.004	0.012451144
rs10262243	Serum levels of protein GPNMB	NUPL2	L0	0.004	0.012451144
rs10262243	Transmembrane glycoprotein NMB levels (GPNMB.8606.39.3)	GPNMB	L0	0.004	0.01865476
rs10262243	Transmembrane glycoprotein NMB levels (GPNMB.8606.39.3)	KLHL7-AS1	L0	0.004	0.01865476
rs10262243	Transmembrane glycoprotein NMB levels (GPNMB.8606.39.3)	NUPL2	L0	0.004	0.01865476
rs1881203	Serum levels of protein GPNMB	GPNMB	L0	0.004	0.012451144
rs1881203	Serum levels of protein GPNMB	KLHL7-AS1	L0	0.004	0.012451144
rs1881203	Serum levels of protein GPNMB	NUPL2	L0	0.004	0.012451144
rs12373123	Alzheimer's disease (age of onset) in APOE e4 non-carriers	LINC02210	L0	0.002	0.044613754
rs12373123	Alzheimer's disease (age of onset) in APOE e4 non-carriers	KANSL1	L0	0.002	0.044613754

rs10250602	Transmembrane glycoprotein NMB levels (GPNMB.8606.39.3)	GPNMB	L0	0.004	0.01865476
rs242944	Waist-hip index	LINC02210	L0	0.016	8.64E-05
rs9898399	Waist-hip index	LINC02210	L0	0.016	8.64E-05
rs9896485	Waist-hip index	KANSL1	L0	0.016	8.64E-05
rs3822020	Alpha-L-iduronidase levels (IDUA.3169.70.2)	TMEM175	L0	0.008	7.67E-05
rs13101828	Alpha-L-iduronidase levels (IDUA.3169.70.2)	TMEM175	L0	0.008	7.67E-05
rs4690203	Alpha-L-iduronidase levels (IDUA.3169.70.2)	TMEM175	L0	0.008	7.67E-05
rs35519908	White matter microstructure (mean diusivities)	LINC02210	L0	0.012	1.67E-09
rs55938136	White matter microstructure (mean diusivities)	LINC02210	L0	0.012	1.67E-09
rs55938136	White matter microstructure (axial diusivities)	LINC02210	L0	0.012	2.57E-07
rs55938136	White matter microstructure (radial diusivities)	LINC02210	L0	0.006	1.66E-08
rs55938136	White matter microstructure (fractional anisotropy)	LINC02210	L0	0.016	0.030818055
rs2696466	White matter microstructure (mean diusivities)	LINC02210	L0	0.012	1.67E-09
rs2696466	White matter microstructure (radial diusivities)	LINC02210	L0	0.006	1.66E-08
rs34081316	White matter microstructure (mean diusivities)	KANSL1	L0	0.012	1.67E-09
rs34081316	White matter microstructure (axial diusivities)	KANSL1	L0	0.012	2.57E-07
rs34081316	White matter microstructure (radial diusivities)	KANSL1	L0	0.006	1.66E-08
rs3890609	White matter microstructure (mean diusivities)	KANSL1	L0	0.012	1.67E-09
rs3890609	White matter microstructure (mean diusivities)	LINC02210	L0	0.012	1.67E-09
rs3890609	White matter microstructure (axial diusivities)	KANSL1	L0	0.012	2.57E-07
rs3890609	White matter microstructure (axial diusivities)	LINC02210	L0	0.012	2.57E-07
rs3890609	White matter microstructure (radial diusivities)	KANSL1	L0	0.006	1.66E-08
rs3890609	White matter microstructure (radial diusivities)	LINC02210	L0	0.006	1.66E-08
rs113562005	White matter microstructure (mean diusivities)	ARHGAP27	L0	0.012	1.67E-09
rs113562005	White matter microstructure (axial diusivities)	ARHGAP27	L0	0.012	2.57E-07
rs368919625	White matter microstructure (mean diusivities)	KANSL1	L0	0.012	1.67E-09
rs369762169	White matter microstructure (mean diusivities)	LINC02210	L0	0.012	1.67E-09

rs369762169	White matter microstructure (mean diusivities)	ARHGAP27	L0	0.012	1.67E-09
rs242562	Reaction time	KANSL1	L0	0.008	2.52E-05
rs6503453	Reaction time	KANSL1	L0	0.008	2.52E-05
rs8079215	Reaction time	KANSL1	L0	0.008	2.52E-05
rs2471738	Reaction time	KANSL1	L0	0.008	2.52E-05
rs2471738	General cognitive ability	KANSL1	L0	0.01	0.001427436
rs117754181	Reaction time	KANSL1	L0	0.008	2.52E-05
rs4988900	Reaction time	KANSL1	L0	0.008	2.52E-05
rs12952704	Reaction time	KANSL1	L0	0.008	2.52E-05
rs10775404	Reaction time	KANSL1	L0	0.008	2.52E-05
rs2532345	Reaction time	KANSL1	L0	0.008	2.52E-05
rs564681262	White matter microstructure (axial diusivities)	LINC02210	L0	0.012	2.57E-07
rs550530305	White matter microstructure (axial diusivities)	KANSL1	L0	0.012	2.57E-07
rs4763	White matter microstructure (axial diusivities)	ARHGAP27	L0	0.012	2.57E-07
rs147431626	White matter microstructure (radial diusivities)	KANSL1	L0	0.006	1.66E-08
rs548785130	White matter microstructure (radial diusivities)	KANSL1	L0	0.006	1.66E-08
rs577386308	White matter microstructure (radial diusivities)	KANSL1	L0	0.006	1.66E-08
rs62063281	Osteoarthrosis (time to event)	KANSL1	L0	0.002	0.012451144
rs2532386	Osteoarthrosis (time to event)	KANSL1	L0	0.002	0.012451144
rs2532386	Osteoarthrosis (time to event)	LINC02210	L0	0.002	0.012451144
rs3785884	Hemoglobin concentration	KANSL1	L0	0.012	0.01186528
rs3785884	Hemoglobin concentration	LINC02210	L0	0.012	0.01186528
rs58879558	Hemoglobin concentration	KANSL1	L0	0.012	0.01186528
rs17563683	Hemoglobin concentration	LINC02210	L0	0.012	0.01186528
rs17563683	Hemoglobin concentration	KANSL1	L0	0.012	0.01186528
rs2942164	Hemoglobin concentration	KANSL1	L0	0.012	0.01186528
rs2942164	Hemoglobin concentration	LINC02210	L0	0.012	0.01186528

rs12939753	Hemoglobin concentration	KANSL1	L0	0.012	0.01186528
rs2696671	Hemoglobin concentration	KANSL1	L0	0.012	0.01186528
rs2696671	Hemoglobin concentration	LINC02210	L0	0.012	0.01186528
rs2696524	White matter microstructure (fractional anisotropy)	KANSL1	L0	0.016	0.030818055
rs10445367	White matter microstructure (fractional anisotropy)	LINC02210	L0	0.016	0.030818055
rs10445367	White matter microstructure (fractional anisotropy)	KANSL1	L0	0.016	0.030818055
rs62062797	White matter microstructure (fractional anisotropy)	KANSL1	L0	0.016	0.030818055
rs80028338	White matter microstructure (fractional anisotropy)	LINC02210	L0	0.016	0.030818055
rs80028338	White matter microstructure (fractional anisotropy)	KANSL1	L0	0.016	0.030818055
rs242559	General cognitive ability	KANSL1	L0	0.01	0.001427436
rs1724425	General cognitive ability	LINC02210	L0	0.01	0.001427436
rs1724425	General factor of neuroticism	LINC02210	L0	0.02	2.54E-10
rs3843738	General cognitive ability	LINC02210	L0	0.01	0.001427436
rs3843738	General factor of neuroticism	LINC02210	L0	0.02	2.54E-10
rs9907781	General cognitive ability	KANSL1	L0	0.01	0.001427436
rs242934	General cognitive ability	LINC02210	L0	0.01	0.001427436
rs35076622	General cognitive ability	LINC02210	L0	0.01	0.001427436
rs35076622	General factor of neuroticism	LINC02210	L0	0.02	2.54E-10
rs1158660	General cognitive ability	KANSL1	L0	0.01	0.001427436
rs1158660	General cognitive ability	LINC02210	L0	0.01	0.001427436
rs35116560	General cognitive ability	LINC02210	L0	0.01	0.001427436
rs62064598	General factor of neuroticism	ARHGAP27	L0	0.02	2.54E-10
rs10876432	Bone mineral density (spine)	AAAS	L1	0.012	3.34E-04
rs2016266	Bone mineral density (spine)	AAAS	L1	0.012	3.34E-04
rs2016266	Lumbar spine bone mineral density	AAAS	L1	0.002	0.005758665
rs2272313	Bone mineral density (spine)	AAAS	L1	0.012	3.34E-04
rs2272313	Lumbar spine bone mineral density	AAAS	L1	0.002	0.005758665

rs10747666	Bone mineral density (spine)	AAAS	L1	0.012	3.34E-04
rs910873	Melanoma	CHMP4B	L1	0.014	0.039693137
rs910873	Non-melanoma skin cancer	CHMP4B	L1	0.038	0.009558168
rs139122788	Melanoma	ATM	L1	0.014	0.039693137
rs17401449	Melanoma	CHMP4B	L1	0.014	0.039693137
rs17401449	Low tan response	CHMP4B	L1	0.016	0.009030327
rs17401449	Non-melanoma skin cancer	CHMP4B	L1	0.038	0.009558168
rs3755955	Lumbar spine bone mineral density	GAK	L1	0.002	0.005758665
rs78382342	Low tan response	CHMP4B	L1	0.016	0.009030327
rs4911466	Low tan response	CHMP4B	L1	0.016	0.009030327
rs4911466	Non-melanoma skin cancer	CHMP4B	L1	0.038	0.009558168
rs111930714	Non-melanoma skin cancer	CHMP4B	L1	0.038	0.009558168
rs56396408	Total body bone mineral density (age 45-60)	GAK	L1	0.002	0.023815263
rs7398996	Total body bone mineral density (age 45-60)	AAAS	L1	0.002	0.023815263
rs13101828	Alpha-L-iduronidase levels (IDUA.3169.70.2)	GAK	L1	0.008	4.16E-06
rs4690203	Alpha-L-iduronidase levels (IDUA.3169.70.2)	GAK	L1	0.008	4.16E-06
rs56079856	Alpha-L-iduronidase levels (IDUA.3169.70.2)	GAK	L1	0.008	4.16E-06
rs12785878	Vitamin D insufficiency	NADSYN1	L2	0.006	0.026453767
rs4944958	Vitamin D insufficiency	NADSYN1	L2	0.006	0.026453767
rs3831470	Vitamin D insufficiency	NADSYN1	L2	0.006	0.026453767
rs11602954	Mean platelet volume	SIRT3	L2	0.01	0.00133101
rs1172130	Mean platelet volume	RBBP5	L2	0.01	0.00133101
rs17655730	Mean platelet volume	SIRT3	L2	0.01	0.00133101
rs55781332	Mean platelet volume	SIRT3	L2	0.01	0.00133101
rs9660992	Mean platelet volume	RBBP5	L2	0.01	0.00133101
rs61822568	Mean platelet volume	RBBP5	L2	0.01	0.00133101
rs11078559	Mean platelet volume	NUP88	L2	0.01	0.00133101

rs11604127	Mean platelet volume	SIRT3	L2	0.01	0.00133101
rs6992820	Mean platelet volume	NRG1	L2	0.01	0.00133101
rs3851296	Mean platelet volume	RBBP5	L2	0.01	0.00133101
rs11607019	Mean platelet volume	SIRT3	L2	0.01	0.00133101
rs13259933	Mean platelet volume	NRG1	L2	0.01	0.00133101
rs6985353	Mean platelet volume	NRG1	L2	0.01	0.00133101
rs528863805	Mean platelet volume	INO80E	L2	0.01	0.00133101
rs1172129	Mean platelet volume	RBBP5	L2	0.01	0.00133101
rs200454003	Serum 25-Hydroxyvitamin D levels	NADSYN1	L2	0.006	1.47E-04
rs7938885	Serum 25-Hydroxyvitamin D levels	NADSYN1	L2	0.006	1.47E-04
rs12800438	Serum 25-Hydroxyvitamin D levels	NADSYN1	L2	0.006	1.47E-04
rs12278461	Serum 25-Hydroxyvitamin D levels	NADSYN1	L2	0.006	1.47E-04
rs2297991	Serum 25-Hydroxyvitamin D levels	VTI1A	L2	0.006	1.47E-04
rs1894100	Serum 25-Hydroxyvitamin D levels	NADSYN1	L2	0.006	1.47E-04
rs1790373	Serum 25-Hydroxyvitamin D levels	NADSYN1	L2	0.006	1.47E-04
rs11233933	Serum 25-Hydroxyvitamin D levels	NADSYN1	L2	0.006	1.47E-04
rs28435470	Serum 25-Hydroxyvitamin D levels	POLE	L2	0.006	1.47E-04
rs721917	Pulmonary surfactant-associated protein D levels	SFTPD	L2	0.008	0.047045383
rs10887331	Pulmonary surfactant-associated protein D levels	SFTPD	L2	0.008	0.047045383
rs34743464	Serum levels of protein KDR	KDR	L2	0.048	0.047045383
rs2305948	Serum levels of protein KDR	KDR	L2	0.048	0.047045383
rs199515	PD	WNT3	L3	0.044	0.040268727
rs199533	PD	WNT3	L3	0.044	0.040268727
rs11150601	PD	SETD1A	L3	0.044	0.040268727
rs140820592	PD	SETD1A	L3	0.044	0.040268727
rs415430	PD	WNT3	L3	0.044	0.040268727
rs199498	PD	WNT3	L3	0.044	0.040268727

rs17649553	PD	WNT3	L3	0.044	0.040268727
rs17577094	PD	AP2B1	L3	0.044	0.040268727
rs199501	PD	WNT3	L3	0.044	0.040268727
rs11078917	Smoking initiation	ERBB2	L3	0.026	0.015119503
rs2403254	Alpha-hydroxyisovalerate levels	GTF2H1	L3	0.026	6.54E-04
rs73058498	X-11315 levels	LIMD1	L3	0.032	6.56E-08
rs11716779	Smoking initiation	CDC25A	L3	0.026	0.015119503
rs455650	Smoking initiation	REV3L	L3	0.026	0.015119503
rs4945881	Smoking initiation	REV3L	L3	0.026	0.015119503
rs10899498	Smoking initiation	GAB2	L3	0.026	0.015119503
rs1970506	Smoking initiation	TNKS2	L3	0.026	0.015119503
rs13260666	Smoking initiation	PTK2	L3	0.026	0.015119503
rs17638867	Smoking initiation	AP2B1	L3	0.026	0.015119503
rs28628339	Smoking initiation	CDH3	L3	0.026	0.015119503
rs36061954	Smoking initiation	FGFR1	L3	0.026	0.015119503
rs4757638	Smoking initiation	GTF2H1	L3	0.026	0.015119503
rs2932966	Smoking initiation	PPARGC1A	L3	0.026	0.015119503
rs9469913	Smoking initiation	SNRPC	L3	0.026	0.015119503
rs5873246	Smoking initiation	DCC	L3	0.026	0.015119503
rs240977	Smoking initiation	REV3L	L3	0.026	0.015119503
rs240966	Smoking initiation	REV3L	L3	0.026	0.015119503
rs56298245	Smoking initiation	REV3L	L3	0.026	0.015119503
rs461646	Smoking initiation	REV3L	L3	0.026	0.015119503
rs6920014	Smoking initiation	REV3L	L3	0.026	0.015119503
rs150138167	Smoking initiation	REV3L	L3	0.026	0.015119503
rs9374263	Smoking initiation	REV3L	L3	0.026	0.015119503
rs7242289	Smoking initiation	TXNL4A	L3	0.026	0.015119503

rs11784581	Smoking initiation	PTK2	L3	0.026	0.015119503
rs113927489	Smoking initiation	RAPGEF1	L3	0.026	0.015119503
rs2421628	Smoking initiation	TNKS2	L3	0.026	0.015119503
rs12774295	Smoking initiation	TNKS2	L3	0.026	0.015119503
rs2676822	Smoking initiation	TNKS2	L3	0.026	0.015119503
rs2676820	Smoking initiation	TNKS2	L3	0.026	0.015119503
rs833384	Smoking initiation	TNKS2	L3	0.026	0.015119503
rs11237456	Smoking initiation	GAB2	L3	0.026	0.015119503
rs4945275	Smoking initiation	GAB2	L3	0.026	0.015119503
rs12225912	Smoking initiation	GAB2	L3	0.026	0.015119503
rs56102101	Smoking initiation	NUP54	L3	0.026	0.015119503
rs7722095	Smoking initiation	XRCC4	L3	0.026	0.015119503
rs12453682	Smoking initiation	ERBB2	L3	0.026	0.015119503
rs199510	Smoking initiation	WNT3	L3	0.026	0.015119503
rs7755822	Smoking initiation	SNRPC	L3	0.026	0.015119503
rs466765	Smoking initiation	REV3L	L3	0.026	0.015119503
rs399219	Smoking initiation	REV3L	L3	0.026	0.015119503
rs455645	Smoking initiation	REV3L	L3	0.026	0.015119503
rs462779	Smoking initiation	REV3L	L3	0.026	0.015119503
rs9487626	Smoking initiation	REV3L	L3	0.026	0.015119503
rs9487627	Smoking initiation	REV3L	L3	0.026	0.015119503
rs9487632	Smoking initiation	REV3L	L3	0.026	0.015119503
rs10214442	Smoking initiation	REV3L	L3	0.026	0.015119503
rs9487645	Smoking initiation	REV3L	L3	0.026	0.015119503
rs13060705	Smoking initiation	RAF1	L3	0.026	0.015119503
rs3802967	2-hydroxy-3-methylvalerate levels	GTF2H1	L3	0.026	2.52E-04
rs3802967	Alpha-hydroxyisovalerate levels	GTF2H1	L3	0.026	6.54E-04

rs12294486	2-hydroxy-3-methylvalerate levels	GTF2H1	L3	0.026	2.52E-04
rs11024613	2-hydroxy-3-methylvalerate levels	GTF2H1	L3	0.026	2.52E-04
rs10766469	2-hydroxy-3-methylvalerate levels	GTF2H1	L3	0.026	2.52E-04
rs17279437	X-11315 levels	LIMD1	L3	0.032	6.56E-08
rs17260680	X-11315 levels	LIMD1	L3	0.032	6.56E-08
rs4327428	X-11315 levels	LIMD1	L3	0.032	6.56E-08
rs3848666	X-11315 levels	NPHS1	L3	0.032	6.56E-08
rs3051635	X-11315 levels	LIMD1	L3	0.032	6.56E-08
rs10832919	Alpha-hydroxyisovalerate levels	GTF2H1	L3	0.026	6.54E-04
rs4596	Alpha-hydroxyisovalerate levels	GTF2H1	L3	0.026	6.54E-04

Supplementary Table 4. Smoking initiation associated SNPs and their target genes within the PD-causal network

SNP	trait	Target gene	PPI level	Bootstrap_pval	Bonferroni_adj_pval	eQTL beta
rs11078917	Smoking initiation	ERBB2	L3	0.026	0.015119503	-0.091085985
rs11716779	Smoking initiation	CDC25A	L3	0.026	0.015119503	0.35077527
rs455650	Smoking initiation	REV3L	L3	0.026	0.015119503	-0.30721644
rs4945881	Smoking initiation	REV3L	L3	0.026	0.015119503	0.3128581
rs10899498	Smoking initiation	GAB2	L3	0.026	0.015119503	-0.14513853
rs1970506	Smoking initiation	TNKS2	L3	0.026	0.015119503	NA
rs13260666	Smoking initiation	PTK2	L3	0.026	0.015119503	0.2047183
rs17638867	Smoking initiation	AP2B1	L3	0.026	0.015119503	0.14472711
rs28628339	Smoking initiation	CDH3	L3	0.026	0.015119503	0.23624544
rs36061954	Smoking initiation	FGFR1	L3	0.026	0.015119503	NA
rs4757638	Smoking initiation	GTF2H1	L3	0.026	0.015119503	NA
rs2932966	Smoking initiation	PPARGC1A	L3	0.026	0.015119503	NA

rs9469913	Smoking initiation	SNRPC	L3	0.026	0.015119503	NA
rs5873246	Smoking initiation	DCC	L3	0.026	0.015119503	NA
rs240977	Smoking initiation	REV3L	L3	0.026	0.015119503	-0.23443325
rs240966	Smoking initiation	REV3L	L3	0.026	0.015119503	-0.23770687
rs56298245	Smoking initiation	REV3L	L3	0.026	0.015119503	-0.31294715
rs461646	Smoking initiation	REV3L	L3	0.026	0.015119503	-0.30254546
rs6920014	Smoking initiation	REV3L	L3	0.026	0.015119503	-0.3129472
rs150138167	Smoking initiation	REV3L	L3	0.026	0.015119503	-0.30254546
rs9374263	Smoking initiation	REV3L	L3	0.026	0.015119503	0.23880339
rs7242289	Smoking initiation	TXNL4A	L3	0.026	0.015119503	NA
rs11784581	Smoking initiation	PTK2	L3	0.026	0.015119503	0.23658305
rs113927489	Smoking initiation	RAPGEF1	L3	0.026	0.015119503	NA
rs2421628	Smoking initiation	TNKS2	L3	0.026	0.015119503	NA
rs12774295	Smoking initiation	TNKS2	L3	0.026	0.015119503	-0.103184916
rs2676822	Smoking initiation	TNKS2	L3	0.026	0.015119503	NA
rs2676820	Smoking initiation	TNKS2	L3	0.026	0.015119503	NA
rs833384	Smoking initiation	TNKS2	L3	0.026	0.015119503	-0.103184916
rs11237456	Smoking initiation	GAB2	L3	0.026	0.015119503	-0.16979568
rs4945275	Smoking initiation	GAB2	L3	0.026	0.015119503	-0.15105952
rs12225912	Smoking initiation	GAB2	L3	0.026	0.015119503	NA
rs56102101	Smoking initiation	NUP54	L3	0.026	0.015119503	-0.22600879
rs7722095	Smoking initiation	XRCC4	L3	0.026	0.015119503	-0.18909717
rs12453682	Smoking initiation	ERBB2	L3	0.026	0.015119503	-0.07097175
rs199510	Smoking initiation	WNT3	L3	0.026	0.015119503	-0.32743636
rs7755822	Smoking initiation	SNRPC	L3	0.026	0.015119503	NA

rs466765	Smoking initiation	REV3L	L3	0.026	0.015119503	NA
rs399219	Smoking initiation	REV3L	L3	0.026	0.015119503	-0.31294718
rs455645	Smoking initiation	REV3L	L3	0.026	0.015119503	-0.30254546
rs462779	Smoking initiation	REV3L	L3	0.026	0.015119503	-0.30254546
rs9487626	Smoking initiation	REV3L	L3	0.026	0.015119503	-0.30254546
rs9487627	Smoking initiation	REV3L	L3	0.026	0.015119503	-0.30254546
rs9487632	Smoking initiation	REV3L	L3	0.026	0.015119503	-0.30254546
rs10214442	Smoking initiation	REV3L	L3	0.026	0.015119503	-0.30254546
rs9487645	Smoking initiation	REV3L	L3	0.026	0.015119503	-0.33361846
rs13060705	Smoking initiation	RAF1	L3	0.026	0.015119503	0.23499091

Supplementary Table 5. Traits enriched for loci regulating genes in PD-associated network.

SNP	trait	Target gene	PPI level	Bootstrap_pval	Bonferroni_adj_pval
rs6812193	PD	FAM47E	L0	0.022	1.69E-71
rs6812193	PD	CCDC158	L0	0.022	1.69E-71
rs12185268	PD	LINC02210	L0	0.022	1.69E-71
rs12185268	PD	KANSL1	L0	0.022	1.69E-71
rs4698412	PD	CD38	L0	0.022	1.69E-71
rs4698412	PD or first degree relation to individual with PD	CD38	L0	0.002	6.11E-74
rs11248060	PD	PCGF3	L0	0.022	1.69E-71
rs1296028	PD	CTSB	L0	0.022	1.69E-71
rs1536076	PD	SH3GL2	L0	0.022	1.69E-71
rs199515	PD	WNT3	L0	0.022	1.69E-71
rs199515	PD	KANSL1	L0	0.022	1.69E-71
rs7077361	PD	ITGA8	L0	0.022	1.69E-71

rs199533	PD	KANSL1	L0	0.022	1.69E-71
rs199533	PD	WNT3	L0	0.022	1.69E-71
rs393152	PD	KANSL1	L0	0.022	1.69E-71
rs393152	PD	LINC02210	L0	0.022	1.69E-71
rs4538475	PD	CD38	L0	0.022	1.69E-71
rs947211	PD	RAB29	L0	0.022	1.69E-71
rs6599388	PD	PCGF3	L0	0.022	1.69E-71
rs11724635	PD	CD38	L0	0.022	1.69E-71
rs1491942	PD	LRRK2	L0	0.022	1.69E-71
rs2942168	PD	KANSL1	L0	0.022	1.69E-71
rs2942168	PD	LINC02210	L0	0.022	1.69E-71
rs11012	PD	LINC02210	L0	0.022	1.69E-71
rs113434679	PD	KANSL1	L0	0.022	1.69E-71
rs113434679	PD	LINC02210	L0	0.022	1.69E-71
rs113434679	General factor of neuroticism	KANSL1	L0	0.026	4.08E-10
rs113434679	General factor of neuroticism	LINC02210	L0	0.026	4.08E-10
rs9275152	PD	HLA-DRB1	L0	0.022	1.69E-71
rs62053943	PD	LINC02210	L0	0.022	1.69E-71
rs62053943	PD or first degree relation to individual with PD	LINC02210	L0	0.002	6.11E-74
rs62053943	White matter microstructure (radial diusivities)	LINC02210	L0	0.016	5.34E-04
rs62053943	General factor of neuroticism	LINC02210	L0	0.026	4.08E-10
rs34311866	PD	PCGF3	L0	0.022	1.69E-71
rs34311866	PD or first degree relation to individual with PD	PCGF3	L0	0.002	6.11E-74
rs11150601	PD	SETD1A	L0	0.022	1.69E-71
rs11150601	PD	STX4	L0	0.022	1.69E-71
rs11150601	PD	PRSS36	L0	0.022	1.69E-71
rs11150601	PD or first degree relation to individual with PD	SETD1A	L0	0.002	6.11E-74

rs11150601	PD or first degree relation to individual with PD	STX4	L0	0.002	6.11E-74
rs11150601	PD or first degree relation to individual with PD	PRSS36	L0	0.002	6.11E-74
rs28624974	PD	GPNMB	L0	0.022	1.69E-71
rs28624974	PD	NUPL2	L0	0.022	1.69E-71
rs1293298	PD	CTSB	L0	0.022	1.69E-71
rs1293298	PD or first degree relation to individual with PD	CTSB	L0	0.002	6.11E-74
rs1450522	PD	SPTSSB	L0	0.022	1.69E-71
rs1450522	PD or first degree relation to individual with PD	SPTSSB	L0	0.002	6.11E-74
rs12643261	PD	FAM47E	L0	0.022	1.69E-71
rs113564729	PD	KANSL1	L0	0.022	1.69E-71
rs2647062	PD	HLA-DRB1	L0	0.022	1.69E-71
rs10797576	PD	PTPRG	L0	0.022	1.69E-71
rs10797576	PD or first degree relation to individual with PD	PTPRG	L0	0.002	6.11E-74
rs10906923	PD	ITGA8	L0	0.022	1.69E-71
rs140820592	PD	SETD1A	L0	0.022	1.69E-71
rs140820592	PD	STX4	L0	0.022	1.69E-71
rs1867598	PD	NDUFAF2	L0	0.022	1.69E-71
rs1867598	PD or first degree relation to individual with PD	NDUFAF2	L0	0.002	6.11E-74
rs8087969	PD	MEX3C	L0	0.022	1.69E-71
rs8087969	PD or first degree relation to individual with PD	MEX3C	L0	0.002	6.11E-74
rs6449168	PD	CD38	L0	0.022	1.69E-71
rs415430	PD	WNT3	L0	0.022	1.69E-71
rs415430	PD	KANSL1	L0	0.022	1.69E-71
rs199498	PD	WNT3	L0	0.022	1.69E-71
rs199498	PD	KANSL1	L0	0.022	1.69E-71
rs17649553	PD	KANSL1	L0	0.022	1.69E-71
rs17649553	PD	WNT3	L0	0.022	1.69E-71

rs62120679	PD	LSM7	L0	0.022	1.69E-71
rs823118	PD	NUCKS1	L0	0.022	1.69E-71
rs823118	PD or first degree relation to individual with PD	NUCKS1	L0	0.002	6.11E-74
rs199347	PD	GPNMB	L0	0.022	1.69E-71
rs199347	PD	NUPL2	L0	0.022	1.69E-71
rs76904798	PD	LRRK2	L0	0.022	1.69E-71
rs76904798	PD or first degree relation to individual with PD	LRRK2	L0	0.002	6.11E-74
rs17577094	PD	KANSL1	L0	0.022	1.69E-71
rs823114	PD	NUCKS1	L0	0.022	1.69E-71
rs10463554	PD	PPIP5K2	L0	0.022	1.69E-71
rs9568188	PD	CAB39L	L0	0.022	1.69E-71
rs9568188	PD or first degree relation to individual with PD	CAB39L	L0	0.002	6.11E-74
rs12497850	PD	IP6K2	L0	0.022	1.69E-71
rs12497850	PD	ARIH2	L0	0.022	1.69E-71
rs12497850	PD	P4HTM	L0	0.022	1.69E-71
rs12497850	PD or first degree relation to individual with PD	IP6K2	L0	0.002	6.11E-74
rs12497850	PD or first degree relation to individual with PD	ARIH2	L0	0.002	6.11E-74
rs12497850	PD or first degree relation to individual with PD	P4HTM	L0	0.002	6.11E-74
rs2280104	PD	BIN3	L0	0.022	1.69E-71
rs2280104	PD or first degree relation to individual with PD	BIN3	L0	0.002	6.11E-74
rs67460515	PD	SPTSSB	L0	0.022	1.69E-71
rs2694528	PD	NDUFAF2	L0	0.022	1.69E-71
rs2740594	PD	CTSB	L0	0.022	1.69E-71
rs7221167	PD	LINC02210	L0	0.022	1.69E-71
rs7221167	PD or first degree relation to individual with PD	LINC02210	L0	0.002	6.11E-74
rs7221167	General factor of neuroticism	LINC02210	L0	0.026	4.08E-10
rs199501	PD	WNT3	L0	0.022	1.69E-71

rs199501	PD	KANSL1	L0	0.022	1.69E-71
rs4266290	PD	CD38	L0	0.022	1.69E-71
rs2209440	PD	SH3GL2	L0	0.022	1.69E-71
rs162227	PD	NDUFAF2	L0	0.022	1.69E-71
rs365825	PD	KANSL1	L0	0.022	1.69E-71
rs365825	PD	LINC02210	L0	0.022	1.69E-71
rs111433752	Neuroticism	LINC02210	L0	0.032	1.21E-09
rs1045430	Neuroticism	AREL1	L0	0.032	1.21E-09
rs1230106	Neuroticism	LINC02210	L0	0.032	1.21E-09
rs1724422	Neuroticism	LINC02210	L0	0.032	1.21E-09
rs1724422	Depressed affect	LINC02210	L0	0.01	6.85E-06
rs61572747	Neuroticism	KANSL1	L0	0.032	1.21E-09
rs61572747	Depressed affect	KANSL1	L0	0.01	6.85E-06
rs61572747	White matter microstructure (mean diusivities)	KANSL1	L0	0.008	6.19E-05
rs61572747	White matter microstructure (radial diusivities)	KANSL1	L0	0.016	5.34E-04
rs61572747	General factor of neuroticism	KANSL1	L0	0.026	4.08E-10
rs113100008	Neuroticism	LINC02210	L0	0.032	1.21E-09
rs35909029	Neuroticism	LINC02210	L0	0.032	1.21E-09
rs35909029	Depressed affect	LINC02210	L0	0.01	6.85E-06
rs34186148	Neuroticism	LINC02210	L0	0.032	1.21E-09
rs34186148	Depressed affect	LINC02210	L0	0.01	6.85E-06
rs34186148	White matter microstructure (mean diusivities)	LINC02210	L0	0.008	6.19E-05
rs34186148	White matter microstructure (radial diusivities)	LINC02210	L0	0.016	5.34E-04
rs4076453	Neuroticism	LINC02210	L0	0.032	1.21E-09
rs3785879	Neuroticism	KANSL1	L0	0.032	1.21E-09
rs3785879	Worry	KANSL1	L0	0.01	3.63E-06
rs2435207	Neuroticism	KANSL1	L0	0.032	1.21E-09

rs2016034	Neuroticism	KANSL1	L0	0.032	1.21E-09
rs199447	Neuroticism	KANSL1	L0	0.032	1.21E-09
rs199447	Neuroticism	WNT3	L0	0.032	1.21E-09
rs77804065	Neuroticism	LINC02210	L0	0.032	1.21E-09
rs77804065	Neuroticism	KANSL1	L0	0.032	1.21E-09
rs77804065	Feeling guilty	LINC02210	L0	0.01	2.82E-04
rs77804065	Feeling guilty	KANSL1	L0	0.01	2.82E-04
rs2165197	Neuroticism	AREL1	L0	0.032	1.21E-09
rs7225384	Neuroticism	LINC02210	L0	0.032	1.21E-09
rs7225384	Depressed affect	LINC02210	L0	0.01	6.85E-06
rs242947	Neuroticism	LINC02210	L0	0.032	1.21E-09
rs242947	Depressed affect	LINC02210	L0	0.01	6.85E-06
rs242947	General factor of neuroticism	LINC02210	L0	0.026	4.08E-10
rs754593	Neuroticism	LINC02210	L0	0.032	1.21E-09
rs754593	Depressed affect	LINC02210	L0	0.01	6.85E-06
rs754593	General factor of neuroticism	LINC02210	L0	0.026	4.08E-10
rs1563304	Neuroticism	WNT3	L0	0.032	1.21E-09
rs1563304	Experiencing mood swings	WNT3	L0	0.014	5.11E-05
rs1563304	Feeling hurt	WNT3	L0	0.012	0.003040444
rs7144654	Neuroticism	AREL1	L0	0.032	1.21E-09
rs2732708	Neuroticism	KANSL1	L0	0.032	1.21E-09
rs2732708	Neuroticism	LINC02210	L0	0.032	1.21E-09
rs17652520	Neuroticism	KANSL1	L0	0.032	1.21E-09
rs17652520	Household income	KANSL1	L0	0.012	0.024373056
rs2214258	Neuroticism	KANSL1	L0	0.032	1.21E-09
rs2214258	Neuroticism	LINC02210	L0	0.032	1.21E-09
rs35641442	Neuroticism	AREL1	L0	0.032	1.21E-09

rs12587457	Neuroticism	AREL1	L0	0.032	1.21E-09
rs62062288	Neuroticism	KANSL1	L0	0.032	1.21E-09
rs62062288	Feeling worry	KANSL1	L0	0.006	6.24E-05
rs62062288	Feeling guilty	KANSL1	L0	0.01	2.82E-04
rs12944712	Neuroticism	LINC02210	L0	0.032	1.21E-09
rs55955207	Neuroticism	KANSL1	L0	0.032	1.21E-09
rs55955207	Neuroticism	LINC02210	L0	0.032	1.21E-09
rs62057061	Depressed affect	LINC02210	L0	0.01	6.85E-06
rs12938031	Depressed affect	LINC02210	L0	0.01	6.85E-06
rs12938031	Worry	LINC02210	L0	0.01	3.63E-06
rs12938031	General factor of neuroticism	LINC02210	L0	0.026	4.08E-10
rs199505	Depressed affect	WNT3	L0	0.01	6.85E-06
rs199505	Depressed affect	KANSL1	L0	0.01	6.85E-06
rs6825004	PD or first degree relation to individual with PD	FAM47E	L0	0.002	6.11E-74
rs11578699	PD or first degree relation to individual with PD	METTL13	L0	0.002	6.11E-74
rs11557080	PD or first degree relation to individual with PD	RAB29	L0	0.002	6.11E-74
rs2042477	PD or first degree relation to individual with PD	KCNIP3	L0	0.002	6.11E-74
rs9261484	PD or first degree relation to individual with PD	ZNRD1ASP	L0	0.002	6.11E-74
rs112485576	PD or first degree relation to individual with PD	HLA-DRB1	L0	0.002	6.11E-74
rs55961674	PD or first degree relation to individual with PD	CCDC58	L0	0.002	6.11E-74
rs199351	PD or first degree relation to individual with PD	GPNMB	L0	0.002	6.11E-74
rs199351	PD or first degree relation to individual with PD	NUPL2	L0	0.002	6.11E-74
rs2086641	PD or first degree relation to individual with PD	FAM49B	L0	0.002	6.11E-74
rs6854006	PD or first degree relation to individual with PD	FAM47E	L0	0.002	6.11E-74
rs6854006	PD or first degree relation to individual with PD	CCDC158	L0	0.002	6.11E-74
rs5019538	PD or first degree relation to individual with PD	ALPK1	L0	0.002	6.11E-74
rs26431	PD or first degree relation to individual with PD	PPIP5K2	L0	0.002	6.11E-74

rs26431	PD or first degree relation to individual with PD	ST8SIA4	L0	0.002	6.11E-74
rs26431	Feeling worry	PPIP5K2	L0	0.006	6.24E-05
rs26431	Feeling worry	ST8SIA4	L0	0.006	6.24E-05
rs11707416	PD or first degree relation to individual with PD	CLRN1-AS1	L0	0.002	6.11E-74
rs76949143	PD or first degree relation to individual with PD	GS1-124K5.12	L0	0.002	6.11E-74
rs10756907	PD or first degree relation to individual with PD	SH3GL2	L0	0.002	6.11E-74
rs6476434	PD or first degree relation to individual with PD	PRSS3	L0	0.002	6.11E-74
rs6476434	PD or first degree relation to individual with PD	UBAP2	L0	0.002	6.11E-74
rs12951632	PD or first degree relation to individual with PD	FAM134C	L0	0.002	6.11E-74
rs12951632	PD or first degree relation to individual with PD	SKAP1	L0	0.002	6.11E-74
rs117615688	PD or first degree relation to individual with PD	LINC02210	L0	0.002	6.11E-74
rs11658976	PD or first degree relation to individual with PD	KANSL1	L0	0.002	6.11E-74
rs11658976	PD or first degree relation to individual with PD	RP11-156P1.3	L0	0.002	6.11E-74
rs11658976	PD or first degree relation to individual with PD	WNT3	L0	0.002	6.11E-74
rs666463	PD or first degree relation to individual with PD	PGS1	L0	0.002	6.11E-74
rs666463	PD or first degree relation to individual with PD	TK1	L0	0.002	6.11E-74
rs55818311	PD or first degree relation to individual with PD	LSM7	L0	0.002	6.11E-74
rs2248244	PD or first degree relation to individual with PD	DYRK1A	L0	0.002	6.11E-74
rs896435	PD or first degree relation to individual with PD	ITGA8	L0	0.002	6.11E-74
rs896435	PD or first degree relation to individual with PD	PRKG1	L0	0.002	6.11E-74
rs7938782	PD or first degree relation to individual with PD	AMPD3	L0	0.002	6.11E-74
rs7938782	PD or first degree relation to individual with PD	MRVI1-AS1	L0	0.002	6.11E-74
rs7938782	PD or first degree relation to individual with PD	RNF141	L0	0.002	6.11E-74
rs12283611	PD or first degree relation to individual with PD	ARHGEF12	L0	0.002	6.11E-74
rs11610045	PD or first degree relation to individual with PD	POLE	L0	0.002	6.11E-74
rs3742785	PD or first degree relation to individual with PD	AREL1	L0	0.002	6.11E-74
rs2904880	PD or first degree relation to individual with PD	NFATC2IP	L0	0.002	6.11E-74

rs6500328	PD or first degree relation to individual with PD	CHD9	L0	0.002	6.11E-74
rs6500328	PD or first degree relation to individual with PD	NOD2	L0	0.002	6.11E-74
rs3104783	PD or first degree relation to individual with PD	TOX3	L0	0.002	6.11E-74
rs199453	PD or first degree relation to individual with PD	WNT3	L0	0.002	6.11E-74
rs199453	PD or first degree relation to individual with PD	KANSL1	L0	0.002	6.11E-74
rs11159097	Worry	AREL1	L0	0.01	3.63E-06
rs7152906	Worry	AREL1	L0	0.01	3.63E-06
rs7152906	Feeling worry	AREL1	L0	0.006	6.24E-05
rs2435210	Worry	KANSL1	L0	0.01	3.63E-06
rs28426374	Worry	AREL1	L0	0.01	3.63E-06
rs59735493	Worry	SETD1A	L0	0.01	3.63E-06
rs62064641	Worry	LINC02210	L0	0.01	3.63E-06
rs62064641	Worry	KANSL1	L0	0.01	3.63E-06
rs7220839	Worry	LINC02210	L0	0.01	3.63E-06
rs9896243	Worry	KANSL1	L0	0.01	3.63E-06
rs9896243	Worry	WNT3	L0	0.01	3.63E-06
rs241036	Experiencing mood swings	KANSL1	L0	0.014	5.11E-05
rs241036	Experiencing mood swings	LINC02210	L0	0.014	5.11E-05
rs55657917	Experiencing mood swings	KANSL1	L0	0.014	5.11E-05
rs55657917	Experiencing mood swings	LINC02210	L0	0.014	5.11E-05
rs55657917	Feeling hurt	KANSL1	L0	0.012	0.003040444
rs55657917	Feeling hurt	LINC02210	L0	0.012	0.003040444
rs79857651	Experiencing mood swings	KANSL1	L0	0.014	5.11E-05
rs112333322	Experiencing mood swings	KANSL1	L0	0.014	5.11E-05
rs112333322	Experiencing mood swings	LINC02210	L0	0.014	5.11E-05
rs17665188	Experiencing mood swings	KANSL1	L0	0.014	5.11E-05
rs7895261	Experiencing mood swings	PRKG1	L0	0.014	5.11E-05

rs62055701	Irritable mood	LINC02210	L0	0.022	4.15E-05
rs62055701	Irritable mood	KANSL1	L0	0.022	4.15E-05
rs2106785	Irritable mood	LINC02210	L0	0.022	4.15E-05
rs2106785	Irritable mood	KANSL1	L0	0.022	4.15E-05
rs17650842	Irritable mood	KANSL1	L0	0.022	4.15E-05
rs17661015	Irritable mood	KANSL1	L0	0.022	4.15E-05
rs17661015	Irritable mood	LINC02210	L0	0.022	4.15E-05
rs17661015	Feeling hurt	KANSL1	L0	0.012	0.003040444
rs17661015	Feeling hurt	LINC02210	L0	0.012	0.003040444
rs2261201	Irritable mood	KANSL1	L0	0.022	4.15E-05
rs70600	Irritable mood	WNT3	L0	0.022	4.15E-05
rs70600	Irritable mood	KANSL1	L0	0.022	4.15E-05
rs4499638	Irritable mood	P4HTM	L0	0.022	4.15E-05
rs187580	Feeling worry	PPIP5K2	L0	0.006	6.24E-05
rs17688916	Feeling worry	LINC02210	L0	0.006	6.24E-05
rs17688916	Feeling worry	KANSL1	L0	0.006	6.24E-05
rs3759741	Feeling worry	AREL1	L0	0.006	6.24E-05
rs62057151	Feeling worry	KANSL1	L0	0.006	6.24E-05
rs62061733	Feeling hurt	KANSL1	L0	0.012	0.003040444
rs2696668	Feeling hurt	KANSL1	L0	0.012	0.003040444
rs2696668	Feeling hurt	LINC02210	L0	0.012	0.003040444
rs4471723	Feeling guilty	KANSL1	L0	0.01	2.82E-04
rs4471723	Feeling guilty	WNT3	L0	0.01	2.82E-04
rs2696532	Feeling guilty	KANSL1	L0	0.01	2.82E-04
rs2696532	Feeling guilty	LINC02210	L0	0.01	2.82E-04
rs199525	Feeling guilty	KANSL1	L0	0.01	2.82E-04
rs199525	Feeling guilty	WNT3	L0	0.01	2.82E-04

rs2272767	Cathepsin B levels	CTSB	L0	0.01	2.69E-06
rs1692812	Cathepsin B levels	CTSB	L0	0.01	2.69E-06
rs3947	Cathepsin B levels	CTSB	L0	0.01	2.69E-06
rs1692813	Cathepsin B levels	CTSB	L0	0.01	2.69E-06
rs28383228	Ubiquitin carboxyl-terminal hydrolase 25 levels (USP25.9215.117.3)	HLA-DRB1	L0	0.016	0.048348116
rs9270891	Ubiquitin carboxyl-terminal hydrolase 25 levels (USP25.9215.117.3)	HLA-DRB1	L0	0.016	0.048348116
rs28746950	Ubiquitin carboxyl-terminal hydrolase 25 levels (USP25.9215.117.3)	HLA-DRB1	L0	0.016	0.048348116
rs35519908	White matter microstructure (mean diusivities)	LINC02210	L0	0.008	6.19E-05
rs55938136	White matter microstructure (mean diusivities)	LINC02210	L0	0.008	6.19E-05
rs55938136	White matter microstructure (axial diusivities)	LINC02210	L0	0.008	9.04E-11
rs55938136	White matter microstructure (radial diusivities)	LINC02210	L0	0.016	5.34E-04
rs2696466	White matter microstructure (mean diusivities)	LINC02210	L0	0.008	6.19E-05
rs2696466	White matter microstructure (radial diusivities)	LINC02210	L0	0.016	5.34E-04
rs34081316	White matter microstructure (mean diusivities)	KANSL1	L0	0.008	6.19E-05
rs34081316	White matter microstructure (axial diusivities)	KANSL1	L0	0.008	9.04E-11
rs34081316	White matter microstructure (radial diusivities)	KANSL1	L0	0.016	5.34E-04
rs3890609	White matter microstructure (mean diusivities)	KANSL1	L0	0.008	6.19E-05
rs3890609	White matter microstructure (mean diusivities)	LINC02210	L0	0.008	6.19E-05
rs3890609	White matter microstructure (axial diusivities)	KANSL1	L0	0.008	9.04E-11
rs3890609	White matter microstructure (axial diusivities)	LINC02210	L0	0.008	9.04E-11
rs3890609	White matter microstructure (radial diusivities)	KANSL1	L0	0.016	5.34E-04
rs3890609	White matter microstructure (radial diusivities)	LINC02210	L0	0.016	5.34E-04
rs368919625	White matter microstructure (mean diusivities)	KANSL1	L0	0.008	6.19E-05
rs10840422	White matter microstructure (mean diusivities)	AMPD3	L0	0.008	6.19E-05

rs369762169	White matter microstructure (mean diusivities)	LINC02210	L0	0.008	6.19E-05
rs1450270	White matter microstructure (axial diusivities)	AMPD3	L0	0.008	9.04E-11
rs7119498	White matter microstructure (axial diusivities)	AMPD3	L0	0.008	9.04E-11
rs899010	White matter microstructure (axial diusivities)	AMPD3	L0	0.008	9.04E-11
rs930672	White matter microstructure (axial diusivities)	AMPD3	L0	0.008	9.04E-11
rs936513	White matter microstructure (axial diusivities)	AMPD3	L0	0.008	9.04E-11
rs564681262	White matter microstructure (axial diusivities)	LINC02210	L0	0.008	9.04E-11
rs7944154	White matter microstructure (axial diusivities)	AMPD3	L0	0.008	9.04E-11
rs10770119	White matter microstructure (axial diusivities)	AMPD3	L0	0.008	9.04E-11
rs11042811	White matter microstructure (axial diusivities)	AMPD3	L0	0.008	9.04E-11
rs550530305	White matter microstructure (axial diusivities)	KANSL1	L0	0.008	9.04E-11
rs35862435	White matter microstructure (axial diusivities)	AMPD3	L0	0.008	9.04E-11
rs147431626	White matter microstructure (radial diusivities)	KANSL1	L0	0.016	5.34E-04
rs548785130	White matter microstructure (radial diusivities)	KANSL1	L0	0.016	5.34E-04
rs577386308	White matter microstructure (radial diusivities)	KANSL1	L0	0.016	5.34E-04
rs2280406	Household income	IP6K2	L0	0.012	0.024373056
rs57468227	Household income	NDUFAF2	L0	0.012	0.024373056
rs62056789	Household income	KANSL1	L0	0.012	0.024373056
rs974295	Household income	KANSL1	L0	0.012	0.024373056
rs974295	Household income	LINC02210	L0	0.012	0.024373056
rs2409836	General factor of neuroticism	CTSB	L0	0.026	4.08E-10
rs2645430	General factor of neuroticism	CTSB	L0	0.026	4.08E-10
rs2074404	General factor of neuroticism	WNT3	L0	0.026	4.08E-10
rs1724425	General factor of neuroticism	LINC02210	L0	0.026	4.08E-10
rs199497	General factor of neuroticism	WNT3	L0	0.026	4.08E-10
rs3843738	General factor of neuroticism	LINC02210	L0	0.026	4.08E-10
rs35076622	General factor of neuroticism	LINC02210	L0	0.026	4.08E-10

rs12889665	General factor of neuroticism	AREL1	L0	0.026	4.08E-10
rs16661	General factor of neuroticism	AREL1	L0	0.026	4.08E-10
rs199529	General factor of neuroticism	WNT3	L0	0.026	4.08E-10
rs3104413	ACPA-positive rheumatoid arthritis (smoking interaction)	HLA-DRB1	L0	0.008	0.048348116
rs6931277	ACPA-positive rheumatoid arthritis (smoking interaction)	HLA-DRB1	L0	0.008	0.048348116
rs3129769	ACPA-positive rheumatoid arthritis (smoking interaction)	HLA-DRB1	L0	0.008	0.048348116
rs13101828	Alpha-L-iduronidase levels (IDUA.3169.70.2)	GAK	L1	0.02	4.93E-04
rs10876432	Bone mineral density (spine)	AAAS	L1	0.01	0.007023297
rs2016266	Bone mineral density (spine)	AAAS	L1	0.01	0.007023297
rs3130340	Bone mineral density (spine)	HLA-DMA	L1	0.01	0.007023297
rs2272313	Bone mineral density (spine)	AAAS	L1	0.01	0.007023297
rs10747666	Bone mineral density (spine)	AAAS	L1	0.01	0.007023297
rs6031847	14-3-3 protein beta/alpha levels	YWHAB	L1	0.02	1.42E-05
rs1884440	14-3-3 protein beta/alpha levels	YWHAB	L1	0.02	1.42E-05
rs6130713	14-3-3 protein beta/alpha levels	YWHAB	L1	0.02	1.42E-05
rs35797567	Ectonucleoside triphosphate diphosphohydrolase 5 levels	LIN52	L1	0.012	1.42E-09
rs73301493	Ectonucleoside triphosphate diphosphohydrolase 5 levels	LIN52	L1	0.012	1.42E-09
rs4903201	Ectonucleoside triphosphate diphosphohydrolase 5 levels	LIN52	L1	0.012	1.42E-09
rs62005072	Ectonucleoside triphosphate diphosphohydrolase 5 levels	LIN52	L1	0.012	1.42E-09
rs146086623	Ectonucleoside triphosphate diphosphohydrolase 5 levels	LIN52	L1	0.012	1.42E-09

rs7160336	Ectonucleoside triphosphate diphosphohydrolase 5 levels	LIN52	L1	0.012	1.42E-09
rs59892091	Ectonucleoside triphosphate diphosphohydrolase 5 levels	LIN52	L1	0.012	1.42E-09
rs57731447	Ectonucleoside triphosphate diphosphohydrolase 5 levels (ENTPD5.4437.56.3)	LIN52	L1	0.01	0.004943468
rs11332437	Ectonucleoside triphosphate diphosphohydrolase 5 levels (ENTPD5.4437.56.3)	LIN52	L1	0.01	0.004943468
rs4690203	Alpha-L-iduronidase levels (IDUA.3169.70.2)	GAK	L1	0.02	4.93E-04
rs56079856	Alpha-L-iduronidase levels (IDUA.3169.70.2)	GAK	L1	0.02	4.93E-04
rs12648219	Circulating odd-numbered chain saturated fatty acid levels (C23:0)	FRAS1	L2	0.008	2.22E-09
rs6839280	Circulating odd-numbered chain saturated fatty acid levels (C23:0)	FRAS1	L2	0.008	2.22E-09
rs6857679	Circulating odd-numbered chain saturated fatty acid levels (C23:0)	FRAS1	L2	0.008	2.22E-09
rs7439893	Circulating odd-numbered chain saturated fatty acid levels (C23:0)	FRAS1	L2	0.008	2.22E-09
rs727761	Circulating odd-numbered chain saturated fatty acid levels (C23:0)	FRAS1	L2	0.008	2.22E-09
rs10518201	Circulating odd-numbered chain saturated fatty acid levels (C23:0)	FRAS1	L2	0.008	2.22E-09
rs727760	Circulating odd-numbered chain saturated fatty acid levels (C23:0)	FRAS1	L2	0.008	2.22E-09
rs10793129	Serum 25-Hydroxyvitamin D levels	DGAT2	L2	0.014	0.017909002
rs200454003	Serum 25-Hydroxyvitamin D levels	NADSYN1	L2	0.014	0.017909002
rs7938885	Serum 25-Hydroxyvitamin D levels	NADSYN1	L2	0.014	0.017909002
rs12800438	Serum 25-Hydroxyvitamin D levels	NADSYN1	L2	0.014	0.017909002
rs12278461	Serum 25-Hydroxyvitamin D levels	NADSYN1	L2	0.014	0.017909002

rs2297991	Serum 25-Hydroxyvitamin D levels	VTI1A	L2	0.014	0.017909002
rs72997623	Serum 25-Hydroxyvitamin D levels	DGAT2	L2	0.014	0.017909002
rs1894100	Serum 25-Hydroxyvitamin D levels	NADSYN1	L2	0.014	0.017909002
rs1790373	Serum 25-Hydroxyvitamin D levels	NADSYN1	L2	0.014	0.017909002
rs11233933	Serum 25-Hydroxyvitamin D levels	NADSYN1	L2	0.014	0.017909002
rs3060	Serum 25-Hydroxyvitamin D levels	DGAT2	L2	0.014	0.017909002
rs62013200	Pro-cathepsin H levels	CTSH	L2	0.026	0.038875608
rs2289702	Pro-cathepsin H levels	CTSH	L2	0.026	0.038875608
rs2289702	Cathepsin H levels	CTSH	L2	0.026	0.038437046
rs111495139	Cathepsin H levels	CTSH	L2	0.026	0.038437046
rs62013199	Cathepsin H levels	CTSH	L2	0.026	0.038437046
rs11586493	Age at menopause	STX6	L2	0.014	0.039381396
rs11650324	Age at menopause	NUP88	L2	0.014	0.039381396
rs8077172	Age at menopause	ERBB2	L2	0.014	0.039381396
rs2277339	Age at menopause	PRIM1	L2	0.014	0.039381396
rs1760940	Age at menopause	PNP	L2	0.014	0.039381396
rs660158	Age at menopause	RAD18	L2	0.014	0.039381396
rs11767307	Age at menopause	AMPH	L2	0.014	0.039381396
rs141471965	3-bromo-5-chloro-2,6-dihydroxybenzoic acid levels	KCNIP4	L2	0.046	0.038875608
rs2231142	3-bromo-5-chloro-2,6-dihydroxybenzoic acid levels	KCNIP4	L2	0.046	0.038875608
rs6510827	Vitiligo	TICAM1	L3	0.024	0.009270444
rs4822024	Vitiligo	EP300	L3	0.024	0.009270444
rs3814231	Vitiligo	CASP7	L3	0.024	0.009270444
rs10986311	Vitiligo	PSMB7	L3	0.024	0.009270444
rs11079035	Vitiligo	RAB5C	L3	0.024	0.009270444
rs4807000	Vitiligo	TICAM1	L3	0.024	0.009270444
rs9611565	Vitiligo	EP300	L3	0.024	0.009270444

rs12771452	Vitiligo	CASP7	L3	0.024	0.009270444
rs174548	Triglycerides	FEN1	L3	0.026	0.01614934
rs1178979	Triglycerides	BAZ1B	L3	0.026	0.01614934
rs174546	Triglycerides	FEN1	L3	0.026	0.01614934
rs174547	Triglycerides	FEN1	L3	0.026	0.01614934
rs8077889	Triglycerides	LSM12	L3	0.026	0.01614934
rs1531140	Triglycerides	MGA	L3	0.026	0.01614934
rs174550	Triglycerides	FEN1	L3	0.026	0.01614934
rs174551	Triglycerides	FEN1	L3	0.026	0.01614934
rs174554	Triglycerides	FEN1	L3	0.026	0.01614934
rs174577	Triglycerides	FEN1	L3	0.026	0.01614934
rs7811265	Triglycerides	BAZ1B	L3	0.026	0.01614934
rs55931203	Triglycerides	BPTF	L3	0.026	0.01614934
rs1322842	Triglycerides	E2F3	L3	0.026	0.01614934
rs12945575	Triglycerides	RAB5C	L3	0.026	0.01614934
rs10793310	Triglycerides	GAB2	L3	0.026	0.01614934
rs9948087	Triglycerides	USP14	L3	0.026	0.01614934
rs12206516	Triglycerides	E2F3	L3	0.026	0.01614934
rs2971672	Triglycerides	YKT6	L3	0.026	0.01614934
rs7502307	Triglycerides	BPTF	L3	0.026	0.01614934
rs11745702	Triglycerides	HSPA4	L3	0.026	0.01614934
rs12928099	Triglycerides	RBFOX1	L3	0.026	0.01614934
rs1519480	Triglycerides	BDNF	L3	0.026	0.01614934
rs11868959	Triglycerides	BPTF	L3	0.026	0.01614934
rs174574	Triglycerides	FEN1	L3	0.026	0.01614934
rs202186505	Triglycerides	GAB2	L3	0.026	0.01614934

Supplementary Table 6. Biological pathways enriched for PD-causal network genes

Pathway description	genes	source	p_value	term_size	query_size	precision	recall
Disease	*ADORA2B,AAAS,CHMP4B,ATM,NUP88, NRG1,SFTPD,KDR,AP2B1,ERBB2,GTF2H1, CDC25A,GAB2,TNKS2,PTK2,FGFR1,NUP54, XRCC4,RAF1*	REAC	1.78E-06	1170	51	3.73E-01	1.62E-02
NAD+ biosynthetic pathways	*CD38,NADSYN1,SIRT3,TNKS2*	WP	5.46E-05	16	51	7.84E-02	2.50E-01
Malignant pleural mesothelioma	*ATM,KDR,WNT3,LIMD1,PTK2,CDH3,FGFR1, PPARGC1A,RAF1*	WP	1.74E-04	319	51	1.76E-01	2.82E-02
HIV Life Cycle	*AAAS,CHMP4B,NUP88,GTF2H1,NUP54, XRCC4*	REAC	2.32E-04	105	51	1.18E-01	5.71E-02
HIV Infection	*AAAS,CHMP4B,NUP88,AP2B1,GTF2H1, NUP54,XRCC4*	REAC	2.32E-04	160	51	1.37E-01	4.38E-02
Proteoglycans in cancer	*KDR,WNT3,ERBB2,PTK2,FGFR1,RAF1*	KEGG	0.0011	151	51	1.18E-01	3.97E-02
DNA repair pathways, full network	*ATM,POLE,GTF2H1,REV3L,XRCC4*	WP	0.0011	99	51	9.80E-02	5.05E-02
Breast cancer pathway	*ATM,WNT3,ERBB2,FGFR1,RAF1*	WP	0.0011	97	51	9.80E-02	5.15E-02
Pathways affected in adenoid cystic carcinoma	*KANSL1,ATM,ERBB2,RAF1*	WP	0.0011	51	51	7.84E-02	7.84E-02
Central carbon metabolism in cancer	*SIRT3,ERBB2,FGFR1,RAF1*	KEGG	0.0013	49	51	7.84E-02	8.16E-02
Prolactin signaling pathway	*ERBB2,GAB2,PTK2,RAF1*	WP	0.0013	55	51	7.84E-02	7.27E-02
HCMV Late Events	*AAAS,CHMP4B,NUP88,NUP54*	REAC	0.0016	43	51	7.84E-02	9.30E-02
Late Phase of HIV Life Cycle	*AAAS,CHMP4B,NUP88,GTF2H1,NUP54*	REAC	0.0020	97	51	9.80E-02	5.15E-02
Regulation of HSF1-mediated heat shock response	*AAAS,ATM,NUP88,NUP54*	REAC	0.0021	50	51	7.84E-02	8.00E-02
EGFR tyrosine kinase inhibitor resistance	*NRG1,KDR,ERBB2,RAF1*	WP	0.0021	68	51	7.84E-02	5.88E-02
Glioblastoma signaling pathways	*ATM,ERBB2,FGFR1,RAF1*	WP	0.0021	66	51	7.84E-02	6.06E-02

EGF/EGFR signaling pathway	*AP2B1,ERBB2,GAB2,PTK2,RAF1*	WP	0.0021	130	51	9.80E-02	3.85E-02
ErbB signaling pathway	*NRG1,ERBB2,PTK2,RAF1*	WP	0.0023	71	51	7.84E-02	5.63E-02
GRB7 events in ERBB2 signaling	*NRG1,ERBB2*	REAC	0.0023	3	51	3.92E-02	6.67E-01
EGFR tyrosine kinase inhibitor resistance	*NRG1,KDR,ERBB2,RAF1*	KEGG	0.0023	67	51	7.84E-02	5.97E-02
ErbB signaling pathway	*NRG1,ERBB2,PTK2,RAF1*	KEGG	0.0023	68	51	7.84E-02	5.88E-02
MicroRNAs in cancer	*ATM,WNT3,ERBB2,CDC25A,RAF1*	KEGG	0.0023	121	51	9.80E-02	4.13E-02
Infectious disease	*ADORA2B,AAAS,CHMP4B,NUP88,SFTPD, AP2B1,GTF2H1,PTK2,NUP54,XRCC4*	REAC	0.0024	617	51	1.96E-01	1.62E-02
Interactions of Vpr with host cellular proteins	*AAAS,NUP88,NUP54*	REAC	0.0024	26	51	5.88E-02	1.15E-01
Diseases of signal transduction by growth factor receptors and second messengers	*NRG1,KDR,ERBB2,GAB2,TNKS2,FGFR1,RAF1*	REAC	0.0024	306	51	1.37E-01	2.29E-02
Export of Viral Ribonucleoproteins from Nucleus	*AAAS,NUP88,NUP54*	REAC	0.0024	25	51	5.88E-02	1.20E-01
SUMOylation of DNA damage response and repair proteins	*AAAS,NUP88,NUP54,XRCC4*	REAC	0.0024	60	51	7.84E-02	6.67E-02
Nuclear import of Rev protein	*AAAS,NUP88,NUP54*	REAC	0.0024	25	51	5.88E-02	1.20E-01
Rev-mediated nuclear export of HIV RNA	*AAAS,NUP88,NUP54*	REAC	0.0024	27	51	5.88E-02	1.11E-01
SUMO E3 ligases SUMOylate target proteins	*AAAS,NUP88,PPARGC1A,NUP54,XRCC4*	REAC	0.0024	123	51	9.80E-02	4.07E-02
SUMOylation	*AAAS,NUP88,PPARGC1A,NUP54,XRCC4*	REAC	0.0024	129	51	9.80E-02	3.88E-02
SUMOylation of SUMOylation proteins	*AAAS,NUP88,NUP54*	REAC	0.0024	27	51	5.88E-02	1.11E-01
Transport of Ribonucleoproteins into the Host Nucleus	*AAAS,NUP88,NUP54*	REAC	0.0024	26	51	5.88E-02	1.15E-01

Transport of the SLBP independent Mature mRNA	*AAAS,NUP88,NUP54*	REAC	0.0024	27	51	5.88E-02	1.11E-01
Vpr-mediated nuclear import of PICs	*AAAS,NUP88,NUP54*	REAC	0.0024	25	51	5.88E-02	1.20E-01
NEP/NS2 Interacts with the Cellular Export Machinery	*AAAS,NUP88,NUP54*	REAC	0.0024	25	51	5.88E-02	1.20E-01
Defective TPR may confer susceptibility towards thyroid papillary carcinoma (TPC)	*AAAS,NUP88,NUP54*	REAC	0.0024	25	51	5.88E-02	1.20E-01
Regulation of Glucokinase by Glucokinase Regulatory Protein	*AAAS,NUP88,NUP54*	REAC	0.0024	25	51	5.88E-02	1.20E-01
Cellular response to heat stress	*AAAS,ATM,NUP88,NUP54*	REAC	0.0024	63	51	7.84E-02	6.35E-02
DNA Repair	*ATM,INO80E,POLE,GTF2H1,REV3L,XRCC4*	REAC	0.0024	225	51	1.18E-01	2.67E-02
Transport of the SLBP Dependant Mature mRNA	*AAAS,NUP88,NUP54*	REAC	0.0024	28	51	5.88E-02	1.07E-01
Interactions of Rev with host cellular proteins	*AAAS,NUP88,NUP54*	REAC	0.0024	28	51	5.88E-02	1.07E-01
HCMV Infection	*AAAS,CHMP4B,NUP88,NUP54*	REAC	0.0024	74	51	7.84E-02	5.41E-02
Nuclear Pore Complex (NPC) Disassembly	*AAAS,NUP88,NUP54*	REAC	0.0024	29	51	5.88E-02	1.03E-01
Nicotinate and nicotinamide metabolism	*CD38,NADSYN1,SIRT3*	KEGG	0.0027	28	51	5.88E-02	1.07E-01
SUMOylation of ubiquitinylation proteins	*AAAS,NUP88,NUP54*	REAC	0.0028	31	51	5.88E-02	9.68E-02
Viral Messenger RNA Synthesis	*AAAS,NUP88,NUP54*	REAC	0.0028	31	51	5.88E-02	9.68E-02
Hepatocyte growth factor receptor signaling	*PTK2,RAPGEF1,RAF1*	WP	0.0029	30	51	5.88E-02	1.00E-01
SARS-CoV-2 activates/modulates innate and adaptive immune responses	*AAAS,NUP88,SFTPD,NUP54*	REAC	0.0031	82	51	7.84E-02	4.88E-02

NS1 Mediated Effects on Host Pathways	*AAAS,NUP88,NUP54*	REAC	0.0032	33	51	5.88E-02	9.09E-02
Transport of Mature mRNA Derived from an Intronless Transcript	*AAAS,NUP88,NUP54*	REAC	0.0034	34	51	5.88E-02	8.82E-02
Androgen receptor network in prostate cancer	*ATM,CDC25A,RAPGEF1,RAF1*	WP	0.0034	82	51	7.84E-02	4.88E-02
SUMOylation of RNA binding proteins	*AAAS,NUP88,NUP54*	REAC	0.0034	35	51	5.88E-02	8.57E-02
Transport of Mature mRNAs Derived from Intronless Transcripts	*AAAS,NUP88,NUP54*	REAC	0.0034	35	51	5.88E-02	8.57E-02
SUMOylation of DNA replication proteins	*AAAS,NUP88,NUP54*	REAC	0.0034	35	51	5.88E-02	8.57E-02
Signaling by Non-Receptor Tyrosine Kinases	*GPNMB,NRG1,ERBB2*	REAC	0.0034	36	51	5.88E-02	8.33E-02
Signaling by PTK6	*GPNMB,NRG1,ERBB2*	REAC	0.0034	36	51	5.88E-02	8.33E-02
Host Interactions of HIV factors	*AAAS,NUP88,AP2B1,NUP54*	REAC	0.0034	88	51	7.84E-02	4.55E-02
Signaling by Receptor Tyrosine Kinases	*NRG1,KDR,AP2B1,ERBB2,GAB2,PTK2, RAPGEF1*	REAC	0.0036	367	51	1.37E-01	1.91E-02
Transcriptional regulation by small RNAs	*AAAS,NUP88,NUP54*	REAC	0.0036	38	51	5.88E-02	7.89E-02
Downregulation of ERBB2:ERBB3 signaling	*NRG1,ERBB2*	REAC	0.0036	8	51	3.92E-02	2.50E-01
Metabolism of non-coding RNA	*AAAS,NUP88,NUP54*	REAC	0.0036	39	51	5.88E-02	7.69E-02
snRNP Assembly	*AAAS,NUP88,NUP54*	REAC	0.0036	39	51	5.88E-02	7.69E-02
Cell-Cell communication	*SFTPD,PTK2,CDH3,NPHS1*	REAC	0.0036	93	51	7.84E-02	4.30E-02
SARS-CoV Infections	*AAAS,CHMP4B,NUP88,SFTPD,AP2B1,NUP54*	REAC	0.0036	261	51	1.18E-01	2.30E-02
Processing of Capped Intron-Containing Pre-mRNA	*AAAS,NUP88,SNRPC,TXNL4A,NUP54*	REAC	0.0036	171	51	9.80E-02	2.92E-02
Focal adhesion	*KDR,ERBB2,PTK2,RAPGEF1,RAF1*	WP	0.0038	159	51	9.80E-02	3.14E-02

Nuclear Envelope Breakdown	*AAAS,NUP88,NUP54*	REAC	0.0038	40	51	5.88E-02	7.50E-02
Focal adhesion	*KDR,ERBB2,PTK2,RAPGEF1,RAF1*	KEGG	0.0040	163	51	9.80E-02	3.07E-02
Breast cancer	*WNT3,ERBB2,FGFR1,RAF1*	KEGG	0.0040	89	51	7.84E-02	4.49E-02
Rap1 signaling pathway	*ADORA2B,KDR,FGFR1,RAPGEF1,RAF1*	KEGG	0.0040	163	51	9.80E-02	3.07E-02
SUMOylation of chromatin organization proteins	*AAAS,NUP88,NUP54*	REAC	0.0043	42	51	5.88E-02	7.14E-02
ERBB2 Activates PTK6 Signaling	*NRG1,ERBB2*	REAC	0.0043	9	51	3.92E-02	2.22E-01
IL-3 signaling pathway	*GAB2,RAPGEF1,RAF1*	WP	0.0044	37	51	5.88E-02	8.11E-02
Constitutive Signaling by Aberrant PI3K in Cancer	*NRG1,ERBB2,GAB2*	REAC	0.0048	44	51	5.88E-02	6.82E-02
ERBB2 Regulates Cell Motility	*NRG1,ERBB2*	REAC	0.0049	10	51	3.92E-02	2.00E-01
GRB2 events in ERBB2 signaling	*NRG1,ERBB2*	REAC	0.0049	10	51	3.92E-02	2.00E-01
tRNA processing in the nucleus	*AAAS,NUP88,NUP54*	REAC	0.0049	45	51	5.88E-02	6.67E-02
Hippo-Merlin signaling dysregulation	*KDR,PTK2,CDH3,FGFR1*	WP	0.0049	97	51	7.84E-02	4.12E-02
Netrin-UNC5B signaling pathway	*KDR,PTK2,RAF1*	WP	0.0049	40	51	5.88E-02	7.50E-02
Markers of kidney cell lineage	*KDR,AP2B1,NPHS1*	WP	0.0050	41	51	5.88E-02	7.32E-02
Physico-chemical features and toxicity-associated pathways	*ERBB2,PTK2,RAF1*	WP	0.0051	42	51	5.88E-02	7.14E-02
G1 to S cell cycle control	*ATM,POLE,CDC25A*	WP	0.0052	43	51	5.88E-02	6.98E-02
Calcium signaling pathway	*CD38,ADORA2B,KDR,ERBB2,FGFR1*	KEGG	0.0052	180	51	9.80E-02	2.78E-02
VEGF signaling pathway	*KDR,PTK2,RAF1*	KEGG	0.0052	41	51	5.88E-02	7.32E-02
Signal Transduction	*GPNMB,ARHGAP27,ADORA2B,AAAS,RBBP5, NRG1,KDR,WNT3,AP2B1,ERBB2,GAB2,TNKS2 ,PTK2,RAPGEF1,RAF1*	REAC	0.0054	1701	51	2.94E-01	8.82E-03
miRNAs involved in DNA damage response	*ATM,CDC25A*	WP	0.0057	10	51	3.92E-02	2.00E-01
PI3K events in ERBB2 signaling	*NRG1,ERBB2*	REAC	0.0057	11	51	3.92E-02	1.82E-01
SARS-CoV-2 Infection	*AAAS,CHMP4B,NUP88,SFTPD,NUP54*	REAC	0.0057	198	51	9.80E-02	2.53E-02

Transport of Mature mRNA derived from an Intron-Containing Transcript	*AAAS,NUP88,NUP54*	REAC	0.0058	49	51	5.88E-02	6.12E-02
Glycolysis	*AAAS,NUP88,NUP54*	REAC	0.0061	50	51	5.88E-02	6.00E-02
Endometrial cancer	*ERBB2,FGFR1,RAF1*	WP	0.0065	48	51	5.88E-02	6.25E-02
Signal regulatory protein family interactions	*SFTPD,PTK2*	REAC	0.0065	12	51	3.92E-02	1.67E-01
MET in type 1 papillary renal cell carcinoma	*PTK2,RAPGEF1,RAF1*	WP	0.0070	50	51	5.88E-02	6.00E-02
NAD+ metabolism	*CD38,SIRT3*	WP	0.0073	12	51	3.92E-02	1.67E-01
DCC mediated attractive signaling	*PTK2,DCC*	REAC	0.0075	13	51	3.92E-02	1.54E-01
ISG15 antiviral mechanism	*AAAS,NUP88,NUP54*	REAC	0.0077	55	51	5.88E-02	5.45E-02
Cell Cycle	*AAAS,CHMP4B,ATM,NUP88,POLE,CDC25A, NUP54*	REAC	0.0081	453	51	1.37E-01	1.55E-02
Signaling by ERBB2 TMD/JMD mutants	*NRG1,ERBB2*	REAC	0.0081	14	51	3.92E-02	1.43E-01
SHC1 events in ERBB2 signaling	*NRG1,ERBB2*	REAC	0.0081	14	51	3.92E-02	1.43E-01
SARS-CoV-2-host interactions	*AAAS,NUP88,SFTPD,NUP54*	REAC	0.0081	128	51	7.84E-02	3.13E-02
Platinum drug resistance	*ATM,ERBB2,REV3L*	KEGG	0.0082	50	51	5.88E-02	6.00E-02
HCMV Early Events	*AAAS,NUP88,NUP54*	REAC	0.0082	58	51	5.88E-02	5.17E-02
Transport of Mature Transcript to Cytoplasm	*AAAS,NUP88,NUP54*	REAC	0.0082	58	51	5.88E-02	5.17E-02
Overview of nanoparticle effects	*PTK2,CDH3*	WP	0.0082	13	51	3.92E-02	1.54E-01
Metabolism of proteins	*RAB29,ADORA2B,AAAS,RBBP5,NUP88,INO80E,SFTPD,CDC25A,TNKS2,PPARGC1A,NUP54, XRCC4*	REAC	0.0084	1251	51	2.35E-01	9.59E-03
Antiviral mechanism by IFN-stimulated genes	*AAAS,NUP88,NUP54*	REAC	0.0088	60	51	5.88E-02	5.00E-02
Gene Silencing by RNA	*AAAS,NUP88,NUP54*	REAC	0.0094	62	51	5.88E-02	4.84E-02

Glucose metabolism	*AAAS,NUP88,NUP54*	REAC	0.0094	62	51	5.88E-02	4.84E-02
Global Genome Nucleotide Excision Repair (GG-NER)	*INO80E,POLE,GTF2H1*	REAC	0.0101	64	51	5.88E-02	4.69E-02
PI3K/AKT Signaling in Cancer	*NRG1,ERBB2,GAB2*	REAC	0.0101	64	51	5.88E-02	4.69E-02
Focal adhesion: PI3K-Akt-mTOR-signaling pathway	*KDR,PTK2,FGFR1,PPARGC1A,RAF1*	WP	0.0102	228	51	9.80E-02	2.19E-02
Ras signaling	*KDR,GAB2,FGFR1,RAF1*	WP	0.0102	133	51	7.84E-02	3.01E-02
SLC transporter disorders	*AAAS,NUP88,NUP54*	REAC	0.0104	65	51	5.88E-02	4.62E-02
Signaling by ERBB2 KD Mutants	*NRG1,ERBB2*	REAC	0.0105	17	51	3.92E-02	1.18E-01
Thyroid hormones production and peripheral downstream signaling effects	*FGFR1,PPARGC1A,RAF1*	WP	0.0109	63	51	5.88E-02	4.76E-02
Leptin signaling pathway	*ERBB2,PTK2,RAF1*	WP	0.0109	63	51	5.88E-02	4.76E-02
Mitotic Prophase	*AAAS,NUP88,NUP54*	REAC	0.0110	67	51	5.88E-02	4.48E-02
Cell Cycle, Mitotic	*AAAS,CHMP4B,NUP88,POLE,CDC25A,NUP54*	REAC	0.0111	364	51	1.18E-01	1.65E-02
PI5P, PP2A and IER3 Regulate PI3K/AKT Signaling	*NRG1,ERBB2,GAB2*	REAC	0.0111	68	51	5.88E-02	4.41E-02
Post-translational protein modification	*RAB29,AAAS,RBBP5,NUP88,INO80E,CDC25A,TNKS2,PPARGC1A,NUP54,XRCC4*	REAC	0.0111	961	51	1.96E-01	1.04E-02
Signaling by ERBB2 in Cancer	*NRG1,ERBB2*	REAC	0.0111	18	51	3.92E-02	1.11E-01
Downregulation of ERBB2 signaling	*NRG1,ERBB2*	REAC	0.0121	19	51	3.92E-02	1.05E-01
Surfactant metabolism	*ADORA2B,SFTPD*	REAC	0.0121	19	51	3.92E-02	1.05E-01
Retinoblastoma gene in cancer	*POLE,CDC25A,RAF1*	WP	0.0126	67	51	5.88E-02	4.48E-02
Negative regulation of the PI3K/AKT network	*NRG1,ERBB2,GAB2*	REAC	0.0132	74	51	5.88E-02	4.05E-02
Influenza Viral RNA Transcription and Replication	*AAAS,NUP88,NUP54*	REAC	0.0132	74	51	0.0588	0.0405
FGFR1 mutant receptor activation	*GAB2,FGFR1*	REAC	0.0132	20	51	0.0392	0.1000

tRNA processing	*AAAS,NUP88,NUP54*	REAC	0.0135	75	51	0.0588	0.0400
Chromatin organization	*KANSL1,KAT8,RBBP5,SETD1A*	REAC	0.0144	162	51	0.0784	0.0247
Chromatin modifying enzymes	*KANSL1,KAT8,RBBP5,SETD1A*	REAC	0.0144	162	51	0.0784	0.0247
FOXO-mediated transcription of oxidative stress, metabolic and neuronal genes	*SIRT3,PPARGC1A*	REAC	0.0150	22	51	0.0392	0.0909
PI3K-Akt signaling pathway	*KDR,ERBB2,PTK2,FGFR1,RAF1*	KEGG	0.0152	243	51	0.0980	0.0206
Pathways in cancer	*WNT3,ERBB2,PTK2,FGFR1,DCC,RAF1*	KEGG	0.0152	361	51	0.1176	0.0166
Endocrine resistance	*ERBB2,PTK2,RAF1*	KEGG	0.0152	64	51	0.0588	0.0469
Nucleotide Excision Repair	*INO80E,POLE,GTF2H1*	REAC	0.0160	81	51	0.0588	0.0370
Nicotinate metabolism	*CD38,NADSYN1*	REAC	0.0160	23	51	0.0392	0.0870
B cell receptor signaling pathway	*GAB2,RAPGEF1,RAF1*	WP	0.0162	74	51	0.0588	0.0405
Embryonic stem cell pluripotency pathways	*WNT3,FGFR1,RAF1*	WP	0.0163	75	51	0.0588	0.0400
Prostate cancer	*ERBB2,FGFR1,RAF1*	KEGG	0.0166	70	51	0.0588	0.0429
Gene expression (Transcription)	*AAAS,ATM,SIRT3,RBBP5,NUP88,SETD1A, ERBB2,GTF2H1,PPARGC1A,NUP54*	REAC	0.0174	1043	51	0.1961	0.0096
DNA damage response (only ATM dependent)	*ATM,WNT3,ERBB2*	WP	0.0174	80	51	0.0588	0.0375
Bladder cancer	*ERBB2,RAF1*	WP	0.0174	22	51	0.0392	0.0909
Integrin-mediated cell adhesion	*PTK2,RAPGEF1,RAF1*	WP	0.0174	80	51	0.0588	0.0375
Angiogenesis	*KDR,PTK2*	WP	0.0174	22	51	0.0392	0.0909
Nanoparticle-mediated activation of receptor signaling	*PTK2,RAF1*	WP	0.0176	23	51	0.0392	0.0870
Hippo signaling regulation pathways	*KDR,CDH3,FGFR1*	WP	0.0183	83	51	0.0588	0.0361
Signaling by FGFR1 in disease	*GAB2,FGFR1*	REAC	0.0185	25	51	0.0392	0.0800
Influenza Infection	*AAAS,NUP88,NUP54*	REAC	0.0189	87	51	0.0588	0.0345
Genes controlling nephrogenesis	*KDR,NPHS1*	WP	0.0192	25	51	0.0392	0.0800

Extracellular vesicle-mediated signaling in recipient cells	*ERBB2,RAF1*	WP	0.0192	25	51	0.0392	0.0800
Platelet activation, signaling and aggregation	*MMRN1,STX4,PTK2,RAF1*	REAC	0.0195	180	51	0.0784	0.0222
RAF/MAP kinase cascade	*NRG1,ERBB2,PTK2,RAF1*	REAC	0.0201	182	51	0.0784	0.0220
PIP3 activates AKT signaling	*NRG1,ERBB2,GAB2,TNKS2*	REAC	0.0202	183	51	0.0784	0.0219
Cytokine Signaling in Immune system	*STX4,AAAS,NUP88,GAB2,RAPGEF1,NUP54*	REAC	0.0202	430	51	0.1176	0.0140
Metabolism of RNA	*AAAS,NUP88,GTF2H1,SNRPC,TXNL4A,NUP54*	REAC	0.0202	431	51	0.1176	0.0139
MAPK1/MAPK3 signaling	*NRG1,ERBB2,PTK2,RAF1*	REAC	0.0208	186	51	0.0784	0.0215
Prion disease pathway	*PTK2,FGFR1*	WP	0.0214	27	51	0.0392	0.0741
Cardiac progenitor differentiation	*NRG1,KDR*	WP	0.0214	27	51	0.0392	0.0741
Nucleocytoplasmic transport	*AAAS,NUP88,NUP54*	KEGG	0.0215	80	51	0.0588	0.0375
Bladder cancer	*ERBB2,RAF1*	KEGG	0.0215	23	51	0.0392	0.0870
Ras signaling pathway	*KDR,GAB2,FGFR1,RAF1*	KEGG	0.0215	167	51	0.0784	0.0240
Signaling pathways regulating pluripotency of stem cells	*WNT3,FGFR1,RAF1*	KEGG	0.0234	85	51	0.0588	0.0353
IL-2 signaling pathway	*GAB2,RAF1*	WP	0.0240	29	51	0.0392	0.0690
Translesion synthesis by Y family DNA polymerases bypasses lesions on DNA template	*POLE,REV3L*	REAC	0.0245	30	51	0.0392	0.0667
ATM signaling pathway	*ATM,CDC25A*	WP	0.0251	30	51	0.0392	0.0667
Signaling by WNT	*RBBP5,WNT3,AP2B1,TNKS2*	REAC	0.0255	198	51	0.0784	0.0202
Dual Incision in GG-NER	*POLE,GTF2H1*	REAC	0.0257	31	51	0.0392	0.0645
Interleukin-3, Interleukin-5 and GM-CSF signaling	*GAB2,RAPGEF1*	REAC	0.0267	32	51	0.0392	0.0625
Cellular responses to stress	*AAAS,ATM,NUP88,LIMD1,PPARGC1A,NUP54*	REAC	0.0267	463	51	0.1176	0.0130
Nonhomologous End-Joining (NHEJ)	*ATM,XRCC4*	REAC	0.0267	32	51	0.0392	0.0625

Developmental Biology	*RBBP5,AP2B1,ERBB2,GAB2,PTK2,PPARGC1A, DCC*	REAC	0.0268	616	51	0.1373	0.0114
Cellular responses to stimuli	*AAAS,ATM,NUP88,LIMD1,PPARGC1A,NUP54*	REAC	0.0268	465	51	0.1176	0.0129
SNARE interactions in vesicular transport	*STX4,VTI1A*	KEGG	0.0269	27	51	0.0392	0.0741
RUNX1 regulates genes involved in megakaryocyte differentiation and platelet function	*RBBP5,SETD1A*	REAC	0.0275	33	51	0.0392	0.0606
Gastric cancer	*WNT3,ERBB2,RAF1*	KEGG	0.0283	94	51	0.0588	0.0319
Signaling by ERBB2	*NRG1,ERBB2*	REAC	0.0289	34	51	0.0392	0.0588
Signaling by plasma membrane FGFR1 fusions	*FGFR1*	REAC	0.0311	2	51	0.0196	0.5000
XAV939 stabilizes AXIN	*TNKS2*	REAC	0.0311	2	51	0.0196	0.5000
PKMTs methylate histone lysines	*RBBP5,SETD1A*	REAC	0.0312	36	51	0.0392	0.0556
MAPK family signaling cascades	*NRG1,ERBB2,PTK2,RAF1*	REAC	0.0312	216	51	0.0784	0.0185
Nucleotide excision repair	*POLE,GTF2H1*	WP	0.0313	34	51	0.0392	0.0588
DNA Double-Strand Break Repair	*ATM,POLE,XRCC4*	REAC	0.0314	112	51	0.0588	0.0268
Regulation of actin cytoskeleton	*PTK2,FGFR1,RAF1*	WP	0.0316	108	51	0.0588	0.0278
Integrated cancer pathway	*ATM,CDC25A*	WP	0.0318	35	51	0.0392	0.0571
Intracellular signaling by second messengers	*NRG1,ERBB2,GAB2,TNKS2*	REAC	0.0320	219	51	0.0784	0.0183
Signaling by FGFR in disease	*GAB2,FGFR1*	REAC	0.0320	37	51	0.0392	0.0541
Disorders of transmembrane transporters	*AAAS,NUP88,NUP54*	REAC	0.0322	114	51	0.0588	0.0263
Insulin signaling pathway	*PPARGC1A,RAPGEF1,RAF1*	KEGG	0.0328	101	51	0.0588	0.0297
MAPK signaling pathway	*KDR,ERBB2,FGFR1,RAF1*	KEGG	0.0328	202	51	0.0784	0.0198
DNA Damage Bypass	*POLE,REV3L*	REAC	0.0329	38	51	0.0392	0.0526

Transcriptional activation of mitochondrial biogenesis	*SIRT3,PPARGC1A*	REAC	0.0329	38	51	0.0392	0.0526
MET promotes cell motility	*PTK2,RAPGEF1*	REAC	0.0341	39	51	0.0392	0.0513
FOXO-mediated transcription	*SIRT3,PPARGC1A*	REAC	0.0341	39	51	0.0392	0.0513
Energy metabolism	*SIRT3,PPARGC1A*	WP	0.0347	37	51	0.0392	0.0541
Nucleotide excision repair	*POLE,GTF2H1*	KEGG	0.0358	34	51	0.0392	0.0588
Apoptosis-related network due to altered Notch3 in ovarian cancer	*NRG1,PTK2*	WP	0.0376	39	51	0.0392	0.0513
Primary ovarian insufficiency	*ATM,KDR,XRCC4*	WP	0.0390	121	51	0.0588	0.0248
PI3K-Akt signaling pathway	*KDR,PTK2,FGFR1,RAF1*	WP	0.0390	234	51	0.0784	0.0171
Insulin signaling	*STX4,RAPGEF1,RAF1*	WP	0.0390	122	51	0.0588	0.0246
NRP1-triggered signaling pathways in pancreatic cancer	*KDR,PTK2*	WP	0.0390	41	51	0.0392	0.0488
TCF dependent signaling in response to WNT	*RBBP5,WNT3,TNKS2*	REAC	0.0402	126	51	0.0588	0.0238
G1/S DNA Damage Checkpoints	*ATM,CDC25A*	REAC	0.0402	43	51	0.0392	0.0465
Interferon Signaling	*AAAS,NUP88,NUP54*	REAC	0.0402	126	51	0.0588	0.0238
RANKL/RANK signaling pathway	*GAB2,PTK2*	WP	0.0405	43	51	0.0392	0.0465
Interferon type I signaling pathways	*GAB2,RAPGEF1*	WP	0.0405	43	51	0.0392	0.0465
Cellular senescence	*ATM,CDC25A,RAF1*	KEGG	0.0412	114	51	0.0588	0.0263
Gap-filling DNA repair synthesis and ligation in TC-NER	*POLE,GTF2H1*	REAC	0.0414	44	51	0.0392	0.0455
Netrin-1 signaling	*PTK2,DCC*	REAC	0.0414	44	51	0.0392	0.0455
Dual incision in TC-NER	*POLE,GTF2H1*	REAC	0.0425	45	51	0.0392	0.0444
TP53 Regulates Transcription of DNA Repair Genes	*ATM,GTF2H1*	REAC	0.0425	45	51	0.0392	0.0444
miRNA regulation of DNA damage response	*ATM,CDC25A*	WP	0.0437	46	51	0.0392	0.0435

Cardiac hypertrophic response	*NRG1,RAF1*	WP	0.0437	46	51	0.0392	0.0435
DNA damage response	*ATM,CDC25A*	WP	0.0437	46	51	0.0392	0.0435
Immune System	*STX4,AAAS,NUP88,SFTPD,AP2B1,GAB2,PTK2, RAPGEF1,NUP54,RAF1*	REAC	0.0441	1252	51	0.1961	0.0080
17q12 copy number variation syndrome	*AP2B1,ERBB2*	WP	0.0447	47	51	0.0392	0.0426
Kit receptor signaling pathway	*GAB2,RAF1*	WP	0.0451	48	51	0.0392	0.0417
Nucleotide excision repair in xeroderma pigmentosum	*POLE,GTF2H1*	WP	0.0451	48	51	0.0392	0.0417
Human papillomavirus infection	*ATM,WNT3,PTK2,RAF1*	KEGG	0.0467	231	51	0.0784	0.0173
Oxytocin signaling	*CD38*	WP	0.0475	4	51	0.0196	0.2500
Melanoma	*KDR,RAF1*	WP	0.0475	51	51	0.0392	0.0392
SARS-CoV-2 altering angiogenesis via NRP1	*KDR*	WP	0.0475	4	51	0.0196	0.2500
Chromosomal and microsatellite instability in colorectal cancer	*DCC,RAF1*	WP	0.0475	51	51	0.0392	0.0392
2-LTR circle formation	*XRCC4*	REAC	0.0476	4	51	0.0196	0.2500
Resistance of ERBB2 KD mutants to lapatinib	*ERBB2*	REAC	0.0476	4	51	0.0196	0.2500
Resistance of ERBB2 KD mutants to osimertinib	*ERBB2*	REAC	0.0476	4	51	0.0196	0.2500
Resistance of ERBB2 KD mutants to AEE788	*ERBB2*	REAC	0.0476	4	51	0.0196	0.2500
Drug resistance in ERBB2 TMD/JMD mutants	*ERBB2*	REAC	0.0476	4	51	0.0196	0.2500
Adenosine P1 receptors	*ADORA2B*	REAC	0.0476	4	51	0.0196	0.2500
Drug-mediated inhibition of ERBB2 signaling	*ERBB2*	REAC	0.0476	4	51	0.0196	0.2500
PLCG1 events in ERBB2 signaling	*ERBB2*	REAC	0.0476	4	51	0.0196	0.2500

Resistance of ERBB2 KD mutants to neratinib	*ERBB2*	REAC	0.0476	4	51	0.0196	0.2500
Resistance of ERBB2 KD mutants to trastuzumab	*ERBB2*	REAC	0.0476	4	51	0.0196	0.2500
Nuclear Envelope (NE) Reassembly	*CHMP4B,NUP54*	REAC	0.0476	50	51	0.0392	0.0400
Resistance of ERBB2 KD mutants to tesevatinib	*ERBB2*	REAC	0.0476	4	51	0.0196	0.2500
Axon guidance	*AP2B1,ERBB2,GAB2,PTK2,DCC*	REAC	0.0476	394	51	0.0980	0.0127
Resistance of ERBB2 KD mutants to sapitinib	*ERBB2*	REAC	0.0476	4	51	0.0196	0.2500
Neurophilin interactions with VEGF and VEGFR	*KDR*	REAC	0.0476	4	51	0.0196	0.2500
Drug resistance in ERBB2 KD mutants	*ERBB2*	REAC	0.0476	4	51	0.0196	0.2500
Resistance of ERBB2 KD mutants to afatinib	*ERBB2*	REAC	0.0476	4	51	0.0196	0.2500
M Phase	*AAAS,CHMP4B,NUP88,NUP54*	REAC	0.0476	264	51	0.0784	0.0152

Supplementary Table 7. Biological pathways enriched for PD-associated network genes.

Pathway description	Genes	source	p_value	term_size	query_size	precision	recall
Immune System	CTSB,SH3GL2,HLA-DRB1,STX4,MEX3C,IP6K2,ARIH2,AREL1,ALPK1,PRSS3,AMPD3,NOD2,AAAS,HLA-DMA,YWHAB,CTSH,NUP88,PNP,TICAM1,EP300,PSMB7,RAB5C,GAB2,USP14	REAC	6.15E-05	1252	86	2.79E-01	1.92E-02
SNARE interactions in vesicular transport	STX4,VTI1A,STX6,YKT6	KEGG	0.0016	27	86	4.65E-02	1.48E-01
Tuberculosis	HLA-DRB1,ARHGEF12,NOD2,HLA-DMA,EP300,RAB5C	KEGG	0.0021	105	86	6.98E-02	5.71E-02
Antigen processing and presentation	CTSB,HLA-DRB1,HLA-DMA,HSPA4	KEGG	0.0046	42	86	4.65E-02	9.52E-02
Mitotic G1 phase and G1/S transition	TK1,DYRK1A,POLE,PRIM1,PSMB7,E2F3	REAC	0.0059	99	86	6.98E-02	6.06E-02
Cell Cycle, Mitotic	TK1,DYRK1A,POLE,AAAS,NUP88,PRIM1,EP300,PSMB7,FEN1,E2F3	REAC	0.0100	364	86	1.16E-01	2.75E-02
Cell Cycle	TK1,DYRK1A,POLE,AAAS,YWHAB,NUP88,PRIM1,EP300,PSMB7,FEN1,E2F3	REAC	0.0102	453	86	1.28E-01	2.43E-02
Influenza A	HLA-DRB1,PRSS3,HLA-DMA,TICAM1,EP300	KEGG	0.0108	100	86	5.81E-02	5.00E-02
Innate Immune System	CTSB,ALPK1,PRSS3,AMPD3,NOD2,CTSH,PNP,TICAM1,EP300,PSMB7,RAB5C,GAB2,USP14	REAC	0.0112	639	86	1.51E-01	2.03E-02
Nucleotide salvage	TK1,AMPD3,PNP	REAC	0.0121	17	86	3.49E-02	1.76E-01
Nicotinate and nicotinamide metabolism	CD38,NADSYN1,PNP	KEGG	0.0143	28	86	3.49E-02	1.07E-01
DNA replication	POLE,PRIM1,FEN1	KEGG	0.0143	28	86	3.49E-02	1.07E-01
Pathways affected in adenoid cystic carcinoma	KANSL1,ERBB2,EP300,MGA	WP	0.0162	51	86	4.65E-02	7.84E-02
Allograft rejection	LRRK2,HLA-DRB1,HLA-DMA,CASP7	WP	0.0162	47	86	4.65E-02	8.51E-02

Acute viral myocarditis	NOD2,HLA-DMA,TICAM1,CASP7	WP	0.0175	56	86	4.65E-02	7.14E-02
Adaptive Immune System	CTSB,SH3GL2,HLA-DRB1,STX4, MEX3C,ARIH2,AREL1,HLA-DMA, YWHAB,CTSH,PSMB7	REAC	0.0184	541	86	1.28E-01	2.03E-02
MHC class II antigen presentation	CTSB,SH3GL2,HLA-DRB1,HLA-DMA,CTSH	REAC	0.0184	97	86	5.81E-02	5.15E-02
trans-Golgi Network Vesicle Budding	SH3GL2,STX4,GAK,RAB5C	REAC	0.0184	54	86	4.65E-02	7.41E-02
Cytokine Signaling in Immune system	HLA-DRB1,STX4,IP6K2,ALPK1,NOD2,AAAS, NUP88,PSMB7,GAB2,USP14	REAC	0.0184	430	86	1.16E-01	2.33E-02
PTK6 promotes HIF1A stabilization	LRRK2,GPNMB	REAC	0.0184	5	86	2.33E-02	4.00E-01
Phase 1- inactivation of fast Na+ channels	KCNIP3,KCNIP4	REAC	0.0184	5	86	2.33E-02	4.00E-01
Cellular response to heat stress	AAAS,NUP88,EP300,HSPA4	REAC	0.0233	63	86	4.65E-02	6.35E-02
DNA replication initiation	POLE,PRIM1	REAC	0.0233	6	86	2.33E-02	3.33E-01
Toll-like Receptor Cascades	CTSB,ALPK1,NOD2,TICAM1,USP14	REAC	0.0275	118	86	5.81E-02	4.24E-02
Toll Like Receptor 7/8 (TLR7/8) Cascade	ALPK1,NOD2,TICAM1,USP14	REAC	0.0275	71	86	4.65E-02	5.63E-02
TRAF6 mediated induction of NFkB and MAP kinases upon TLR7/8 or 9 activation	ALPK1,NOD2,TICAM1,USP14	REAC	0.0275	71	86	4.65E-02	5.63E-02
MyD88 dependent cascade initiated on endosome	ALPK1,NOD2,TICAM1,USP14	REAC	0.0275	71	86	4.65E-02	5.63E-02
TAK1-dependent IKK and NF-kappa-B activation	ALPK1,NOD2,USP14	REAC	0.0275	30	86	3.49E-02	1.00E-01
Inflammatory bowel disease	HLA-DRB1,NOD2,HLA-DMA	KEGG	0.0285	37	86	0.0349	0.0811
Purine salvage	AMPD3,PNP	REAC	0.0295	8	86	0.0233	0.2500
Signaling by Non-Receptor Tyrosine Kinases	LRRK2,GPNMB,ERBB2	REAC	0.0304	36	86	0.0349	0.0833
Signaling by PTK6	LRRK2,GPNMB,ERBB2	REAC	0.0304	36	86	0.0349	0.0833
MyD88-independent TLR4 cascade	ALPK1,NOD2,TICAM1,USP14	REAC	0.0304	79	86	0.0465	0.0506

TRIF(TICAM1)-mediated TLR4 signaling	ALPK1,NOD2,TICAM1,USP14	REAC	0.0304	79	86	0.0465	0.0506
Toll Like Receptor 3 (TLR3) Cascade	ALPK1,NOD2,TICAM1,USP14	REAC	0.0304	76	86	0.0465	0.0526
Toll Like Receptor 9 (TLR9) Cascade	ALPK1,NOD2,TICAM1,USP14	REAC	0.0304	75	86	0.0465	0.0533
Interleukin-1 signaling	ALPK1,NOD2,PSMB7,USP14	REAC	0.0307	80	86	0.0465	0.0500
Synthesis of DNA	POLE,PRIM1,PSMB7,FEN1	REAC	0.0325	82	86	0.0465	0.0488
Membrane Trafficking	SH3GL2,STX4,GAK,YWHAB,VTI1A,STX6, AMPH,RAB5C,YKT6	REAC	0.0368	465	86	0.1047	0.0194
Intra-Golgi traffic	VTI1A,STX6,YKT6	REAC	0.0368	40	86	0.0349	0.0750
G1/S Transition	TK1,POLE,PRIM1,PSMB7	REAC	0.0368	87	86	0.0465	0.0460
Ulcerative colitis signaling	NOD2,HLA-DMA	WP	0.0389	8	86	0.0233	0.2500
Golgi Associated Vesicle Biogenesis	SH3GL2,GAK,RAB5C	REAC	0.0390	42	86	0.0349	0.0714
DNA Replication	POLE,PRIM1,PSMB7,FEN1	REAC	0.0406	91	86	0.0465	0.0440
Sema4D induced cell migration and growth-cone collapse	ARHGEF12,ERBB2	REAC	0.0411	12	86	0.0233	0.1667
Signaling by NTRKs	SH3GL2,YWHAB,EP300,BDNF	REAC	0.0432	94	86	0.0465	0.0426
Removal of the Flap Intermediate	PRIM1,FEN1	REAC	0.0445	13	86	0.0233	0.1538
Vesicle-mediated transport	SH3GL2,STX4,GAK,YWHAB,VTI1A,STX6, AMPH,RAB5C,YKT6	REAC	0.0445	490	86	0.1047	0.0184
Neutrophil degranulation	CTSB,PRSS3,AMPD3,CTSH,PNP,PSMB7, RAB5C	REAC	0.0460	315	86	0.0814	0.0222
Processive synthesis on the lagging strand	PRIM1,FEN1	REAC	0.0492	14	86	0.0233	0.1429

Supplementary Table 8. Genes that are present in both PD-causal and PD-associated networks.

LINC02210	SETD1A	RAB29	GPNMB	GAK	VTI1A	CD38	GAB2	PRSS36
KANSL1	ERBB2	STX4	NUPL2	NADSYN1	POLE	AAAS	NUP88	WNT3

Supplementary Table 9. Biological pathways enriched for both PD-causal and associated network genes.

ID	Pathway description	source	PD-associated network genes	PD-causal network genes
REAC:R-HSA-168256	Immune System	REAC	CTSB,SH3GL2,HLA-DRB1,STX4,MEX3C, IP6K2,ARIH2,AREL1,ALPK1,PRSS3,AMPD3, NOD2,AAAS,HLA-DMA,YWHAB,CTSH, NUP88,PNP,TICAM1,EP300,PSMB7,RAB5C, GAB2,USP14	STX4,AAAS,NUP88,SFTPD,AP2B1,GAB2, PTK2,RAPGEF1,NUP54,RAF1
KEGG:04130	SNARE interactions in vesicular transport	KEGG	STX4,VTI1A,STX6,YKT6	STX4,VTI1A
REAC:R-HSA-69278	Cell Cycle, Mitotic	REAC	TK1,DYRK1A,POLE,AAAS,NUP88,PRIM1, EP300,PSMB7,FEN1,E2F3	AAAS,CHMP4B,NUP88,POLE,CDC25A, NUP54
REAC:R-HSA-1640170	Cell Cycle	REAC	TK1,DYRK1A,POLE,AAAS,YWHAB,NUP88, PRIM1,EP300,PSMB7,FEN1,E2F3	AAAS,CHMP4B,ATM,NUP88,POLE,CDC25A, NUP54
KEGG:00760	Nicotinate and nicotinamide metabolism	KEGG	CD38,NADSYN1,PNP	CD38,NADSYN1,SIRT3
WP:WP3651	Pathways affected in adenoid cystic carcinoma	WP	KANSL1,ERBB2,EP300,MGA	KANSL1,ATM,ERBB2,RAF1
REAC:R-HSA-1280215	Cytokine Signaling in Immune system	REAC	HLA-DRB1,STX4,IP6K2,ALPK1,NOD2,AAAS, NUP88,PSMB7,GAB2,USP14	STX4,AAAS,NUP88,GAB2,RAPGEF1,NUP54
REAC:R-HSA-3371556	Cellular response to heat stress	REAC	AAAS,NUP88,EP300,HSPA4	AAAS,ATM,NUP88,NUP54
REAC:R-HSA-9006927	Signaling by Non-Receptor Tyrosine Kinases	REAC	LRRK2,GPNMB,ERBB2	GPNMB,NRG1,ERBB2

REAC:R-HSA-8848021	Signaling by PTK6	REAC	LRRK2,GPNMB,ERBB2	GPNMB,NRG1,ERBB2

Supplementary Table 10. Diseases that comorbid with PD patients were identified by comorbidity analysis.

Disease code	Major class	descShort	log10_or_lower	log10_or_upper	log10_or
G218	Secondary parkinsonism	Other secondary parkinsonism	3.6997	7.3752	5.6220
T8384	Complications of genitourinary prosth dev/grft	Pain from genitourinary prosth dev/grft	2.6414	6.8235	4.9932
F028	Dementia in other diseases classified elsewhere	Dementia in other diseases classified elsewhere	4.6006	4.9628	4.7824
L8940	Pressure ulcer	Pressr ulc of contig site of back, buttock and hip, unsp stg	2.3674	6.3282	4.6568
C9420	Other leukemias of specified cell type	Acute megakaryoblastic leukemia not achieve remission	0.4434	6.4722	4.2999
M4319	Other deforming dorsopathies	Spondylolisthesis, multiple sites in spine	0.4434	6.4722	4.2999
G219	Secondary parkinsonism	Secondary parkinsonism, unspecified	0.4434	6.4722	4.2999
S880	Traumatic amputation of lower leg	Traumatic amputation at knee level	0.4434	6.4722	4.2999
M4606	Other inflammatory spondylopathies	Spinal enthesopathy, lumbar region	0.4434	6.4722	4.2999
Y753	Neurological devices associated with adverse incidents	Surgical instrumnt, matrl and neuro devices assoc w incdt	0.4434	6.4722	4.2999
F818	Specific developmental disorders of scholastic skills	Other developmental disorders of scholastic skills	0.2889	6.2253	4.1176
M8727	Osteonecrosis	Osteonecrosis due to previous trauma, ankle, foot and toes	0.2889	6.2253	4.1176
T690	Other effects of reduced temperature	Immersion hand and foot	0.2889	6.2253	4.1176
R049	Hemorrhage from respiratory passages	Hemorrhage from respiratory passages, unspecified	0.2889	6.2253	4.1176

M4848	Other spondylopathies	Fatigue fracture of vertebra, sacr/sacrocygl region	2.3141	4.3818	3.4764
Z739	Problems related to life management difficulty	Problem related to life management difficulty, unspecified	2.3767	4.2374	3.4115
Z749	Problems related to care provider dependency	Problem related to care provider dependency, unspecified	2.1577	4.2118	3.3148
M889	Osteitis deformans [Paget's disease of bone]	Osteitis deformans of unspecified bone	2.0746	4.1221	3.2290
L8944	Pressure ulcer	Pressr ulcer of contig site of back, buttock and hip, stg 4	1.7058	4.0185	3.0335
M4326	Other deforming dorsopathies	Fusion of spine, lumbar region	1.6656	3.9747	2.9921
R498	Voice and resonance disorders	Other voice and resonance disorders	1.6525	3.9607	2.9787
Z043	Encounter for examination and observation for other reasons	Encounter for exam and observation following oth accident	2.8748	3.0778	2.9771
Z738	Problems related to life management difficulty	Other problems related to life management difficulty	2.6420	3.2599	2.9624
S7203	Fracture of femur	Midcervical fracture of femur	2.7968	3.0411	2.9209
M8437	Disorder of continuity of bone	Stress fracture, ankle, foot and toes	1.2909	4.0177	2.8974
V632	Occupant of hv veh injured pick-up truck, pk-up/van	Person outside hv veh inj pick-up truck, pk-up/van nontraf	2.7620	2.9974	2.8811
S7204	Fracture of femur	Fracture of base of neck of femur	2.4861	3.2071	2.8621
H505	Other strabismus	Heterophoria	1.2444	3.9672	2.8494
H105	Conjunctivitis	Blepharoconjunctivitis	1.2293	3.9503	2.8339
F600	Specific personality disorders	Paranoid personality disorder	1.1851	3.9047	2.7888
Y9219	Place of occurrence of the external cause	Oth residential institution as place	1.4528	3.7487	2.7743
N489	Other disorders of penis	Disorder of penis, unspecified	1.1295	3.8436	2.7316
S0133	Open wound of head	Puncture wound without foreign body of ear	1.1295	3.8436	2.7316

L891	Pressure ulcer	Pressure ulcer of back	2.5533	2.8150	2.6857
Y733	Gastroenterology and urology devices assoc w incdt	Surg instrumnt, matrl & gastroent and urol dev assoc w incdt	1.0764	3.7873	2.6775
S799	Other and unspecified injuries of hip and thigh	Unspecified injury of hip and thigh	2.5272	2.7980	2.6647
R15	Fecal incontinence	Fecal incontinence	2.5220	2.7879	2.6569
L890	Pressure ulcer	Pressure ulcer of elbow	2.5058	2.7825	2.6462
E1143	Type 2 diabetes mellitus	Type 2 diabetes w diabetic autonomic (poly)neuropathy	2.5037	2.7682	2.6378
S0638	Intracranial injury	Contusion, laceration, and hemorrhage of brainstem	1.0021	3.7097	2.6016
T835	Complications of genitourinary prosth dev/grft	Infect/inflm reaction due to prosth dev/grft in urinry sys	2.4221	2.7118	2.5689
C700	Malignant neoplasm of meninges	Malignant neoplasm of cerebral meninges	0.4075	3.8724	2.5422
M8586	Other disorders of bone density and structure	Oth disrd of bone density and structure, lower leg	0.4075	3.8724	2.5422
E778	Disorders of glycoprotein metabolism	Other disorders of glycoprotein metabolism	0.3907	3.8544	2.5251
X110	Contact with hot tap-water	Contact with hot water in bath or tub	0.3743	3.8367	2.5083
I470	Paroxysmal tachycardia	Re-entry ventricular arrhythmia	0.3743	3.8367	2.5083
S510	Open wound of elbow and forearm	Open wound of elbow	2.3742	2.6265	2.5020
R36	Urethral discharge	Urethral discharge	0.8998	3.6011	2.4974
L8910	Pressure ulcer	Pressure ulcer of unspecified part of back	0.3422	3.8017	2.4755
B351	Dermatophytosis	Tinea unguium	2.0293	2.8529	2.4622
S498	Other and unspecified injuries of shoulder and upper arm	Other specified injuries of shoulder and upper arm	1.9850	2.8837	2.4599
R827	Other and unspecified abnormal findings in urine	Abnormal findings on microbiological examination of urine	0.3264	3.7855	2.4595
L740	Eccrine sweat disorders	Miliaria rubra	2.0426	2.8346	2.4581

L899	Pressure ulcer	Pressure ulcer of unspecified site	2.2952	2.5978	2.4491
L8991	Pressure ulcer	Pressure ulcer of unspecified site, stage 1	0.3112	3.7693	2.4437
H510	Other disorders of binocular movement	Palsy (spasm) of conjugate gaze	0.3112	3.7693	2.4437
R452	Symptoms and signs involving emotional state	Unhappiness	0.3112	3.7693	2.4437
R234	Other skin changes	Changes in skin texture	2.2839	2.5525	2.4200
H041	Disorders of lacrimal system	Other disorders of lacrimal gland	1.9021	2.8464	2.4024
D230	Other benign neoplasms of skin	Other benign neoplasm of skin of lip	0.2515	3.7062	2.3832
T174	Foreign body in respiratory tract	Foreign body in trachea	0.2374	3.6913	2.3686
L893	Pressure ulcer	Pressure ulcer of buttock	1.7743	2.8015	2.3219
G308	Alzheimer's disease	Other Alzheimer's disease	1.8469	2.7223	2.3091
N390	Other disorders of urinary system	Urinary tract infection, site not specified	2.2174	2.3337	2.2757
N429	Other and unspecified disorders of prostate	Disorder of prostate, unspecified	0.1424	3.5905	2.2719
H913	Other and unspecified hearing loss	Deaf nonspeaking, not elsewhere classified	0.1424	3.5905	2.2719
S798	Other and unspecified injuries of hip and thigh	Other specified injuries of hip and thigh	1.7663	2.7079	2.2656
Y9204	Place of occurrence of the external cause	Boarding-house as place	1.9981	2.2795	2.1411
S760	Injury of muscle, fascia and tendon at hip and thigh level	Injury of muscle, fascia and tendon of hip	1.5482	2.6440	2.1350
M513	Thoracic, thoracolum, and lumbosacral intvrt disc disorders	Oth thoracic, thrclm and lumbosacr intvrt disc degeneration	1.6339	2.5744	2.1327
Z604	Problems related to social environment	Social exclusion and rejection	1.6296	2.5701	2.1281
N318	Neuromuscular dysfunction of bladder, NEC	Other neuromuscular dysfunction of bladder	1.5039	2.6404	2.1143
R471	Speech disturbances, not elsewhere classified	Dysarthria and anarthria	1.9334	2.2641	2.1019

M750	Shoulder lesions	Adhesive capsulitis of shoulder	1.4963	2.5911	2.0829
H010	Other inflammation of eyelid	Blepharitis	1.4424	2.6245	2.0792
R64	Cachexia	Cachexia	1.4877	2.5828	2.0742
R4589	Symptoms and signs involving emotional state	Other symptoms and signs involving emotional state	1.8865	2.2280	2.0605
G479	Sleep disorders	Sleep disorder, unspecified	1.4800	2.5037	2.0260
R391	Oth and unsp symptoms and signs involving the GU sys	Other difficulties with micturition	1.4947	2.4601	2.0075
S325	Fracture of lumbar spine and pelvis	Fracture of pubis	1.8014	2.1819	1.9959
M6289	Other disorders of muscle	Other specified disorders of muscle	1.3853	2.5200	1.9951
G309	Alzheimer's disease	Alzheimer's disease, unspecified	1.8161	2.1541	1.9884
R600	Edema, not elsewhere classified	Localized edema	1.7904	2.1554	1.9767
E43	Unspecified severe protein-calorie malnutrition	Unspecified severe protein-calorie malnutrition	1.3403	2.5206	1.9763
N139	Obstructive and reflux uropathy	Obstructive and reflux uropathy, unspecified	1.3094	2.5418	1.9759
S0633	Intracranial injury	Contusion and laceration of cerebrum, unspecified	1.2762	2.5079	1.9423
S3091	Superfic inj abdomen, low back, pelvis and external genitals	Unspecified superficial injury of lower back and pelvis	1.3514	2.4449	1.9374
S011	Open wound of head	Open wound of eyelid and periocular area	1.7572	2.1069	1.9356
I958	Hypotension	Other hypotension	1.2559	2.4872	1.9221
B961	Oth bacterial agents as the cause of diseases classd elswhr	Klebsiella pneumoniae as the cause of diseases classd elswhr	1.7518	2.0787	1.9182
B952	Strep as the cause of diseases classified elsewhere	Enterococcus as the cause of diseases classified elsewhere	1.7348	2.0549	1.8979
R478	Speech disturbances, not elsewhere classified	Other speech disturbances	1.7102	2.0749	1.8964

M2555	Other joint disorder, not elsewhere classified	Pain in hip	1.7022	2.0670	1.8886
A4152	Other sepsis	Sepsis due to Pseudomonas	1.2994	2.3920	1.8849
H540	Blindness and low vision	Blindness, both eyes	1.2947	2.3872	1.8802
M2552	Other joint disorder, not elsewhere classified	Pain in elbow	1.1436	2.4335	1.8442
V556	Occupant of pk-up/van injured in collision w rail trn/veh	Pasngr in pk-up/van injured in clsn w rail trn/veh in traf	1.1330	2.4226	1.8334
Z436	Encounter for attention to artificial openings	Encounter for attn to oth artif openings of urinary tract	1.1330	2.4226	1.8334
S0130	Open wound of head	Unspecified open wound of ear	1.1484	2.3785	1.8138
S271	Injury of other and unspecified intrathoracic organs	Traumatic hemothorax	1.1951	2.3279	1.8039
R258	Abnormal involuntary movements	Other abnormal involuntary movements	1.1474	2.3258	1.7827
S069	Intracranial injury	Unspecified intracranial injury	1.1317	2.3095	1.7668
R638	Symptoms and signs concerning food and fluid intake	Other symptoms and signs concerning food and fluid intake	1.5661	1.9578	1.7666
D044	Carcinoma in situ of skin	Carcinoma in situ of skin of scalp and neck	1.1537	2.2862	1.7625
S299	Other and unspecified injuries of thorax	Unspecified injury of thorax	1.5439	1.9681	1.7616
M706	Soft tissue disorders related to use, overuse and pressure	Trochanteric bursitis	1.0178	2.3745	1.7580
S800	Superficial injury of knee and lower leg	Contusion of knee	1.5271	1.9515	1.7447
N179	Acute kidney failure	Acute kidney failure, unspecified	1.6666	1.7949	1.7309
S400	Superficial injury of shoulder and upper arm	Contusion of shoulder and upper arm	1.5131	1.9242	1.7238
S0045	Superficial injury of head	Superficial foreign body of ear	0.9294	2.3657	1.7176
B958	Strep as the cause of diseases classified elsewhere	Unsp staphylococcus as the cause of diseases classd elswhr	1.0120	2.3000	1.7116
E854	Amyloidosis	Organ-limited amyloidosis	1.0065	2.2946	1.7060

S0005	Superficial injury of head	Superficial foreign body of scalp	1.5149	1.8789	1.7009
J189	Pneumonia, unspecified organism	Pneumonia, unspecified organism	1.6296	1.7707	1.7006
S4220	Fracture of shoulder and upper arm	Unspecified fracture of upper end of humerus	1.0876	2.2192	1.6960
L97	Non-pressure chronic ulcer of lower limb, NEC	Non-pressure chronic ulcer of lower limb, NEC	1.4963	1.8710	1.6879
A4151	Other sepsis	Sepsis due to Escherichia coli [E. coli]	1.4750	1.8819	1.6834
G934	Other disorders of brain	Other and unspecified encephalopathy	0.9858	2.2144	1.6506
M2547	Other joint disorder, not elsewhere classified	Effusion, ankle and foot	0.8540	2.2895	1.6415
C442	Other and unspecified malignant neoplasm of skin	Oth and unsp malig neoplasm skin/ ear and extrn auric canal	1.4019	1.8428	1.6283
I959	Hypotension	Hypotension, unspecified	1.5518	1.7017	1.6273
G251	Other extrapyramidal and movement disorders	Drug-induced tremor	0.8696	2.2249	1.6092
E876	Other disorders of fluid, electrolyte and acid-base balance	Hypokalemia	1.5274	1.6860	1.6072
F609	Specific personality disorders	Personality disorder, unspecified	0.7561	2.2867	1.6012
I878	Other disorders of veins	Other specified disorders of veins	0.9651	2.1416	1.5998
L039	Cellulitis and acute lymphangitis	Cellulitis and acute lymphangitis, unspecified	0.9345	2.1624	1.5990
S0081	Superficial injury of head	Abrasion of other part of head	1.4098	1.7750	1.5964
Z978	Presence of other devices	Presence of other specified devices	0.7429	2.2732	1.5878
S001	Superficial injury of head	Contusion of eyelid and periocular area	1.3740	1.7888	1.5866
C444	Other and unspecified malignant neoplasm of skin	Other and unsp malignant neoplasm of skin of scalp and neck	1.3775	1.7761	1.5817
J22	Unspecified acute lower respiratory infection	Unspecified acute lower respiratory infection	1.4938	1.6546	1.5748

M2551	Other joint disorder, not elsewhere classified	Pain in shoulder	1.3442	1.7632	1.5590
I619	Nontraumatic intracerebral hemorrhage	Nontraumatic intracerebral hemorrhage, unspecified	0.8875	2.1153	1.5520
I350	Nonrheumatic aortic valve disorders	Nonrheumatic aortic (valve) stenosis	1.3326	1.7329	1.5375
E1043	Type 1 diabetes mellitus	Type 1 diabetes w diabetic autonomic (poly)neuropathy	0.7415	2.1757	1.5284
Y9209	Place of occurrence of the external cause	Oth non-institutional residence as place	1.4563	1.6000	1.5284
Z636	Oth prob rel to prim support group, inc family circumstances	Dependent relative needing care at home	0.7400	2.1743	1.5269
M7966	Oth and unsp soft tissue disorders, not elsewhere classified	Pain in lower leg	1.2756	1.7480	1.5189
C434	Malignant melanoma of skin	Malignant melanoma of scalp and neck	0.7696	2.1238	1.5087
S7240	Fracture of femur	Unspecified fracture of lower end of femur	0.8083	2.0947	1.5074
V609	Occupant of hv veh injured in collision w ped/anml	Occup of hv veh injured in collision w ped/anml in traf	0.7975	2.0837	1.4963
E1142	Type 2 diabetes mellitus	Type 2 diabetes mellitus with diabetic polyneuropathy	1.2854	1.6918	1.4936
B023	Zoster [herpes zoster]	Zoster ocular disease	0.6366	2.1656	1.4809
C446	Other and unspecified malignant neoplasm of skin	Oth and unsp malig neoplasm skin/ upper limb, inc shoulder	1.2672	1.6842	1.4809
S202	Superficial injury of thorax	Contusion of thorax	1.2723	1.6743	1.4782
G122	Spinal muscular atrophy and related syndromes	Motor neuron disease	0.7348	2.0888	1.4738
Y9241	Place of occurrence of the external cause	Street and highway as place	1.2610	1.6592	1.4649
G832	Other paralytic syndromes	Monoplegia of upper limb	0.6729	2.1068	1.4600
D045	Carcinoma in situ of skin	Carcinoma in situ of skin of trunk	0.5429	2.1889	1.4591

M2506	Other joint disorder, not elsewhere classified	Hemarthrosis, knee	0.6719	2.1055	1.4586
S810	Open wound of knee and lower leg	Open wound of knee	1.2075	1.6930	1.4575
E1169	Type 2 diabetes mellitus	Type 2 diabetes mellitus with other specified complication	1.2152	1.6842	1.4565
Z9665	Presence of other functional implants	Presence of artificial knee joint	1.2024	1.6949	1.4563
I489	Atrial fibrillation and flutter	Unspecified atrial fibrillation and atrial flutter	1.3770	1.5310	1.4547
I509	Heart failure	Heart failure, unspecified	1.2057	1.6777	1.4486
C433	Malignant melanoma of skin	Malignant melanoma of other and unspecified parts of face	0.7090	2.0628	1.4481
E639	Other nutritional deficiencies	Nutritional deficiency, unspecified	0.6016	2.1303	1.4457
S9082	Superficial injury of ankle, foot and toes	Blister (nonthermal) of foot	0.6575	2.0912	1.4446
S469	Injury of muscle, fascia and tendon at shldr/up arm	Injury of unsp muscle, fascia and tendon at shldr/up arm	0.5999	2.1288	1.4443
B022	Zoster [herpes zoster]	Zoster with other nervous system involvement	0.6921	2.0456	1.4310
Z466	Encounter for fitting and adjustment of other devices	Encounter for fitting and adjustment of urinary device	1.1653	1.6651	1.4231
I691	Sequelae of cerebrovascular disease	Sequelae of nontraumatic intracerebral hemorrhage	0.5074	2.1527	1.4231
N281	Oth disorders of kidney and ureter, not elsewhere classified	Cyst of kidney, acquired	0.6806	2.0343	1.4197
C445	Other and unspecified malignant neoplasm of skin	Other and unspecified malignant neoplasm of skin of trunk	1.1796	1.6423	1.4176
G4020	Epilepsy and recurrent seizures	Local-rel symptc epi w complex partial seiz, not ntrct	0.6785	2.0321	1.4173
L989	Oth disorders of skin, subcu, not elsewhere classified	Disorder of the skin and subcutaneous tissue, unspecified	1.1613	1.6538	1.4149

M542	Dorsalgia	Cervicalgia	1.1537	1.6462	1.4073
K761	Other diseases of liver	Chronic passive congestion of liver	0.6200	2.0534	1.4068
V536	Occupant of pk-up/van injured pick-up truck, pk-up/van	Pasngr in pk-up/van injured pick-up truck, pk-up/van in traf	1.1477	1.6477	1.4056
E880	Other and unspecified metabolic disorders	Disorders of plasma-protein metabolism, NEC	1.2021	1.5982	1.4049
E213	Hyperparathyroidism and other disorders of parathyroid gland	Hyperparathyroidism, unspecified	0.4787	2.1238	1.3945
A400	Streptococcal sepsis	Sepsis due to streptococcus, group A	0.6497	2.0032	1.3888
S900	Superficial injury of ankle, foot and toes	Contusion of ankle	0.6360	1.9892	1.3747
J80	Acute respiratory distress syndrome	Acute respiratory distress syndrome	0.5146	2.0432	1.3589
T821	Complications of cardiac and vascular prosth dev/grft	Mechanical complication of cardiac electronic device	0.6146	1.9677	1.3535
R222	Localized swelling, mass and lump of skin, subcu	Localized swelling, mass and lump, trunk	0.4363	2.0809	1.3517
Z431	Encounter for attention to artificial openings	Encounter for attention to gastrostomy	0.6032	1.9564	1.3421
V551	Occupant of pk-up/van injured in collision w rail trn/veh	Pasngr in pk-up/van injured in clsn w rail trn/veh nontraf	0.6032	1.9564	1.3421
K8030	Cholelithiasis	Calculus of bile duct w cholangitis, unsp, w/o obstruction	0.5550	1.9877	1.3413
S7241	Fracture of femur	Unspecified condyle fracture of lower end of femur	0.4965	2.0246	1.3408
C851	Oth and unspecified types of non-Hodgkin lymphoma	Unspecified B-cell lymphoma	0.4167	2.0615	1.3324
C447	Other and unspecified malignant neoplasm of skin	Oth and unsp malignant neoplasm skin/ lower limb, inc hip	1.1115	1.5414	1.3321
S1223	Fracture of cervical vertebra and other parts of neck	Unsp traumatic spondylolisthesis of third cervical vertebra	0.4128	2.0572	1.3281

K626	Other diseases of anus and rectum	Ulcer of anus and rectum	0.3031	2.0942	1.3097
J156	Bacterial pneumonia, not elsewhere classified	Pneumonia due to other aerobic Gram-negative bacteria	0.4523	1.9800	1.2964
I38	Endocarditis, valve unspecified	Endocarditis, valve unspecified	0.5092	1.9413	1.2953
K921	Other diseases of digestive system	Melena	1.0487	1.5269	1.2950
T172	Foreign body in respiratory tract	Foreign body in pharynx	0.3743	2.0181	1.2892
Z450	Encounter for adjustment and management of implanted device	Encounter for adjustment and management of cardiac device	1.0346	1.5160	1.2826
S898	Other and unspecified injuries of lower leg	Other specified injuries of lower leg	0.4959	1.9280	1.2820
I618	Nontraumatic intracerebral hemorrhage	Other nontraumatic intracerebral hemorrhage	0.3639	2.0082	1.2795
E1141	Type 2 diabetes mellitus	Type 2 diabetes mellitus with diabetic mononeuropathy	0.4929	1.9250	1.2790
F309	Manic episode	Manic episode, unspecified	0.2570	2.0481	1.2638
I501	Heart failure	Left ventricular failure	1.0289	1.4690	1.2548
L570	Skin changes due to chronic expsr to nonionizing radiation	Actinic keratosis	1.0371	1.4598	1.2539
H440	Disorders of globe	Purulent endophthalmitis	0.2445	2.0352	1.2513
G472	Sleep disorders	Circadian rhythm sleep disorders	0.4587	1.8909	1.2447
T447	Drugs primarily affecting the autonomic nervous system	Beta-adrenoreceptor antagonists	0.4568	1.8887	1.2427
M2553	Other joint disorder, not elsewhere classified	Pain in wrist	0.3235	1.9671	1.2387
R092	Oth symptoms and signs involving the circ and resp sys	Respiratory arrest	0.4460	1.8782	1.2323
Z854	Personal history of malignant neoplasm	Personal history of malignant neoplasm of genital organs	0.2215	2.0116	1.2279
R066	Abnormalities of breathing	Hiccough	0.3819	1.9094	1.2258

G4010	Epilepsy and recurrent seizures	Local-rel symptc epi w simple partial seiz, not ntrct	0.3798	1.9069	1.2235
Z951	Presence of cardiac and vascular implants and grafts	Presence of aortocoronary bypass graft	0.9847	1.4469	1.2226
S323	Fracture of lumbar spine and pelvis	Fracture of ilium	0.3023	1.9456	1.2173
B957	Strep as the cause of diseases classified elsewhere	Oth staphylococcus as the cause of diseases classd elswhr	0.9712	1.4490	1.2173
S468	Injury of muscle, fascia and tendon at shldr/up arm	Injury of musc/fasc/tend at shoulder and upper arm level	0.4311	1.8627	1.2170
R008	Abnormalities of heart beat	Other abnormalities of heart beat	0.3729	1.9000	1.2167
S2205	Fracture of rib(s), sternum and thoracic spine	Fracture of T5-T6 vertebra	0.3646	1.8919	1.2087
D473	Oth neoplm of uncrt behav of lymphoid, hematpoetc & rel tiss	Essential (hemorrhagic) thrombocythemia	0.2919	1.9354	1.2069
K2951	Gastritis and duodenitis	Unspecified chronic gastritis with bleeding	0.3584	1.8851	1.2018
Z8671	Personal history of certain other diseases	Personal history of venous thrombosis and embolism	0.1923	1.9825	1.1988
K521	Other and unsp noninfective gastroenteritis and colitis	Toxic gastroenteritis and colitis	0.9662	1.4139	1.1963
G500	Disorders of trigeminal nerve	Trigeminal neuralgia	0.3264	1.8527	1.1697
S225	Fracture of rib(s), sternum and thoracic spine	Flail chest	0.2531	1.8958	1.1675
S6230	Fracture at wrist and hand level	Unspecified fracture of other metacarpal bone	0.3243	1.8505	1.1675
J449	Other chronic obstructive pulmonary disease	Chronic obstructive pulmonary disease, unspecified	0.9377	1.3853	1.1675
E1161	Type 2 diabetes mellitus	Type 2 diabetes mellitus with diabetic arthropathy	0.3185	1.8451	1.1619
H920	Otalgia and effusion of ear	Otalgia	0.3155	1.8418	1.1588

E1151	Type 2 diabetes mellitus	Type 2 diabetes w diabetic peripheral angiopath w/o gangrene	0.9018	1.3795	1.1477
I472	Paroxysmal tachycardia	Ventricular tachycardia	0.8637	1.4009	1.1417
R58	Hemorrhage, not elsewhere classified	Hemorrhage, not elsewhere classified	0.2964	1.8226	1.1394
S3205	Fracture of lumbar spine and pelvis	Fracture of fifth lumbar vertebra	0.1319	1.9213	1.1378
T887	Oth complications of surgical and medical care, NEC	Unspecified adverse effect of drug or medicament	0.2054	1.8481	1.1200
G938	Other disorders of brain	Other specified disorders of brain	0.2761	1.8027	1.1194
L82	Seborrheic keratosis	Seborrheic keratosis	0.8272	1.3843	1.1158
I609	Nontraumatic subarachnoid hemorrhage	Nontraumatic subarachnoid hemorrhage, unspecified	0.2546	1.7807	1.0979
K430	Ventral hernia	Incisional hernia with obstruction, without gangrene	0.0889	1.8781	1.0949
M2441	Other specific joint derangements	Recurrent dislocation, shoulder	0.0853	1.8743	1.0909
H811	Disorders of vestibular function	Benign paroxysmal vertigo	0.7798	1.3719	1.0869
F402	Phobic anxiety disorders	Specific (isolated) phobias	0.0714	1.8605	1.0777
K056	Gingivitis and periodontal diseases	Periodontal disease, unspecified	0.1579	1.8004	1.0726
Z603	Problems related to social environment	Acculturation difficulty	0.1544	1.7971	1.0692
R7989	Other abnormal findings of blood chemistry	Other specified abnormal findings of blood chemistry	0.0620	1.8513	1.0682
R090	Oth symptoms and signs involving the circ and resp sys	Asphyxia and hypoxemia	0.7524	1.3379	1.0561
E119	Type 2 diabetes mellitus	Type 2 diabetes mellitus without complications	0.9704	1.1372	1.0547
E1134	Type 2 diabetes mellitus	Type 2 diabetes w severe nonprlf diabetic retinopathy	0.7110	1.2790	1.0054
S0151	Open wound of head	Laceration of lip and oral cavity without foreign body	0.6413	1.2390	0.9517
H353	Other retinal disorders	Degeneration of macula and posterior pole	0.6270	1.2060	0.9270

R040	Hemorrhage from respiratory passages	Epistaxis	0.6114	1.1262	0.8771
E611	Deficiency of other nutrient elements	Iron deficiency	0.5619	1.1291	0.8557
T827	Complications of cardiac and vascular prosth dev/grft	Infect/inflm reaction due to oth cardi/vasc dev/implnt/grft	0.4492	1.1165	0.7975
L309	Other and unspecified dermatitis	Dermatitis, unspecified	0.3954	1.0285	0.7246
I978	Intraop and postproc comp and disorders of circ sys, NEC	Oth intraop and postproc comp and disord of circ sys, NEC	0.3736	1.0149	0.7075
M751	Shoulder lesions	Rotator cuff tear or rupture, not specified as traumatic	0.4048	0.9780	0.7021
J47	Bronchiectasis	Bronchiectasis	0.3228	1.0399	0.6981
H258	Age-related cataract	Other age-related cataract	0.3723	0.9907	0.6936
K567	Paralytic ileus and intestinal obstruction without hernia	Ileus, unspecified	0.3743	0.9787	0.6881
E872	Other disorders of fluid, electrolyte and acid-base balance	Acidosis	0.3688	0.9532	0.6719
R91	Abnormal findings on diagnostic imaging of lung	Abnormal findings on diagnostic imaging of lung	0.2761	1.0047	0.6575
Z530	Persons encntr hlth serv for spec proc & trtmt, not crd out	Proc/trtmt not carried out because of contraindication	0.3436	0.9478	0.6575
V641	Occupant of hv veh injured in collision w hv veh	Passenger in hv veh injured in collision w hv veh nontraf	0.3436	0.9474	0.6570
R11	Nausea and vomiting	Nausea and vomiting	0.5306	0.7491	0.6413
R068	Abnormalities of breathing	Other abnormalities of breathing	0.3185	0.9290	0.6355
J9691	Respiratory failure, not elsewhere classified	Respiratory failure, unspecified with hypoxia	0.2508	0.9798	0.6329
T813	Complications of procedures, not elsewhere classified	Disruption of wound, not elsewhere classified	0.2429	0.9286	0.6010
T818	Complications of procedures, not elsewhere classified	Other complications of procedures, not elsewhere classified	0.1989	0.8242	0.5241

S5251	Fracture of forearm	Fracture of radial styloid process	0.1781	0.8185	0.5116
D696	Purpura and other hemorrhagic conditions	Thrombocytopenia, unspecified	0.1310	0.7793	0.4688
N185	Chronic kidney disease (CKD)	Chronic kidney disease, stage 5	0.1310	0.7636	0.4600
K047	Diseases of pulp and periapical tissues	Periapical abscess without sinus	-1.2623	-0.1381	-0.6578
K621	Other diseases of anus and rectum	Rectal polyp	-1.3471	-0.1767	-0.7133
K353	Acute appendicitis	Acute appendicitis with localized peritonitis	-1.6928	-0.1720	-0.8510
R102	Abdominal and pelvic pain	Pelvic and perineal pain	-1.7373	-0.2194	-0.8989
E668	Overweight and obesity	Other obesity	-1.7487	-0.2307	-0.9113
N939	Other abnormal uterine and vaginal bleeding	Abnormal uterine and vaginal bleeding, unspecified	-1.9878	-0.3510	-1.0759
W50	Acc hit, strk, kick, twist, bite or scratch by another prsn	Acc hit, strk, kick, twist, bite or scratch by another prsn	-2.1120	-0.3271	-1.1056
Y927	Place of occurrence of the external cause	Farm as the place of occurrence of the external cause	-2.0250	-0.3916	-1.1147
K429	Umbilical hernia	Umbilical hernia without obstruction or gangrene	-2.0326	-0.5142	-1.1940
N736	Other female pelvic inflammatory diseases	Female pelvic peritoneal adhesions (postinfective)	-2.4651	-0.6773	-1.4567
N23	Unspecified renal colic	Unspecified renal colic	-2.6037	-0.6236	-1.4740
T432	Psychotropic drugs, not elsewhere classified	And unsp antidepressants	-2.8824	-0.6330	-1.5750
J342	Other and unspecified disorders of nose and nasal sinuses	Deviated nasal septum	-2.9188	-0.6812	-1.6246
N832	Noninflammatory disord of ovary, fallop and broad ligament	Other and unspecified ovarian cysts	-2.7489	-0.7765	-1.6246
W03	Oth fall on same level due to collision with another person	Oth fall on same level due to collision with another person	-3.3524	-0.6972	-1.7720
K610	Abscess of anal and rectal regions	Anal abscess	-4.1997	-0.8164	-2.1037

J039	Acute tonsillitis	Acute tonsillitis, unspecified	-4.5099	-1.1394	-2.4191
H659	Nonsuppurative otitis media	Unspecified nonsuppurative otitis media	-4.6052	-1.2006	-2.4889
H669	Suppurative and unspecified otitis media	Otitis media, unspecified	-4.6052	-1.2553	-2.5383
N840	Polyp of female genital tract	Polyp of corpus uteri	-4.7105	-1.2730	-2.5639
T510	Toxic effect of alcohol	Toxic effect of ethanol	-6.9078	-1.1648	-2.8824

Supplementary Table 11. PD comorbid diseases that are also linked to genes within the PD-causal network.

genes	PPI level	MeSH ID	MeSH Heading	Disease code	descShort	log10_or	log10_or_lower	log10_or_upper
PPARGC1A	L3	D003924	Diabetes Mellitus, Type 2	E1143	Type 2 diabetes w diabetic autonomic (poly)neuropathy	2.6378	2.5037	2.7682
PPARGC1A	L3	D003924	Diabetes Mellitus, Type 2	E1142	Type 2 diabetes mellitus with diabetic polyneuropathy	1.4936	1.2854	1.6918
PPARGC1A	L3	D003924	Diabetes Mellitus, Type 2	E1169	Type 2 diabetes mellitus with other specified complication	1.4565	1.2152	1.6842
PPARGC1A	L3	D003924	Diabetes Mellitus, Type 2	E1141	Type 2 diabetes mellitus with diabetic mononeuropathy	1.2790	0.4929	1.9250
PPARGC1A	L3	D003924	Diabetes Mellitus, Type 2	E1161	Type 2 diabetes mellitus with diabetic arthropathy	1.1619	0.3185	1.8451
PPARGC1A	L3	D003924	Diabetes Mellitus, Type 2	E1151	Type 2 diabetes w diabetic peripheral angiopath w/o gangrene	1.1477	0.9018	1.3795
PPARGC1A	L3	D003924	Diabetes Mellitus, Type 2	E119	Type 2 diabetes mellitus without complications	1.0547	0.9704	1.1372
PPARGC1A	L3	D003924	Diabetes Mellitus, Type 2	E1134	Type 2 diabetes w severe nonprlf diabetic retinopathy	1.0054	0.7110	1.2790
PPARGC1A	L3	D006333	Heart Failure	I509	Heart failure, unspecified	1.4486	1.2057	1.6777
NRG1	L2	D006333	Heart Failure	I509	Heart failure, unspecified	1.4486	1.2057	1.6777
KAT8	L0	D006333	Heart Failure	I509	Heart failure, unspecified	1.4486	1.2057	1.6777

PPARGC1A	L3	D006333	Heart Failure	I501	Left ventricular failure	1.2548	1.0289	1.4690
NRG1	L2	D006333	Heart Failure	I501	Left ventricular failure	1.2548	1.0289	1.4690
KAT8	L0	D006333	Heart Failure	I501	Left ventricular failure	1.2548	1.0289	1.4690
SIRT3	L2	D009765	Obesity	E668	Other obesity	-0.9113	-1.7487	-0.2307
ATM	L1	D008545	Melanoma	C434	Malignant melanoma of scalp and neck	1.5087	0.7696	2.1238
RAF1,GAB2,DCC	L3	D008545	Melanoma	C434	Malignant melanoma of scalp and neck	1.5087	0.7696	2.1238
ATM	L1	D008545	Melanoma	C433	Malignant melanoma of other and unspecified parts of face	1.4481	0.7090	2.0628
RAF1,GAB2,DCC	L3	D008545	Melanoma	C433	Malignant melanoma of other and unspecified parts of face	1.4481	0.7090	2.0628
RAF1	L3	C562393	Melanoma, Cutaneous Malignant	C434	Malignant melanoma of scalp and neck	1.5087	0.7696	2.1238
RAF1	L3	C562393	Melanoma, Cutaneous Malignant	C433	Malignant melanoma of other and unspecified parts of face	1.4481	0.7090	2.0628
ERBB2	L3	D002280	Carcinoma, Basal Cell	C442	Oth and unsp malig neoplasm skin/ ear and extrn auric canal	1.6283	1.4019	1.8428
ERBB2	L3	D002280	Carcinoma, Basal Cell	C444	Other and unsp malignant neoplasm of skin of scalp and neck	1.5817	1.3775	1.7761
ERBB2	L3	D002280	Carcinoma, Basal Cell	C446	Oth and unsp malig neoplasm skin/ upper limb, inc shoulder	1.4809	1.2672	1.6842
ERBB2	L3	D002280	Carcinoma, Basal Cell	C445	Other and unspecified malignant neoplasm of skin of trunk	1.4176	1.1796	1.6423
ERBB2	L3	D002280	Carcinoma, Basal Cell	C447	Oth and unsp malignant neoplasm skin/ lower limb, inc hip	1.3321	1.1115	1.5414

KAT8	L0	D012640	Seizures	G4020	Local-rel symptc epi w complex partial seiz, not ntrct	1.4173	0.6785	2.0321
KAT8	L0	D012640	Seizures	G4010	Local-rel symptc epi w simple partial seiz, not ntrct	1.2235	0.3798	1.9069
CDH3	L3	D010048	Ovarian Cysts	N832	Other and unspecified ovarian cysts	-1.6246	-2.7489	-0.7765
CDH3	L3	D005128	Eye Diseases	H440	Purulent endophthalmitis	1.2513	0.2445	2.0352
KDR	L2	D002543	Cerebral Hemorrhage	I619	Nontraumatic intracerebral hemorrhage, unspecified	1.5520	0.8875	2.1153
KDR	L2	D002543	Cerebral Hemorrhage	I618	Other nontraumatic intracerebral hemorrhage	1.2795	0.3639	2.0082
SETD1A	L3	D004827	Epilepsy	G4020	Local-rel symptc epi w complex partial seiz, not ntrct	1.4173	0.6785	2.0321
SETD1A	L3	D004827	Epilepsy	G4010	Local-rel symptc epi w simple partial seiz, not ntrct	1.2235	0.3798	1.9069
GPNMB	L0	D058186	Acute Kidney Injury	N179	Acute kidney failure, unspecified	1.7309	1.6666	1.7949

Supplementary Table 12. PD-causal network genes mapped to MeSH terms.

Gene	MeSH ID	MeSH Heading
ATM	D001260	Ataxia Telangiectasia
RAF1	D009634	Noonan Syndrome
RAF1	D009634	Turner Syndrome, Male
RAF1	D009634	Female Pseudo-Turner Syndrome
RAF1	D009634	Noonan Syndrome 1
RAF1	C548083	Noonan Syndrome 5
ATM	D007938	Leukemia
ATM	D008223	Lymphoma
FGFR1	D000168	Syndrome, Apert

FGFR1	D000168	Saethre-Chotzen Syndrome
FGFR1	D000168	Pfeiffer Syndrome
FGFR1	D000168	Acrocephalosyndactylia
FGFR1	D000168	Apert-Crouzon Disease
ATM	D020522	Lymphoma, Lymphocytic, Diffuse, Poorly-Differentiated
ATM	D020522	Lymphoma, Mantle-Cell
ATM	D020522	Lymphoma, Lymphocytic, Intermediate
ERBB2	D001943	Malignant Neoplasm of Breast
ERBB2	D001943	Breast Carcinoma
ERBB2	D001943	Mammary Neoplasms, Human
ERBB2	D001943	Breast Neoplasms
ERBB2	D001943	Mammary Carcinoma, Human
NPHS1	C535761	Finnish congenital nephrosis
NPHS1	C535761	Nephrotic Syndrome, Type 3
NPHS1	C535761	Nephrotic Syndrome, Idiopathic
NPHS1	C535761	Nephrosis, congenital
AAAS	C536008	Glucocorticoid deficiency and achalasia
AAAS	C536008	Achalasia Addisonianism Alacrimia syndrome
RAF1	D044542	LEOPARD Syndrome
RAF1	D044542	Leopard Syndrome 1
FGFR1	C564484	Holoprosencephaly, Ectrodactyly, and Bilateral Cleft Lip-Palate
KANSL1	C566476	Chromosome 17q21.31 Deletion Syndrome
CDH3	C536190	Ectodermal dysplasia, ectrodactyly, and macular dystrophy
FGFR1	C535736	Encephalocraniocutaneous lipomatosis
FGFR1	C536050	Osteoglophonic dwarfism
TXNL4A	C537411	Burn-Mckeown syndrome
FGFR1	D017436	Kallmann Syndrome

FGFR1	D017436	Kallmann Syndrome 1
FGFR1	D017436	Kallmann Syndrome 2
FGFR1	D017436	Kallmann Syndrome, Type 3, Recessive
FGFR1	C537559	Jackson-Weiss syndrome
ATM	D001943	Malignant Neoplasm of Breast
ATM	D001943	Breast Carcinoma
ATM	D001943	Mammary Neoplasms, Human
ATM	D001943	Breast Neoplasms
ATM	D001943	Mammary Carcinoma, Human
ATM	D015451	Leukemia, Lymphocytic, Chronic, B-Cell
ATM	D015451	Chronic Lymphocytic Leukemia
CDH3	C537698	Juvenile macular degeneration and hypotrichosis
CDH3	C537698	Hypotrichosis, congenital, with juvenile macular dystrophy
DCC	D015179	Colorectal Carcinoma
DCC	D015179	Colorectal Neoplasms
ERBB2	D013274	Gastric Cancer
ERBB2	D013274	Stomach Neoplasms
ERBB2	D013274	Gastric Cancer, Familial Diffuse
ERBB2	D010051	Ovarian Neoplasms
ERBB2	D010051	Neoplasm, Ovarian
ERBB2	D010051	Ovarian Cancer
FGFR1	D003398	Craniosynostosis
FGFR1	D003398	Craniosynostoses
FGFR1	D003398	Acrocephaly
FGFR1	D003398	Brachycephaly
FGFR1	D003398	Scaphocephaly
FGFR1	D003398	Trigonocephaly

FGFR1	D003398	Synostotic Posterior Plagiocephaly
FGFR1	D003398	Synostosis, Metopic
FGFR1	D003398	Synostotic Anterior Plagiocephaly
FGFR1	D003398	Craniosynostosis, Type 1
KDR	C535860	Hemangioma, capillary infantile
RAF1	C537117	LEOPARD syndrome, 2
SFTPD	D029424	Chronic Obstructive Airway Disease
SFTPD	D029424	Pulmonary Disease, Chronic Obstructive
SFTPD	D029424	Chronic Airflow Obstruction
CHMP4B	C535343	Cataract, posterior polar, 3
ATM	D001749	Cancer of Bladder
ATM	D001749	Bladder Neoplasm
ATM	D001749	Urinary Bladder Neoplasms
SETD1A	D012559	Schizophrenia
FGFR1	D003866	Depression, Endogenous
FGFR1	D003866	Disorder, Depressive
FGFR1	D003866	Depressive Disorder
FGFR1	D003866	Melancholia
FGFR1	D003866	Unipolar Depression
FGFR1	D003866	Depressive Syndrome
FGFR1	D003866	Depression, Neurotic
WNT3	C536498	Tetra-amelia autosomal recessive
FGFR1	C562951	Craniosynostosis, Metopic
FGFR1	C562951	Trigonocephaly, Nonsyndromic
ATM	D005910	Glioma
ATM	D005910	Gliomas, Mixed
ATM	D005910	Malignant Glioma

ATM	D013274	Gastric Cancer
ATM	D013274	Stomach Neoplasms
ATM	D013274	Gastric Cancer, Familial Diffuse
ERBB2	D009362	Neoplasm Metastasis
FGFR1	D001943	Malignant Neoplasm of Breast
FGFR1	D001943	Breast Carcinoma
FGFR1	D001943	Mammary Neoplasms, Human
FGFR1	D001943	Breast Neoplasms
FGFR1	D001943	Mammary Carcinoma, Human
KDR	D015179	Colorectal Carcinoma
KDR	D015179	Colorectal Neoplasms
POLE	D015179	Colorectal Carcinoma
POLE	D015179	Colorectal Neoplasms
PTK2	D006528	Carcinoma, Hepatocellular
PPARGC1A	D003924	Diabetes Mellitus, Type 2
PPARGC1A	D003924	Diabetes Mellitus, Non-Insulin-Dependent
PPARGC1A	D006333	Heart Failure
PPARGC1A	D006333	Heart Failure, Congestive
PPARGC1A	D006333	Left-Sided Heart Failure
PPARGC1A	D006333	Heart Failure, Right-Sided
PPARGC1A	D006333	Myocardial Failure
PPARGC1A	D006333	Heart Decompensation
SIRT3	D009765	Obesity
KDR	D006394	Hemangiosarcoma
GPNMB	D001943	Malignant Neoplasm of Breast
GPNMB	D001943	Breast Carcinoma
GPNMB	D001943	Mammary Neoplasms, Human

GPNMB	D001943	Breast Neoplasms
GPNMB	D001943	Mammary Carcinoma, Human
PTK2	D005909	Glioblastoma
PTK2	D005909	Giant Cell Glioblastoma
PTK2	D005909	Glioblastoma Multiforme
PPARGC1A	D009203	Myocardial Infarction
TTC19	C565128	Mitochondrial Complex III Deficiency
ATM	D008527	Medulloblastoma
ATM	D008527	Medullomyoblastoma
ATM	D008527	Childhood Medulloblastoma
ATM	D008527	Adult Medulloblastoma
ATM	D008527	Desmoplastic Medulloblastoma
ATM	D008527	Melanocytic Medulloblastoma
DCC	D004938	Esophageal Neoplasms
DCC	D004938	Esophageal Cancer
ATM	D008545	Melanoma
ATM	D009369	Malignant Neoplasms
ATM	D009369	Neoplasms
ATM	D009369	Benign Neoplasm
ATM	D015461	Leukemia, T-Cell, Chronic
ATM	D015461	T-Cell Prolymphocytic Leukemia
ATM	D015461	Leukemia, Prolymphocytic, T-Cell
CD38	D008106	Liver Cirrhosis, Experimental
CD38	D017202	Myocardial Ischemia
ERBB2	D002289	Carcinoma, Non-Small-Cell Lung
ERBB2	D002289	Non Small Cell Lung Carcinoma
ERBB2	D008325	Mammary Neoplasms, Experimental

ERBB2	D000077192	Adenocarcinoma of Lung
FGFR1	C562785	Idiopathic Hypogonadotropic Hypogonadism
GAK	D010300	Parkinson Disease
NRG1	D001714	Bipolar Disorder
NRG1	D001714	Depression, Bipolar
NRG1	D001714	Manic Disorder
NRG1	D001714	Mania
NRG1	D006627	Hirschsprung Disease
NRG1	D006627	Aganglionosis, Colonic
NRG1	D006627	Aganglionosis, Rectosigmoid Colon
NRG1	D006627	Congenital Intestinal Aganglionosis
NRG1	D012559	Schizophrenia
KDR	D015427	Reperfusion Injury
POLE	D016889	Endometrial Neoplasms
POLE	D016889	Endometrial Carcinoma
PTK2	D008325	Mammary Neoplasms, Experimental
RAF1	D002312	Cardiomyopathy, Hypertrophic
RAF1	D002312	Hypertrophic Cardiomyopathy
RAF1	D002312	Cardiomyopathy, Hypertrophic Obstructive
RAF1	D008545	Melanoma
PPARGC1A	D003921	Alloxan Diabetes
PPARGC1A	D003921	Diabetes Mellitus, Experimental
PPARGC1A	D003921	Streptozotocin Diabetes
PPARGC1A	D000690	Amyotrophic Lateral Sclerosis
PPARGC1A	D000690	Amyotrophic Lateral Sclerosis With Dementia
PPARGC1A	D000690	Amyotrophic Lateral Sclerosis, Guam Form
FGFR1	D001254	Astrocytoma

FGFR1	D001254	Subependymal Giant Cell Astrocytoma
FGFR1	D001254	Juvenile Pilocytic Astrocytoma
FGFR1	D001254	Astrocytoma, Grade II
FGFR1	D001254	Astrocytoma, Anaplastic
FGFR1	D001254	Astrocytoma, Protoplasmic
FGFR1	D001254	Astrocytoma, Gemistocytic
FGFR1	D001254	Fibrillary Astrocytoma
FGFR1	D001254	Pilocytic Astrocytoma
FGFR1	D001254	Childhood Cerebral Astrocytoma
FGFR1	D001254	Oligoastrocytoma, Mixed
FGFR1	D001254	Cerebral Astrocytoma
FGFR1	D001254	Intracranial Astrocytoma
FGFR1	D001254	Grade I Astrocytoma
FGFR1	D002972	Cleft Palate
FGFR1	D002972	Cleft Palate, Isolated
KANSL1	D008607	Idiocy
KANSL1	D008607	Mental Retardation, Psychosocial
KANSL1	D008607	Deficiency, Mental
KANSL1	D008607	Intellectual Disability
RAF1	C562393	Familial Atypical Mole-Malignant Melanoma Syndrome
RAF1	C562393	Melanoma, Cutaneous Malignant
XRCC4	D004392	Dwarfism
DCC	D012559	Schizophrenia
DCC	D061085	Corpus Callosum, Agenesis Of
DCC	D061085	Agenesis of Corpus Callosum
DCC	D061085	Corpus Callosum Malformation
FGFR1	D002971	Cleft Lip

RAF1	D000077192	Adenocarcinoma of Lung
XRCC4	C537404	Pituitary dwarfism 1
XRCC4	C537404	Pituitary Dwarfism I
ATM	D004421	Dystonia
ATM	D004421	Dystonia, Paroxysmal
ATM	D004421	Dystonia, Diurnal
ATM	D004421	Dystonia, Limb
ATM	D007153	Antibody Deficiency Syndrome
ATM	D007153	Immunologic Deficiency Syndromes
FGFR1	D012559	Schizophrenia
FGFR1	D025962	Septo-Optic Dysplasia
VTI1A	D015179	Colorectal Carcinoma
VTI1A	D015179	Colorectal Neoplasms
DCC	D008607	Idiocy
DCC	D008607	Mental Retardation, Psychosocial
DCC	D008607	Deficiency, Mental
DCC	D008607	Intellectual Disability
FGFR1	D000848	Hypodontia
FGFR1	D000848	Anodontia
FGFR1	D000848	Tooth Agenesis, Familial
NPHS1	D011507	Proteinuria
NUP88	C536647	Pena Shokeir syndrome, type 1
NUP88	C536647	Pena-Shokeir Syndrome, Type I
RAF1	D002311	Cardiomyopathy, Dilated
RAF1	D002311	Cardiomyopathy, Familial Idiopathic
ATM	D002294	Carcinoma, Squamous Cell
ATM	D000077192	Adenocarcinoma of Lung

ATM	C562840	Breast Cancer, Familial
ATM	D010190	Pancreatic Neoplasm
ATM	D010190	Pancreatic Neoplasms
ATM	D010190	Pancreatic Cancer
ATM	D011471	Prostatic Neoplasms
ATM	D011471	Prostate Cancer
ATM	C566865	Ataxia-Telangiectasia Variant
CDH3	D003093	Ulcerative Colitis
CDH3	D003093	Colitis, Ulcerative
ERBB2	D000230	Adenocarcinoma
ERBB2	D000230	Adenocarcinoma, Basal Cell
ERBB2	D000230	Adenocarcinoma, Oxyphilic
ERBB2	D000230	Carcinoma, Cribriform
ERBB2	D000230	Carcinoma, Granular Cell
ERBB2	D000230	Adenocarcinoma, Tubular
ERBB2	D009369	Malignant Neoplasms
ERBB2	D009369	Neoplasms
ERBB2	D009369	Benign Neoplasm
ERBB2	D003110	Cancer of Colon
ERBB2	D003110	Colonic Neoplasms
ERBB2	D002292	Renal Cell Carcinoma
ERBB2	D002292	Carcinoma, Renal Cell
ERBB2	D002292	Carcinoma, Hypernephroid
ERBB2	D002292	Chromophobe Renal Cell Carcinoma
ERBB2	D002292	Sarcomatoid Renal Cell Carcinoma
ERBB2	D002292	Collecting Duct Carcinoma of the Kidney
ERBB2	D002292	Papillary Renal Cell Carcinoma

ERBB2	D004938	Esophageal Neoplasms
ERBB2	D004938	Esophageal Cancer
ERBB2	D005910	Glioma
ERBB2	D005910	Gliomas, Mixed
ERBB2	D005910	Malignant Glioma
ERBB2	D008175	Lung Neoplasms
ERBB2	D008175	Lung Cancer
ERBB2	D011471	Prostatic Neoplasms
ERBB2	D011471	Prostate Cancer
ERBB2	D005706	Gallbladder Neoplasms
ERBB2	D005706	Neoplasm, Gallbladder
ERBB2	D005706	Cancer of Gallbladder
ERBB2	D018281	Cholangiocarcinoma
ERBB2	D018281	Intrahepatic Cholangiocarcinoma
ERBB2	D018281	Extrahepatic Cholangiocarcinoma
ERBB2	D063646	Carcinogenesis
ERBB2	D005770	Gastrointestinal Neoplasms
ERBB2	D005770	Gastrointestinal Cancer
ERBB2	D018270	Mammary Ductal Carcinoma
ERBB2	D018270	Carcinoma, Ductal, Breast
ERBB2	D018270	Carcinoma, Invasive Ductal, Breast
ERBB2	D000077216	Carcinoma, Ovarian Epithelial
FGFR1	D009369	Malignant Neoplasms
FGFR1	D009369	Neoplasms
FGFR1	D009369	Benign Neoplasm
FGFR1	D002289	Carcinoma, Non-Small-Cell Lung
FGFR1	D002289	Non Small Cell Lung Carcinoma

FGFR1	D002294	Carcinoma, Squamous Cell
FGFR1	D009196	Myeloproliferative Disorders
FGFR1	D008175	Lung Neoplasms
FGFR1	D008175	Lung Cancer
FGFR1	D016142	Arhinencephaly
FGFR1	D016142	Holoprosencephaly
FGFR1	D016142	Lobar Holoprosencephaly
FGFR1	D016142	Alobar Holoprosencephaly
FGFR1	D016142	Semilobar Holoprosencephaly
NRG1	D001943	Malignant Neoplasm of Breast
NRG1	D001943	Breast Carcinoma
NRG1	D001943	Mammary Neoplasms, Human
NRG1	D001943	Breast Neoplasms
NRG1	D001943	Mammary Carcinoma, Human
NRG1	D006333	Heart Failure
NRG1	D006333	Heart Failure, Congestive
NRG1	D006333	Left-Sided Heart Failure
NRG1	D006333	Heart Failure, Right-Sided
NRG1	D006333	Myocardial Failure
NRG1	D006333	Heart Decompensation
NRG1	D008175	Lung Neoplasms
NRG1	D008175	Lung Cancer
KDR	D001943	Malignant Neoplasm of Breast
KDR	D001943	Breast Carcinoma
KDR	D001943	Mammary Neoplasms, Human
KDR	D001943	Breast Neoplasms
KDR	D001943	Mammary Carcinoma, Human

KDR	D009369	Malignant Neoplasms
KDR	D009369	Neoplasms
KDR	D009369	Benign Neoplasm
KDR	D002289	Carcinoma, Non-Small-Cell Lung
KDR	D002289	Non Small Cell Lung Carcinoma
KDR	D002292	Renal Cell Carcinoma
KDR	D002292	Carcinoma, Renal Cell
KDR	D002292	Carcinoma, Hypernephroid
KDR	D002292	Chromophobe Renal Cell Carcinoma
KDR	D002292	Sarcomatoid Renal Cell Carcinoma
KDR	D002292	Collecting Duct Carcinoma of the Kidney
KDR	D002292	Papillary Renal Cell Carcinoma
KDR	D010190	Pancreatic Neoplasm
KDR	D010190	Pancreatic Neoplasms
KDR	D010190	Pancreatic Cancer
NPHS1	D005921	Glomerulonephritis
NPHS1	D005921	Bright Disease
NPHS1	D005923	Glomerulosclerosis, Focal
NPHS1	D005923	Glomerulosclerosis, Focal Segmental
NPHS1	D005923	Hyalinosis, Segmental Glomerular
NPHS1	C536404	Nephrotic syndrome, idiopathic, steroid-resistant
NPHS1	C536404	Nephrotic syndrome, steroid-resistant, autosomal recessive
PTK2	D001281	Atrial Fibrillation
PTK2	D001281	Atrial Fibrillation, Paroxysmal
PTK2	D001281	Atrial Fibrillation, Persistent
PTK2	D001281	Atrial Fibrillation, Familial
RAF1	D001943	Malignant Neoplasm of Breast

RAF1	D001943	Breast Carcinoma
RAF1	D001943	Mammary Neoplasms, Human
RAF1	D001943	Breast Neoplasms
RAF1	D001943	Mammary Carcinoma, Human
RAF1	D002289	Carcinoma, Non-Small-Cell Lung
RAF1	D002289	Non Small Cell Lung Carcinoma
RAF1	D008175	Lung Neoplasms
RAF1	D008175	Lung Cancer
WNT3	D001281	Atrial Fibrillation
WNT3	D001281	Atrial Fibrillation, Paroxysmal
WNT3	D001281	Atrial Fibrillation, Persistent
WNT3	D001281	Atrial Fibrillation, Familial
PPARGC1A	D010300	Parkinson Disease
TXNL4A	D002972	Cleft Palate
TXNL4A	D002972	Cleft Palate, Isolated
KANSL1	D009123	Flaccid Muscle Tone
KANSL1	D009123	Muscle Hypotonia
KANSL1	D009123	Hypotonia, Muscle
KANSL1	D009123	Floppy Muscles
KANSL1	D009123	Muscle Tone Atonic
KANSL1	D009123	Unilateral Hypotonia
KANSL1	D009123	Neonatal Hypotonia
ATM	D009203	Myocardial Infarction
ERBB2	D008527	Medulloblastoma
ERBB2	D008527	Medullomyoblastoma
ERBB2	D008527	Childhood Medulloblastoma
ERBB2	D008527	Adult Medulloblastoma

ERBB2	D008527	Desmoplastic Medulloblastoma
ERBB2	D008527	Melanocytic Medulloblastoma
FGFR1	D055752	Small Cell Lung Carcinoma
FGFR1	D055752	Carcinoma, Small Cell Lung
KDR	D000230	Adenocarcinoma
KDR	D000230	Adenocarcinoma, Basal Cell
KDR	D000230	Adenocarcinoma, Oxyphilic
KDR	D000230	Carcinoma, Cribriform
KDR	D000230	Carcinoma, Granular Cell
KDR	D000230	Adenocarcinoma, Tubular
NPHS1	D007674	Kidney Diseases
GAB2	D009362	Neoplasm Metastasis
ATM	D006528	Carcinoma, Hepatocellular
RAF1	D005910	Glioma
RAF1	D005910	Gliomas, Mixed
RAF1	D005910	Malignant Glioma
SFTPD	D008175	Lung Neoplasms
SFTPD	D008175	Lung Cancer
KDR	D000077192	Adenocarcinoma of Lung
PTK2	D002294	Carcinoma, Squamous Cell
ERBB2	D008207	Lymphatic Metastasis
ERBB2	D006258	Head and Neck Neoplasms
ERBB2	D006258	Head Neoplasms
ERBB2	D006258	Neck Neoplasms
ERBB2	D006258	Cancer of Head and Neck
ERBB2	D006258	Cancer of Neck
ERBB2	D006258	Cancer of Head

ERBB2	D006258	Upper Aerodigestive Tract Neoplasms
ERBB2	D000077274	Nasopharyngeal Carcinoma
ERBB2	D000077274	Carcinoma, Nasopharyngeal
FGFR1	D005910	Glioma
FGFR1	D005910	Gliomas, Mixed
FGFR1	D005910	Malignant Glioma
ATM	D016411	Peripheral T-Cell Lymphoma
ATM	D016411	Lymphoma, T-Cell, Peripheral
ATM	D002292	Renal Cell Carcinoma
ATM	D002292	Carcinoma, Renal Cell
ATM	D002292	Carcinoma, Hypernephroid
ATM	D002292	Chromophobe Renal Cell Carcinoma
ATM	D002292	Sarcomatoid Renal Cell Carcinoma
ATM	D002292	Collecting Duct Carcinoma of the Kidney
ATM	D002292	Papillary Renal Cell Carcinoma
ATM	D003123	Hereditary Nonpolyposis Colorectal Neoplasms
ATM	D003123	Colorectal Neoplasms, Hereditary Nonpolyposis
ATM	D003123	Hereditary Nonpolyposis Colorectal Cancer
ATM	D003123	Lynch Syndrome
ERBB2	D010212	Papilloma
ERBB2	D010212	Papilloma, Squamous Cell
ERBB2	D010212	Papillomatosis
FGFR1	D003863	Depression
FGFR1	D003863	Depressive Symptoms
FGFR1	D003865	Involutional Depression
FGFR1	D003865	Depressive Disorder, Major
FGFR1	D003865	Major Depressive Disorder

FGFR1	D003865	Paraphrenia, Involutional
FGFR1	D003865	Psychosis, Involutional
NRG1	D003863	Depression
NRG1	D003863	Depressive Symptoms
NRG1	D003866	Depression, Endogenous
NRG1	D003866	Disorder, Depressive
NRG1	D003866	Depressive Disorder
NRG1	D003866	Melancholia
NRG1	D003866	Unipolar Depression
NRG1	D003866	Depressive Syndrome
NRG1	D003866	Depression, Neurotic
NRG1	D010146	Pain
NRG1	D010146	Pain, Burning
NRG1	D010146	Ache
NRG1	D010146	Pain, Radiating
NRG1	D010146	Pain, Splitting
NRG1	D010146	Pain, Crushing
NRG1	D010146	Pain, Migratory
NRG1	D010146	Suffering, Physical
KDR	D004938	Esophageal Neoplasms
KDR	D004938	Esophageal Cancer
NPHS1	D009402	Nephrosis, Lipoid
NPHS1	D009402	Glomerulonephritis, Minimal Change
NPHS1	D009402	Nephrotic Syndrome, Minimal Change
RAF1	C535579	Cardio-Facio-Cutaneous Syndrome
RAF1	C535579	Cardiofaciocutaneous syndrome
RAF1	D000077216	Carcinoma, Ovarian Epithelial

REV3L	D020331	Mobius Syndrome
GAB2	D008545	Melanoma
PPARGC1A	D012559	Schizophrenia
ATM	D012559	Schizophrenia
KDR	D020246	Venous Thrombosis
KDR	D020246	Deep Vein Thrombosis
RAF1	D008113	Liver Neoplasms
RAF1	D008113	Neoplasms, Liver
RAF1	D008113	Cancer of Liver
RAF1	D056685	Costello Syndrome
SETD1A	D002658	Developmental Disabilities
SETD1A	D002658	Child Development Deviations
SETD1A	D002658	Child Development Disorders, Specific
GPNMB	D015179	Colorectal Carcinoma
GPNMB	D015179	Colorectal Neoplasms
SIRT3	D002294	Carcinoma, Squamous Cell
TTC19	D019636	Degenerative Diseases, Central Nervous System
TTC19	D019636	Neurodegenerative Diseases
TTC19	D019636	Neurodegenerative Disorders
TTC19	D019636	Degenerative Diseases, Spinal Cord
AP2B1	D003863	Depression
AP2B1	D003863	Depressive Symptoms
ATM	D003528	Adenoid Cystic Carcinoma
ATM	D003528	Carcinoma, Adenoid Cystic
ERBB2	D009374	Neoplasms, Experimental
ERBB2	D002280	Carcinoma, Basal Cell, Pigmented
ERBB2	D002280	Carcinoma, Basal Cell

FGFR1	D001714	Bipolar Disorder
FGFR1	D001714	Depression, Bipolar
FGFR1	D001714	Manic Disorder
FGFR1	D001714	Mania
FGFR1	D006349	Heart Valve Diseases
FGFR1	D006349	Disease, Heart Valve
FGFR1	D003394	Craniofacial Dysostosis
FGFR1	D003394	Craniofacial Dysostosis Type 1
NRG1	D002189	Cannabis Abuse
NRG1	D002189	Cannabis Dependence
NRG1	D002189	Hashish Abuse
NRG1	D002189	Marijuana Abuse
NRG1	D002189	Cannabis-Related Disorder
KDR	D003928	Diabetic Nephropathies
KDR	D003928	Diabetic Nephropathy
KDR	D003928	Glomerulosclerosis, Nodular
KDR	D004833	Epilepsy, Temporal Lobe
KDR	D004833	Uncinate Epilepsy
KDR	D004833	Epilepsy, Benign Psychomotor, Childhood
KDR	D004833	Epilepsy, Lateral Temporal
KDR	D012559	Schizophrenia
KDR	D003865	Involutional Depression
KDR	D003865	Depressive Disorder, Major
KDR	D003865	Major Depressive Disorder
KDR	D003865	Paraphrenia, Involutional
KDR	D003865	Psychosis, Involutional
POLE	D013274	Gastric Cancer

POLE	D013274	Stomach Neoplasms
POLE	D013274	Gastric Cancer, Familial Diffuse
POLE	C564543	Intrauterine Growth Retardation, Metaphyseal Dysplasia, Adrenal Hypoplasia Congenita, And Genital Anomalies
POLE	C564543	Adrenal Hypoplasia, Cytomegalic Type
RAF1	D006930	Hyperalgesia
RAF1	D006930	Allodynia
RAF1	D006930	Hyperalgesia, Primary
RAF1	D006930	Hyperalgesia, Secondary
RAF1	D006930	Tactile Allodynia
RAF1	D006930	Hyperalgesia, Thermal
RAF1	D006930	Mechanical Allodynia
RAF1	D009202	Cardiomyopathies, Primary
RAF1	D009202	Myocardial Diseases, Secondary
RAF1	D009202	Cardiomyopathies
WNT3	D020522	Lymphoma, Lymphocytic, Diffuse, Poorly-Differentiated
WNT3	D020522	Lymphoma, Mantle-Cell
WNT3	D020522	Lymphoma, Lymphocytic, Intermediate
XRCC4	D012559	Schizophrenia
SIRT3	D016640	Gestational Diabetes
SIRT3	D016640	Diabetes, Gestational
TTC19	D009422	Nervous System Diseases
TTC19	D009422	Disorder, Nervous System
TTC19	D028361	Mitochondrial Diseases
TTC19	D028361	Electron Transport Chain Deficiencies, Mitochondrial
TTC19	D028361	Oxidative Phosphorylation Deficiencies
TTC19	D028361	Mitochondrial Respiratory Chain Deficiencies

KAT8	D012559	Schizophrenia
KAT8	D012640	Jacksonian Seizure
KAT8	D012640	Seizures
KAT8	D012640	Partial Seizures, Complex
KAT8	D012640	Seizures, Generalized
KAT8	D012640	Clonic Seizures
KAT8	D012640	Seizure, Visual
KAT8	D012640	Tonic Seizures
KAT8	D012640	Atonic Seizures
KAT8	D012640	Seizures, Somatosensory
KAT8	D012640	Seizures, Auditory
KAT8	D012640	Seizure, Olfactory
KAT8	D012640	Seizure, Gustatory
KAT8	D012640	Seizure, Vertiginous
KAT8	D012640	Seizures, Tonic Clonic
KAT8	D012640	Convulsion, Non Epileptic
KAT8	D012640	Single Seizure
KAT8	D012640	Atonic Absence Seizures
KAT8	D012640	Convulsive Seizures
KAT8	D012640	Seizures, Focal
KAT8	D012640	Seizures, Sensory
KAT8	D012640	Nonepileptic Seizures
KAT8	D012640	Convulsions
KAT8	D012640	Absence Seizures
KAT8	D012640	Epileptic Seizures
KAT8	D012640	Myoclonic Seizures
KAT8	D012640	Generalized Absence Seizures

KAT8	D008607	Idiocy
KAT8	D008607	Mental Retardation, Psychosocial
KAT8	D008607	Deficiency, Mental
KAT8	D008607	Intellectual Disability
ADORA2B	D009362	Neoplasm Metastasis
ADORA2B	D018805	Pyemia
ADORA2B	D018805	Septicemia
ADORA2B	D018805	Sepsis
ADORA2B	D018805	Severe Sepsis
ADORA2B	D015427	Reperfusion Injury
ATM	D002311	Cardiomyopathy, Dilated
ATM	D002311	Cardiomyopathy, Familial Idiopathic
ATM	D005355	Fibrosis
ATM	D005355	Cirrhosis
ATM	D011297	Prenatal Exposure Delayed Effects
ATM	D016410	Lymphoma, T-Cell, Cutaneous
ATM	D016410	Granulomatous Slack Skin
ATM	D002386	Cataract
ATM	D002386	Pseudoaphakia
ATM	D002386	Lens Opacities
ATM	D018487	Ventricular Dysfunction, Left
ATM	D055847	Lynch Syndrome II
ATM	C537243	Prostate cancer, familial
ATM	C537243	Prostate Cancer, Hereditary, 1
ATM	C535972	Colorectal cancer, hereditary nonpolyposis, type 1
CD38	D000419	Albuminuria
CDH3	D010048	Corpus Luteum Cyst

CDH3	D010048	Ovarian Cysts
CDH3	D005128	Eye Diseases
DCC	D008545	Melanoma
DCC	D017436	Kallmann Syndrome
DCC	D017436	Kallmann Syndrome 1
DCC	D017436	Kallmann Syndrome 2
DCC	D017436	Kallmann Syndrome, Type 3, Recessive
DCC	C564593	Gaze Palsy, Familial Horizontal, with Progressive Scoliosis
DCC	C564593	Familial Idiopathic Scoliosis Associated with Congenital Encephalopathy
ERBB2	D001661	Biliary Tract Neoplasms
ERBB2	D001661	Biliary Tract Neoplasm
ERBB2	D001661	Biliary Tract Cancer
ERBB2	D009360	Embolism, Tumor
ERBB2	D015674	Mammary Neoplasms, Animal
ERBB2	D015674	Animal Mammary Neoplasms
ERBB2	D015674	Mammary Carcinoma, Animal
ERBB2	D009364	Neoplasm Recurrence, Local
ERBB2	D018450	Disease Exacerbation
FGFR1	D006258	Head and Neck Neoplasms
FGFR1	D006258	Head Neoplasms
FGFR1	D006258	Neck Neoplasms
FGFR1	D006258	Cancer of Head and Neck
FGFR1	D006258	Cancer of Neck
FGFR1	D006258	Cancer of Head
FGFR1	D006258	Upper Aerodigestive Tract Neoplasms
FGFR1	D017674	Hypophosphatemia
FGFR1	D017689	Polydactyly

FGFR1	D018316	Gliosarcoma
FGFR1	D005909	Glioblastoma
FGFR1	D005909	Giant Cell Glioblastoma
FGFR1	D005909	Glioblastoma Multiforme
FGFR1	D019465	Craniofacial Abnormalities
FGFR1	C565129	Cutis Gyrata Syndrome of Beare And Stevenson
FGFR1	C538582	Pfeiffer type acrocephalosyndactyly
NRG1	D002471	Cell Transformation, Neoplastic
NRG1	D002471	Neoplastic Cell Transformation
NRG1	D003877	Contact Dermatitis
NRG1	D003877	Dermatitis, Contact
NRG1	D003877	Hypersensitivity, Contact
NRG1	D008106	Liver Cirrhosis, Experimental
NRG1	D009069	Movement Disorders
NRG1	D009069	Etat Marbre
NRG1	D009361	Neoplasm Invasiveness
NRG1	D011085	Polycystic Ovary Syndrome
NRG1	D011085	Sclerocystic Ovaries
NRG1	D013276	Stomach Ulcer
NRG1	D013276	Ulcer, Gastric
NRG1	D001171	Juvenile-Onset Still Disease
NRG1	D001171	Arthritis, Juvenile
NRG1	D001171	Arthritis, Juvenile Psoriatic
NRG1	D001171	Polyarthritis, Juvenile, Rheumatoid Factor Negative
NRG1	D001171	Polyarthritis, Juvenile, Rheumatoid Factor Positive
NRG1	D019969	Amphetamine-Related Disorders
NRG1	D019969	Amphetamine Addiction

NRG1	D019969	Amphetamine Abuse
NRG1	D066126	Cardiotoxicity
KDR	D010009	Dyschondroplasias
KDR	D010009	Melnick-Needles Syndrome
KDR	D010009	Multiple Epiphyseal Dysplasia
KDR	D010009	Osteochondrodysplasias
KDR	D010009	Schwartz-Jampel Syndrome
KDR	D010009	Spondyloepiphyseal Dysplasia
KDR	D010009	Van Buchem Disease
KDR	D010009	Spondyloepiphyseal Dysplasia Tarda, X-Linked
KDR	D010009	Schwartz-Jampel Syndrome, Type 1
KDR	D008114	Hepatoma, Morris
KDR	D008114	Hepatoma, Novikoff
KDR	D008114	Liver Neoplasms, Experimental
KDR	D008114	Experimental Hepatoma
KDR	D009389	Pathologic Neovascularization
KDR	D009389	Neovascularization, Pathologic
KDR	D003866	Depression, Endogenous
KDR	D003866	Disorder, Depressive
KDR	D003866	Depressive Disorder
KDR	D003866	Melancholia
KDR	D003866	Unipolar Depression
KDR	D003866	Depressive Syndrome
KDR	D003866	Depression, Neurotic
KDR	D014511	Uremia
KDR	D017202	Myocardial Ischemia
KDR	D002543	Cerebral Hemorrhage

NPHS1	D000419	Albuminuria
NPHS1	D020138	Hyperhomocysteinemia
NUP88	D015658	HIV Infections
NUP88	D015658	HIV Coinfection
POLE	D012878	Cancer of Skin
POLE	D012878	Skin Neoplasms
PTK2	D001172	Rheumatoid Arthritis
PTK2	D001172	Arthritis, Rheumatoid
PTK2	D002471	Cell Transformation, Neoplastic
PTK2	D002471	Neoplastic Cell Transformation
PTK2	D009062	Mouth Neoplasms
PTK2	D009062	Cancer of Mouth
PTK2	D009361	Neoplasm Invasiveness
RAF1	D007680	Kidney Neoplasm
RAF1	D007680	Kidney Neoplasms
RAF1	D007680	Cancer of Kidney
RAF1	C536231	Familial dilated cardiomyopathy
RAF1	C562839	Mesothelioma, Malignant
RAF1	C564342	Noonan-Like Syndrome With Loose Anagen Hair
REV3L	C531747	Congenital facial diplegia
SFTPD	D055371	Acute Lung Injury
WNT3	C562839	Mesothelioma, Malignant
WNT3	C536500	Tetraamelia multiple malformations
XRCC4	D002869	Autosome Abnormalities
XRCC4	D002869	Chromosome Aberrations
XRCC4	D003123	Hereditary Nonpolyposis Colorectal Neoplasms
XRCC4	D003123	Colorectal Neoplasms, Hereditary Nonpolyposis

XRCC4	D003123	Hereditary Nonpolyposis Colorectal Cancer
XRCC4	D003123	Lynch Syndrome
XRCC4	D055847	Lynch Syndrome II
XRCC4	C564694	LIG4 Syndrome
XRCC4	C535972	Colorectal cancer, hereditary nonpolyposis, type 1
AAAS	D011471	Prostatic Neoplasms
AAAS	D011471	Prostate Cancer
SETD1A	D004827	Epilepsy
SETD1A	D004827	Epilepsy, Cryptogenic
SETD1A	D004827	Aura
SETD1A	D004827	Awakening Epilepsy
SETD1A	D008607	Idiocy
SETD1A	D008607	Mental Retardation, Psychosocial
SETD1A	D008607	Deficiency, Mental
SETD1A	D008607	Intellectual Disability
GAB2	D009361	Neoplasm Invasiveness
GPNMB	D058186	Kidney Failure, Acute
GPNMB	D058186	Acute Kidney Insufficiency
GPNMB	D058186	Kidney Injury, Acute
GPNMB	D058186	Acute Kidney Injury
GPNMB	D008106	Liver Cirrhosis, Experimental
GPNMB	D017114	Liver Failure, Acute
PPARGC1A	D001913	Bowen's Disease
PPARGC1A	D009137	Muscular Dystrophy, Animal
PPARGC1A	D020961	Lewy Body Disease
PPARGC1A	C537948	Ceroid lipofuscinosis, neuronal 1, infantile
SIRT3	D009062	Mouth Neoplasms

SIRT3	D009062	Cancer of Mouth
TTC19	D007246	Infertility
TTC19	D007246	Subfertility
TTC19	D007246	Sterility, Reproductive
TTC19	D007888	Leigh Disease
TTC19	D007888	Encephalopathy, Subacute Necrotizing, Infantile
TTC19	D007888	Encephalopathy, Subacute Necrotizing, Juvenile
TTC19	D020233	Gait, Unsteady
TTC19	D020233	Gait, Spastic
TTC19	D020233	Gait, Shuffling
TTC19	D020233	Gait, Athetotic
TTC19	D020233	Charcot Gait
TTC19	D020233	Gait, Festinating
TTC19	D020233	Gait, Reeling
TTC19	D020233	Gait, Hemiplegic
TTC19	D020233	Gait, Scissors
TTC19	D020233	Duck Gait
TTC19	D020233	Gait, Rigid
TTC19	D020233	Gait, Broadened
TTC19	D020233	Gait, Stumbling
TTC19	D020233	Rapid Fatigue of Gait
TTC19	D020233	Gait, Drop Foot
TTC19	D020233	Marche a Petit Pas
TTC19	D020233	Gait, Hysterical
TTC19	D020233	Gait Disorder, Sensorimotor
TTC19	D020233	Gait Disorders, Neurologic
TTC19	D020233	Gait, Frontal

TTC19	D020233	Gait, Widebased
TTC19	C564021	Leigh Syndrome Due To Mitochondrial Complex I Deficiency
TTC19	C564961	Leigh Syndrome Due To Mitochondrial Complex II Deficiency
TTC19	C564962	Leigh Syndrome due to Mitochondrial Complex III Deficiency
TTC19	C564963	Leigh Syndrome due to Mitochondrial Complex IV Deficiency
TTC19	C564964	Leigh Syndrome due to Mitochondrial Complex V Deficiency
TTC19	C538590	Necrotizing encephalopathy, infantile subacute, of Leigh
NADSYN1	D000015	Abnormalities, Multiple
KAT8	D001321	Autistic Disorder
KAT8	D002312	Cardiomyopathy, Hypertrophic
KAT8	D002312	Hypertrophic Cardiomyopathy
KAT8	D002312	Cardiomyopathy, Hypertrophic Obstructive
KAT8	D006333	Heart Failure
KAT8	D006333	Heart Failure, Congestive
KAT8	D006333	Left-Sided Heart Failure
KAT8	D006333	Heart Failure, Right-Sided
KAT8	D006333	Myocardial Failure
KAT8	D006333	Heart Decompensation
KAT8	D018376	Cardiovascular Abnormalities
VTI1A	D000230	Adenocarcinoma
VTI1A	D000230	Adenocarcinoma, Basal Cell
VTI1A	D000230	Adenocarcinoma, Oxyphilic
VTI1A	D000230	Carcinoma, Cribriform
VTI1A	D000230	Carcinoma, Granular Cell
VTI1A	D000230	Adenocarcinoma, Tubular
KANSL1	D003528	Adenoid Cystic Carcinoma
KANSL1	D003528	Carcinoma, Adenoid Cystic

KANSL1	D019465	Craniofacial Abnormalities
KANSL1	D010051	Ovarian Neoplasms
KANSL1	D010051	Neoplasm, Ovarian
KANSL1	D010051	Ovarian Cancer
KANSL1	C566025	Leukemia, Megakaryoblastic, of Down Syndrome
KANSL1	C538045	Chromosome 17 deletion
KANSL1	C538045	Chromosome 17p Deletion Syndrome

Supplementary Table 13. Vitamin D associated regulatory interactions.

snp	trait	Target gene	**eQTL beta**	GWAS OR	GWAS beta (unit change)	Study accession	Source network
rs12785878	Vitamin D insufficiency	NADSYN1	0.280	NA	0.036	GCST005367	PD-causal network
rs4944958	Vitamin D insufficiency	NADSYN1	0.280	NA	0.259	GCST90020244	PD-causal network
rs3831470	Vitamin D insufficiency	NADSYN1	0.258	NA	0.580	GCST90101732	PD-causal network
rs200454003	Serum 25-Hydroxyvitamin D levels	NADSYN1	NA	NA	-0.087	GCST010144	PD-causal network
rs7938885	Serum 25-Hydroxyvitamin D levels	NADSYN1	0.263	NA	8.125 (z-score decrease)	GCST005782	PD-causal network
rs12800438	Serum 25-Hydroxyvitamin D levels	NADSYN1	0.263	NA	NA	GCST005782	PD-causal network
rs12278461	Serum 25-Hydroxyvitamin D levels	NADSYN1	0.256	NA	-0.129	GCST90000619	PD-causal network
rs12278461	Serum 25-Hydroxyvitamin D levels	NADSYN1	0.256	NA	-0.034	GCST90000621	PD-causal network
rs12278461	Serum 25-Hydroxyvitamin D levels	NADSYN1	0.256	NA	-0.106	GCST90000620	PD-causal network
rs12278461	Serum 25-Hydroxyvitamin D levels	NADSYN1	0.256	0.569	NA	GCST90244035	PD-causal network
rs2297991	Serum 25-Hydroxyvitamin D levels	VTI1A	0.146	NA	-0.013	GCST90000618	PD-causal network
rs1894100	Serum 25-Hydroxyvitamin D levels	NADSYN1	NA	NA	-0.102	GCST90000618	PD-causal network
rs1790373	Serum 25-Hydroxyvitamin D levels	NADSYN1	0.236	NA	-0.102	GCST90000618	PD-causal network
rs11233933	Serum 25-Hydroxyvitamin D levels	NADSYN1	-0.232	NA	-0.116	GCST90000618	PD-causal network

rs28435470	Serum 25-Hydroxyvitamin D levels	POLE	-0.387	NA	-0.012	GCST90000618	PD-causal network
rs10793129	Serum 25-Hydroxyvitamin D levels	DGAT2	-0.286	NA	-0.024	GCST010144	PD-associated network
rs200454003	Serum 25-Hydroxyvitamin D levels	NADSYN1	NA	NA	-0.087	GCST010144	PD-associated network
rs7938885	Serum 25-Hydroxyvitamin D levels	NADSYN1	0.263	NA	8.125 (z score decrease)	GCST005782	PD-associated network
rs12800438	Serum 25-Hydroxyvitamin D levels	NADSYN1	0.263	NA	NA	GCST005782	PD-associated network
rs12278461	Serum 25-Hydroxyvitamin D levels	NADSYN1	0.256	NA	-0.106	GCST90000620	PD-associated network
rs12278461	Serum 25-Hydroxyvitamin D levels	NADSYN1	0.256	NA	-0.129	GCST90000619	PD-associated network
rs12278461	Serum 25-Hydroxyvitamin D levels	NADSYN1	0.256	NA	-0.034	GCST90000621	PD-associated network
rs12278461	Serum 25-Hydroxyvitamin D levels	NADSYN1	0.256	0.569	NA	GCST90244035	PD-associated network
rs2297991	Serum 25-Hydroxyvitamin D levels	VTI1A	0.146	NA	-0.013	GCST90000618	PD-associated network
rs72997623	Serum 25-Hydroxyvitamin D levels	DGAT2	-0.281	NA	0.029	GCST90000616	PD-associated network
rs1894100	Serum 25-Hydroxyvitamin D levels	NADSYN1	NA	NA	-0.102	GCST90000618	PD-associated network
rs1790373	Serum 25-Hydroxyvitamin D levels	NADSYN1	0.236	NA	-0.102	GCST90000618	PD-associated network
rs11233933	Serum 25-Hydroxyvitamin D levels	NADSYN1	-0.232	NA	-0.116	GCST90000618	PD-associated network
rs3060	Serum 25-Hydroxyvitamin D levels	DGAT2	-0.245	NA	0.028	GCST90000618	PD-associated network

Supplementary Table 14. IDUA associated regulatory interactions.

snp	trait	Target gene	**eQTL beta**	GWAS beta (unit change)	Study accession	Source network
rs3822020	Alpha-L-iduronidase levels (IDUA.3169.70.2)	TMEM175	0.145	0.61 unit increase	GCST90240249	PD-causal network
rs3822020-A				0.573546 unit decrease	GCST90161644	PD-causal network
rs4690203	Alpha-L-iduronidase levels (IDUA.3169.70.2)	TMEM175	-0.386	0.46 unit decrease	GCST90240249	PD-causal network
rs4690203	Alpha-L-iduronidase levels (IDUA.3169.70.2)	GAK	0.185	0.46 unit decrease	GCST90240249	PD-causal network
rs13101828	Alpha-L-iduronidase levels (IDUA.3169.70.2)	TMEM175	NA	0.46 unit increase	GCST90240249	PD-causal network
rs13101828	Alpha-L-iduronidase levels (IDUA.3169.70.2)	GAK	-0.151	0.46 unit increase	GCST90240249	PD-causal network
rs56079856	Alpha-L-iduronidase levels (IDUA.3169.70.2)	GAK	0.177	0.38 unit increase	GCST90240249	PD-causal network
rs56079856	Alpha-L-iduronidase levels (IDUA.3169.70.2)	GAK	0.177	0.38 unit increase	GCST90240249	PD-associated network
rs13101828	Alpha-L-iduronidase levels (IDUA.3169.70.2)	GAK	-0.151	0.46 unit increase	GCST90240249	PD-associated network
rs4690203	Alpha-L-iduronidase levels (IDUA.3169.70.2)	GAK	0.185	0.46 unit decrease	GCST90240249	PD-associated network

Supplementary Table 15. Melanoma associated regulatory interactions.

snp	trait	Target gene	**eQTL** beta	GWAS OR	GWAS beta (unit change)	Study accession	Source network
rs910873	Melanoma	CHMP4B	0.2211824	1.75	NA	GCST004142	PD-causal network
rs17401449	Melanoma	CHMP4B	0.2402662	1.3089005	NA	GCST90011809	PD-causal network
